# Plant diversity and community analysis of Sele-Nono forest, Southwest Ethiopia: implication for conservation planning

**DOI:** 10.1186/s40529-022-00353-w

**Published:** 2022-07-19

**Authors:** Alemayehu Kefalew, Teshome Soromessa, Sebsebe Demissew

**Affiliations:** 1 Department of Plant Biology and Biodiversity Management, the National Herbarium, Addis Ababa University, P. O. Box 3434, Addis Ababa, Ethiopia; 2 Center for Environmental Science, Addis Ababa University, P. O. Box 1176, Addis Ababa, Ethiopia; 3 Department of Biology, Debre Markos University (DMU), P. O. Box 269, Debre Markos, Ethiopia

**Keywords:** Canonical correspondence analysis, Cluster analysis, Community types, Multivariate technique, Ordination, Sele-Nono Forest

## Abstract

**Background:**

Studying the floristic diversity of a certain forest is a basic aspect of the design and management of forest vegetation; and consequently this study focused on the plant diversity and community analysis of the Sele-Nono forest. For the current study, plants were sampled from 90 plots using a stratified random sampling technique along the established strata of the study forest. In all the plots, both floristic and environmental data that were relevant to the study were collected following the state of the art. Based on the collected data, the community types, ordination, floristic diversity, and threats to the forest were analyzed using R-package and SPSS software.

**Results:**

Cluster analysis produced seven distinct community types which significantly differed among themselves (Cophentic correlation coefficient = 0.785, P < 0.001) of which community types 2 and 6 were relatively poor; whereas communities 1 and 4 were rich in terms of their species richness and diversity. In addition, Canonical Correspondence Analysis (CCA) suggests that a number of environmental factors such as altitude and slope (topographic factor), OM and N (edaphic factors) and disturbance were the main drivers for the current distribution of plant species and disparity in plant community composition in Sele-Nono forest. Moreover, the study revealed high beta diversity (${\beta }_{w}$ >12) of plant species at the landscape level (i.e., throughout the study forest). Deforestation for agricultural land expansion and degradation through selective logging are the main threats to the Sele-Nono forest.

**Conclusions:**

The present study revealed that the Sele-Nono forest is a large and heterogenous forest at the landscape level (150, 325.27 ha; ${\beta }_{w}$ >12). Moreover, it is one of the richest and diverse forest ecosystems in terms of plant biodiversity, and it could qualify to be labeled as a keystone ecosystem. However, currently it is exposed to a variety of threats. We recommend the forest to be developed into a biosphere reserve. We also recommend the prioritization of areas belonging to community types 2 and 6 of the forest for any possible conservation actions so as to maximize species richness and diversity of the native plants of the area.

**Supplementary Information:**

The online version contains supplementary material available at 10.1186/s40529-022-00353-w.

## Introduction

Forest, according to FAO (2020), is defined as segments of a land spanning more than 0.5 hectares with trees higher than 5 m and a canopy cover of more than 10%, or trees able to reach these thresholds in situ. Forests are crucial habitats for hosting diverse organisms and providing ecosystem services (Wilson 1992; Pearce and Pearce 2001; Wilson et al. 2012; Brockerhoff et al. 2017). Globally, they are the dominant ecosystem of the world and cover approximately 30% of the planet’s land area (Olson and Dinerstein 1998; Olson et al. 2001; FAO, 2010). Of the global forests, tropical forests are believed to be the most species-rich ecosystem on earth although it covers only about 5% of the Earth’ s surface (Mutke and Barthlott 2005; World Conservation Monitoring Centre (WCMC 1992); Wright and Muller-Landau 2006; Elliot et al. 2013; Wilson et al. 2012). The tropical forests are mainly distributed in tropical landmasses such as America, Asia, and Africa which are situated between the tropic of Cancer and the tropic of Capricorn (Thomas and Boltzer 2002).

It is said that about 30% of the tropical forest cover is found in Africa, which is accounting for 21% of its total land area and for 16% of the global forest coverage (Whitmore 1997; Richards 1996; Whitmore 2008; Klopper et al. 2002; FAO 2006). Among the tropical forest ecosystems of Africa, its Afromontane forests are the most species-rich ecosystems housing more than half of African’s flora (Sayer et al. 1992; Kuper et al. 2004). These Afromontane forests are distributed along with the mountain spots of Cameroon and Sierra Leone in the West to the Ethiopian highlands and the Somalia Ahl Muscat Mountains in the East; and from the Red Sea Hills in the north to the Cape region in the south (White 1978; Friis 1992; Linder et al. 2005). More than 50% of these Afromontane forests are found in Ethiopia (Yalden 1983).

Although there is controversy over the precise figure of the former forest cover in Ethiopia, it is thought that about 35–40% of the land area has been covered with forests (EFAP 1994). However, by the 1950s the high forests declined to 16% of the total land area (Breitenbach 1963) which furthermost rapidly dropped to 3.6% by the 1980s (Egziabher 1991). In 2003, the forest cover was estimated to be 2.3% (Tedla 2003; WBISPP 2004; FAO 2006). By now even if the country currently increased its forest coverage due to the new FAO forest definition, the natural forest cover still decline to less than 2% (Kelbessa/Member of the task force in FAO/, Pers. Comm). The remaining major dense forests are found in the moist highlands of Southwest (SW) Ethiopia (MOA 1990; Reusing 1998; Friis et al. 2011).

Given the existed access restrictions of the SW forest due to its unique physical setting, climatic conditions, and biogeographical position, the area was not to the attention of researchers for a long time in the history of botanical explorations compared to other corners of Ethiopian vegetation (Meyer 1965; Chaffey 1978b; Friis 2002; Teketay 2004; Friis 2009). However, by the mid-1900s, which is often considered as a renaissance period by the botanists due to access roads (Tadesse 1993, 1994; Friis 2011), some pioneered floristic work on the area became evident (Logan 1946; Chaffey 1978a, 1979; Friis 1979; Friis et al. 1982; 1986a, b). The outcome of these studies discovered the area to have ecological and economic potential and attracted the attention of many other researchers for further studies. The research effort of the Ethiopian botanist and related stakeholders were instrumental in providing additional data to ‘Conservation International’ to consider the area as part of the Eastern Afromontane hotspot so as to give due recognition for being a globally key area for biodiversity conservation(WWF and IUCN 1994; Barthlott et al. 1999; Kuper et al. 2004; Mittermeier et al. 2004; Conservation International 2006; Vivero et al. 2006).

From these early efforts on, vegetation scientists did specific floristic studies and generated enormous data on the diversity, distribution, and conservation status of the existing patches of the area. Some of these studies include the work of Derero (1998), Kigomo (1999); Yeshitela and Bekele (2002), Woldemariam (2003), Yeshitela and Bekele (2003), Bekele (2003), Woldu and Yeshitela (2003), Berhan and Assefa (2004), Yeshitela and Shibru (2004), Ersado and Assefa (2004), Bekele et al. (2004), Senbeta et al. (2005), Denu (2006), Senbeta (2006), Schmitt (2006), Senbeta et al. (2007), Kelbessa and Soromessa (2008), Hundera and Deboch (2008), Hundera and Gadissa (2008), Woldemariam et al. (2008); Nune (2008), Gobeze et al.(2009), Schmitt et al. (2010), Friis et al. (2011), Assefa et al. (2013), Hylander et al.(2013a,b), Soromessa and Kelbessa (2013), Gebrehiwot and Hundera (2014), Mulugeta et al. (2015), Addi et al. (2016), Siraj et al. (2017), Yasin et al. (2018), Denu (2019), Addi et al. (2020), Dibaba et al. (2022). Despite these and many other related studies, the area still faces the challenge to conserve its vegetation and is becoming among the highly-threatened ecosystems in Ethiopia. To protect these fragile ecosystems through designing and successfully implementing more appropriate practices it primerly requires complete inventory on the floristic composition and diversity of plants in the area so as to represent ecological changes that may occur over years (Kneeshaw et al. 2000; Zumeta and Ellefson 2000; Adams and Hulme 2001; Larsson and Danell 2001; Bekele 2004; Schmitt et al. 2010). Thus, it would be imperative to conduct further floristic studies for each patch of the SW forest particularly in places that had not been addressed previously.

Literature shows that most of the floristic studies conducted so far in the moist southwest forest are concentrated on the forest fragments of Jimma Zone (from Oromia regional state), Keffa, Sheka, and Benchi Maji Zone (from the South Nation Nationalities and People Regional, (SNNPR) state and Godere District Forest (from Gambella regional state). Studies on the moist forests of Illuababora Zone (Oromia regional state) are very scanty although they largely contribute to the total forest coverage of the SW forest. With this drive, Sele-Nono Forest, which is the largest intact natural forest in Illuababora Zone, was entertained in this study with emphasis on the diversity and community analysis of plants as such information provides the baseline data about the current status of the forest.

## Statement of the problem

Sele-Nono forest is registered as one of the forest priority areas of the country which falls within the boundaries of Sele-Anderacha national forest priority areas (NFPAs) (EFAP 1994) even if its botanical wealth was not thoroughly assessed by botanists. Moreover, the forest is believed to be one of the few jungle forests in the country with only 5% of it occupied by non-forest land; and hence studying this area would help to design proactive conservation strategies before the area is getting seriously threatened by human impacts as it is economically, climatically and ecologically sensitive. On top of this, the area is a remarkable natural laboratory to investigate the ecological relationships among the vegetation and environment (ecological factors) so as to have a better understanding of species diversity, which is a key parameter to develop appropriate forest conservation measures. Hence, this study was initiated for all these purposes.

## Objectives

### General objective

The general objective of this study aims at understanding the plant diversity and community analysis of the Sele-Nono forest.

### Specific objectives

The specific objectives were (i) to describe plant community types of Sele-Nono forest and their ecological relationships with some environmental factors (ii) to assess the extent of plant diversity within and along the described plant community types (iii) to identify and rank the existing threats of the forest and recommend possible management directions to prevent from quick rupture.

## Material and methods

### Description of the study area

**Fig1:**
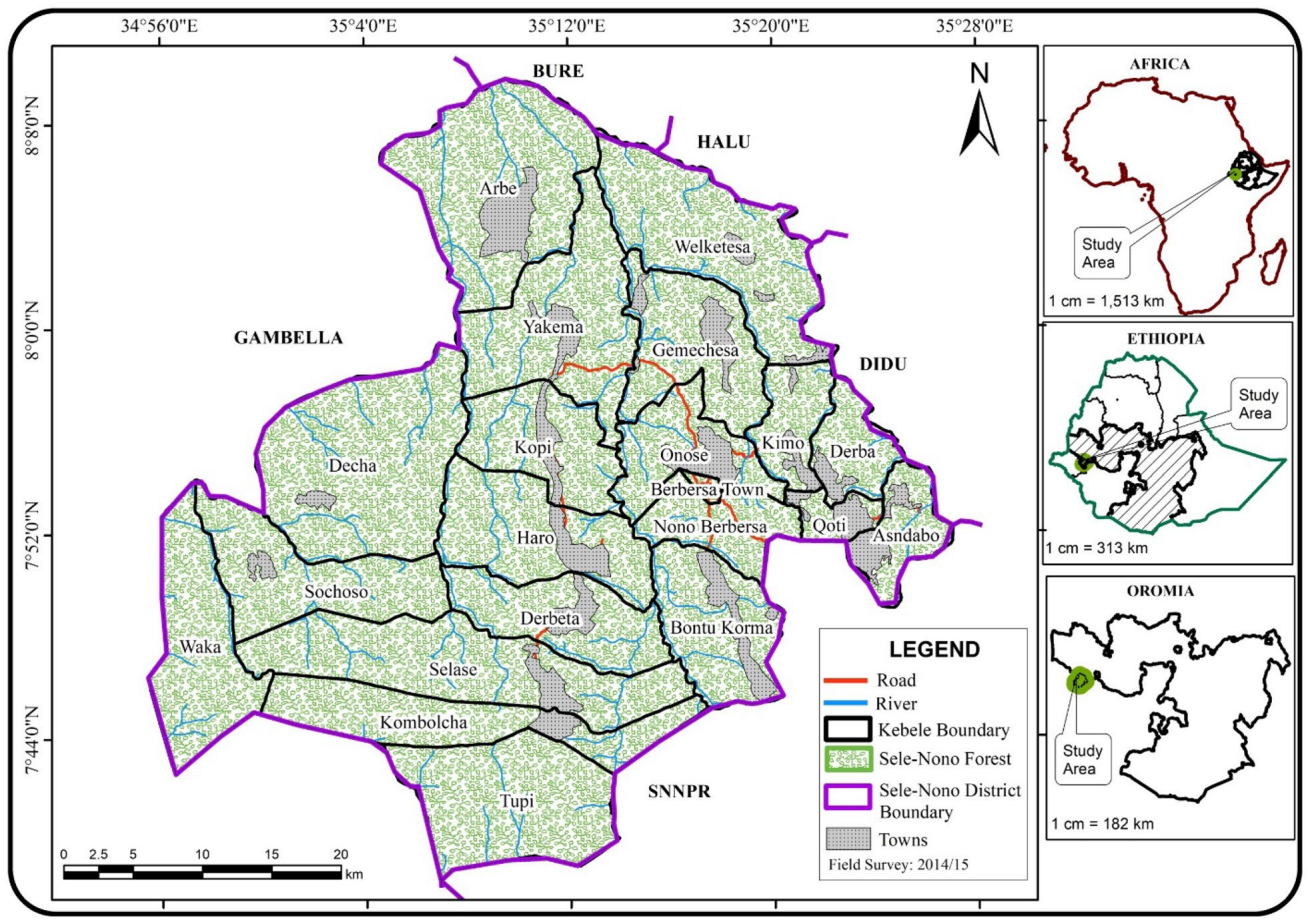
Map of Africa and Ethiopia showing the study area

The study was carried out in the Sele-Nono forest, Southwest Ethiopia (between 7^o^38^′^–8^o^10′N and 34^o^56^′^–35^o^26′E). It is located in the Sele-Nono District of Illuababora Zone of Oromia Regional State (Zone is an administrative unit in Ethiopia). The district has a total area of 260,000 ha, of which 95% of it is covered by natural forest and it is divided into twenty administrative Kebeles, the smallest administrative units, namely Arbe, Asendabo, Bontu Korma, Decha, Derba, Derbeta, Gemeches, Haro, Kimo, Kombolcha, Kopi, Nono Berbersa, Onose, Qoti, Selase, Sochoso, Tupi, Waka, Welketesa and Yakema. The study area is characterized by undulating terrain with an altitude range of 840 m asl to 2448 m asl. The climate is monsoon-like (i.e., tropical moist climate type with heavy summer rains) with a mean annual precipitation of 1620 mm (concentrated March to December) and a mean annual temperature of 21 °C (Asres 1996). According to the Sele-Nono District Agricultural Organization (SNDAO) soils of the area mainly belong to nitosol group with a pH range from 6.4 to 6.6 (SNDAO 2015). The vegetation of the area falls into the transitional rainforest and Afromontane rainforest types (Friis 1992). The dominant vegetation in the transitional rainforest of the area includes *Pouteria atissima, Anthocleista schweinfurthii, Manilkara butugi, Morus mesozygia, Strychnos mitis, Trichilia dregeana,* and *Trilepisium madascariense*; whereas *Pouteria (Aningeria)-adolfi-friederici, Syzygium guineense, Olea welwitschii, Schefflera abyssinica, Croton macrostachyus, Ilex mitis* and the likes are very dominant to the Afromontane forest part of the study area (Friis et al. 2011). The studied forest is bordered on the Southwest by Gambela region, on the North by Bure, on the Northeast by Halu, and on the Southeast by the Southern Nations, Nationalities, and Peoples Region (SNNPR) (Fig. 1).

## Research methods

### Sampling design

Preliminary field investigations at Sele-Nono forest in March 2013 have enabled the researcher to employ a stratified random sampling technique for the study. For this purpose, the study area was initially developed using the Digital altitude model (DEM) technique extracted from SRTM (Shuttle Radar Topography Mission) database which has approximately 30 m spatial resolution (Farr and Kobrick 2000). Then it was stratified into six strata following the maximum recommended strata number suggested by MacDicken (1997) based on 275 m altitude differences, and sampling plots were allocated proportionally along with each stratum (Table 1 below).

**Table 1:** Stratification of the study area and total number of plots allocated to each stratum

Strata No.	Altitude range for each strata (m, asl)	Total area (forest and village) of each strata (ha)	Total village areas in each strata (Ha)	Total net forest areas in each strata (Ha)	Proportion of forest areas in each strata (%)	Total number of plots allocated to each strata
stratum 1	2182–2448	18,490.44	117.35	18,373.08	12.22	11
stratum 2	1994–2181	42,025.49	5279.31	36,746.17	24.44	22
stratum 3	1646–1993	45,673.67	8927.49	36,746.17	24.44	22
stratum 4	1378–1645	33,827.22	421.61	33,405.61	22.22	20
stratum 5	1109–1377	23,637.48	253.55	23,383.93	15.55	14
stratum 6	840–1108	1670.28	0	1670.28	1.11	1
Total	840–2448	165,324.60	14,999.33	150,325.27	100	90

**Fig. 2:**
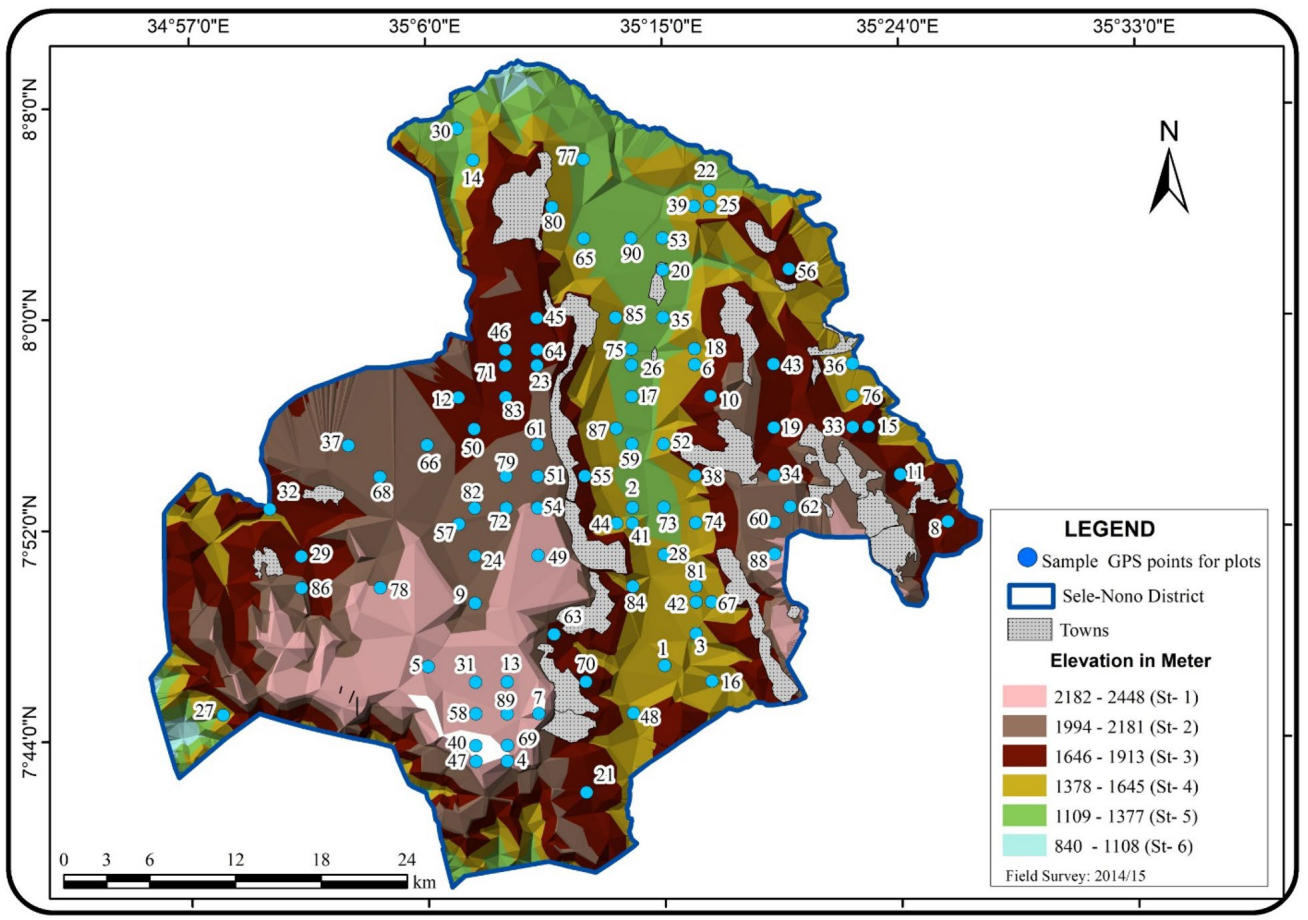
Location of permanent sample plots in the study area. Numbers on the map are plot numbers

The allocation of sampling points was done by making geographical grids on the map of the area before going to the field. The grids were made by dividing the map of the study forest in latitude and longitude coordinate using a one-minute (1’) grid-scale, which is equivalent to 1.85 km actual distance on the ground. Then some of the intersection points of the grids from each stratum were randomly chosen as a sample point (sample plot) using the lottery method, and plot numbers were given randomly to each of the sampling points using the same lottery technique (Additional file 1: Appendix S1). Those plot locations on the map (Fig. 2) were placed on the ground of the actual study area using GPS navigation after ridding to the nearest town. Sampling was conducted after checking any category of natural or semi-natural vegetation in the stratum. Agricultural fields and plantations were not sampled.

### Vegetation survey

**Fig. 3:**
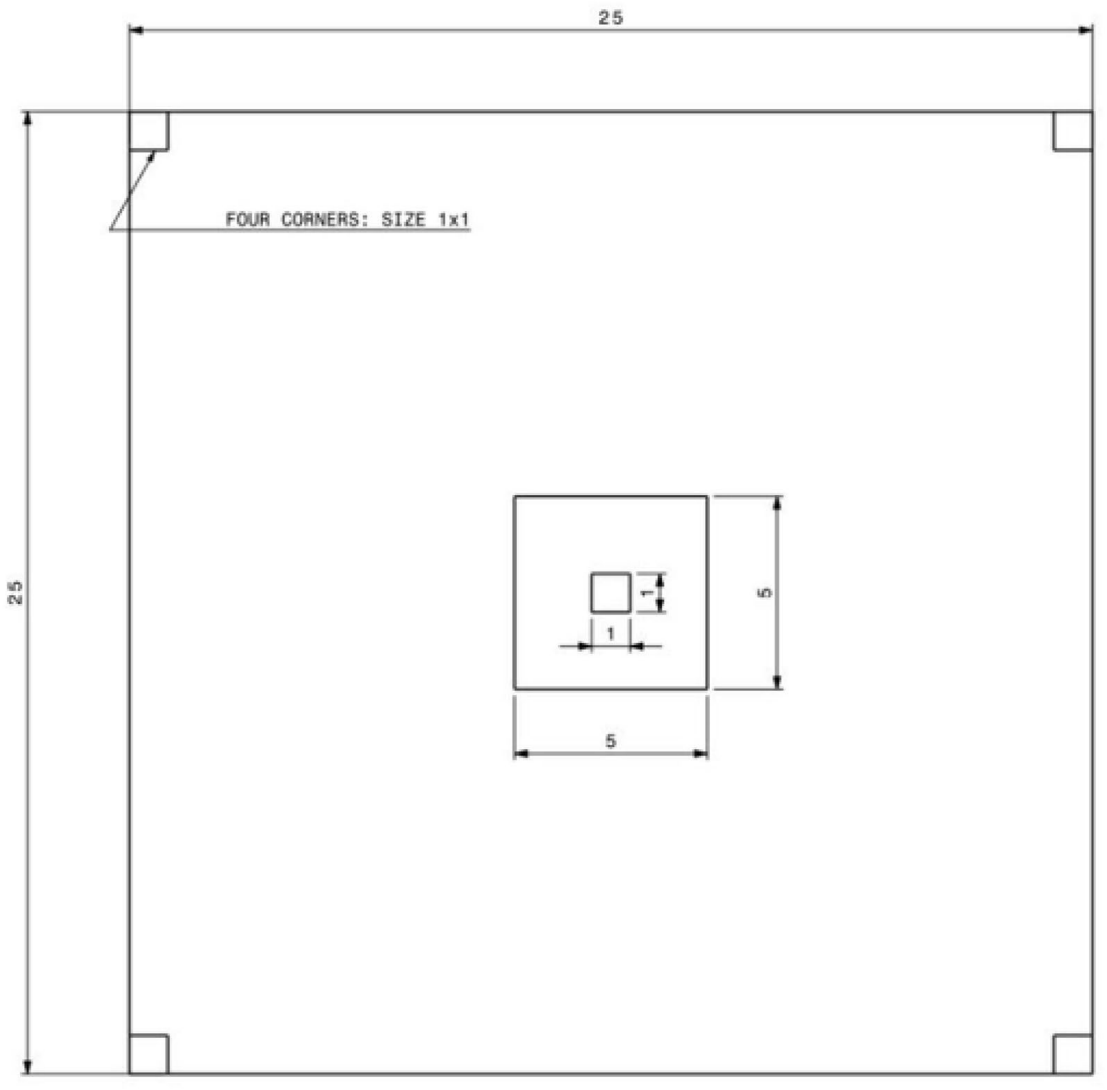
Design of plot layout in the study area (unit in meter, scale 1:600)

Field data on the vegetation at Sele-Nono forest was carried out following the releve approach (Braun-Blanquet 1932; Van der Maarel 1979; Podani 2006) from January 2014 to December 2016 escaping the field activities each year between the mid-March to mid-October because of heavy rain on the area. Considering the size of the forest, variation of vegetation, and environmental factors a total of 90 (25 m by 25 m) permanent plots were established in the study area. Plot size was determined using the published table by Van der Maarel (2005) and based on previous experience from other SW forests of Ethiopia since the minimum area method was difficult to map in the study area. The practical use of a minimum area curve for plot size determination was also criticized for moist tropical forests (Kent and Coker 1992; Van der Maarel 2005). The 25 m by 25 m plot size was adopted in this study as this size is a trade-off between the 20 m by 20 m (Woldemariam et al. 2002; Senbeta et al. 2014) and 30 m by 30 m (Kelbessa and Soromessa 2008; Mulugeta et al. 2015) plot size, which were the most widely used plot sizes for floristic study in SW forests of Ethiopia. All woody individuals with DBH > 2.5 cm and rooted within the quadrat were recorded in each plot. Canopy cover (the percentage of ground area covered or shaded) of all trees (> 1.5 m in height) were assessed in the entire plot (25 m × 25 m), while shrubs were measured in 25 m^2^ subplot designed in the center of the main plot. Cover of herbaceous plant species was surveyed in five 1m^2^ quadrats within each sample area; four of which were arranged at the four corners of the main plot and one was at the center of the 25 m^2^ subplot (Fig. 3). The cover-abundance for herbaceous species was estimated by calculating the average cover-abundance of each herb species existing in five subplots established inside a large quadrat. Then, the abundance and/or percentage cover estimates recorded in the field were converted into cover-abundance values (Additional file 2: Appendix S2) according to 1–9 modified Braun-Blanquet ordinal scale (Van der Maarel 2005). Plant species that were not parts of the plots (for trees) or subplots (for shrubs and herbs) but encountered during our field walk were also collected and recorded only as ‘present’ to enrich the floristic composition of the study area; but not included further in the data analysis (Mueller-Dombois and Ellenberg 1974).

### Environmental data collection

The environmental factors (altitude, slope, slope orientation (Aspect), disturbance, and soil characteristics) were determined for each of the main plots so as to explain possible differences in plant communities by their corresponding environmental factors. Disturbance on each plot was estimated subjectively on ordinal scales from zero to five following checklist developed for this study (Additional file 3: Appendix S3). GPS was used to record the altitude and position (latitude and longitude) of the sampled plots. Compass and Clinometer were used to measure aspect and slope respectively. The aspect was codified according to Woldu et al. (1989). Composite soil samples were collected at a depth of 30 cm by pooling soil samples from all five subplots. These soil samples were air-dried; and analyzed for total nitrogen (N), available phosphorus (P), organic matter (OM), pH, Potassium (K), Calcium (Ca), Magnesium (Mg), Cation exchange capacity (CEC), and texture (sand, silt, and clay) at the National Soil Test Laboratory of Ethiopia.

### Interview

Semi-structured interviews using checklists prepared in advance (Additional file 4: Appendix S4) were made with the informants of the local people to understand the threats of the forest understudy. For this purpose, a total of 40 informants who have been born or have lived most of their lives in the study area were chosen. 30 of the informants (5 from each stratum) were randomly selected following Kefalew et al. (2015). The other 10 of the total informants were key informants who were believed to have immense knowledge of the past and present change of the forest; and they were selected based on the recommendation from the local people, local authorities, and development agents (DAs) in the study area.

### Plant identification

Plant species that were easily identifiable were recorded on the field. Other species were temporarily stored in a plastic bag; and then pressed and brought to the National Herbarium (ETH) of Addis Ababa University (AAU) where they were dried, deep-frozen, and identified. The identifications were first performed using Natural database for Africa (2011) and keys of published volumes of Flora of Ethiopia and Eritrea (Edwards et al. 1995; Hedberg and Edwards 1989; Edwards et al. 1997; Edwards et al. 2000; Hedberg et al. 2006; Hedberg et al. 2009) and later supported with identification by comparisons with the already authenticated dried specimen in the Herbarium. At last, all the Latin names of the plant species were confirmed by taxonomic experts in Addis Ababa University (AAU).

### Data analysis

**Fig. 4:**
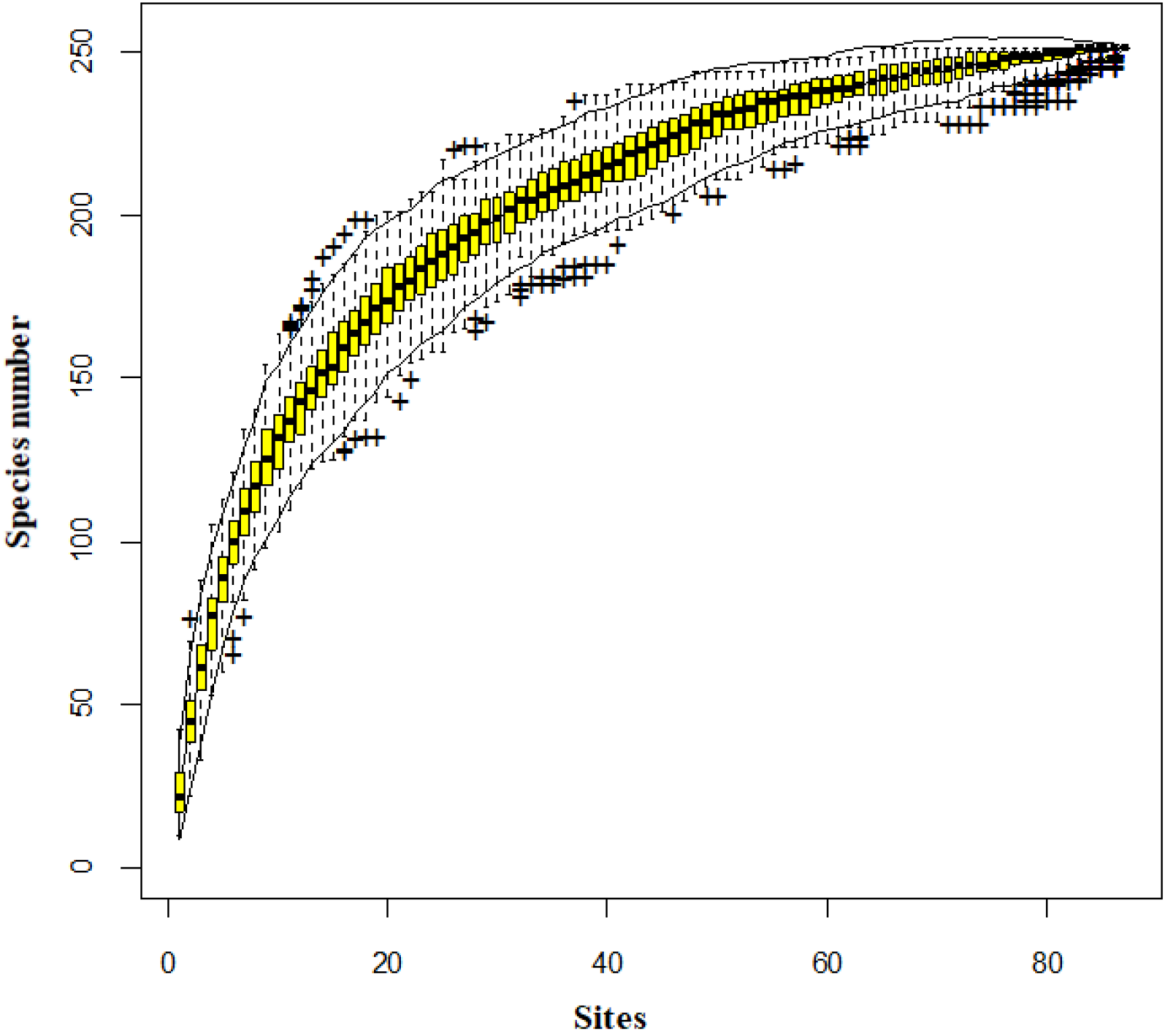
Species accumulation curve showing the relationship between the numbers of sites or quadrats and the number of plant species in Sele-Nono Forest

A species accumulation curve (SAC) of the data was drawn to ensure that we had collected an adequate number of plots for the study using “library (vegan)” package in R (Oksanen et al*.* 2011) (Fig. 4).

**Fig. 5:**
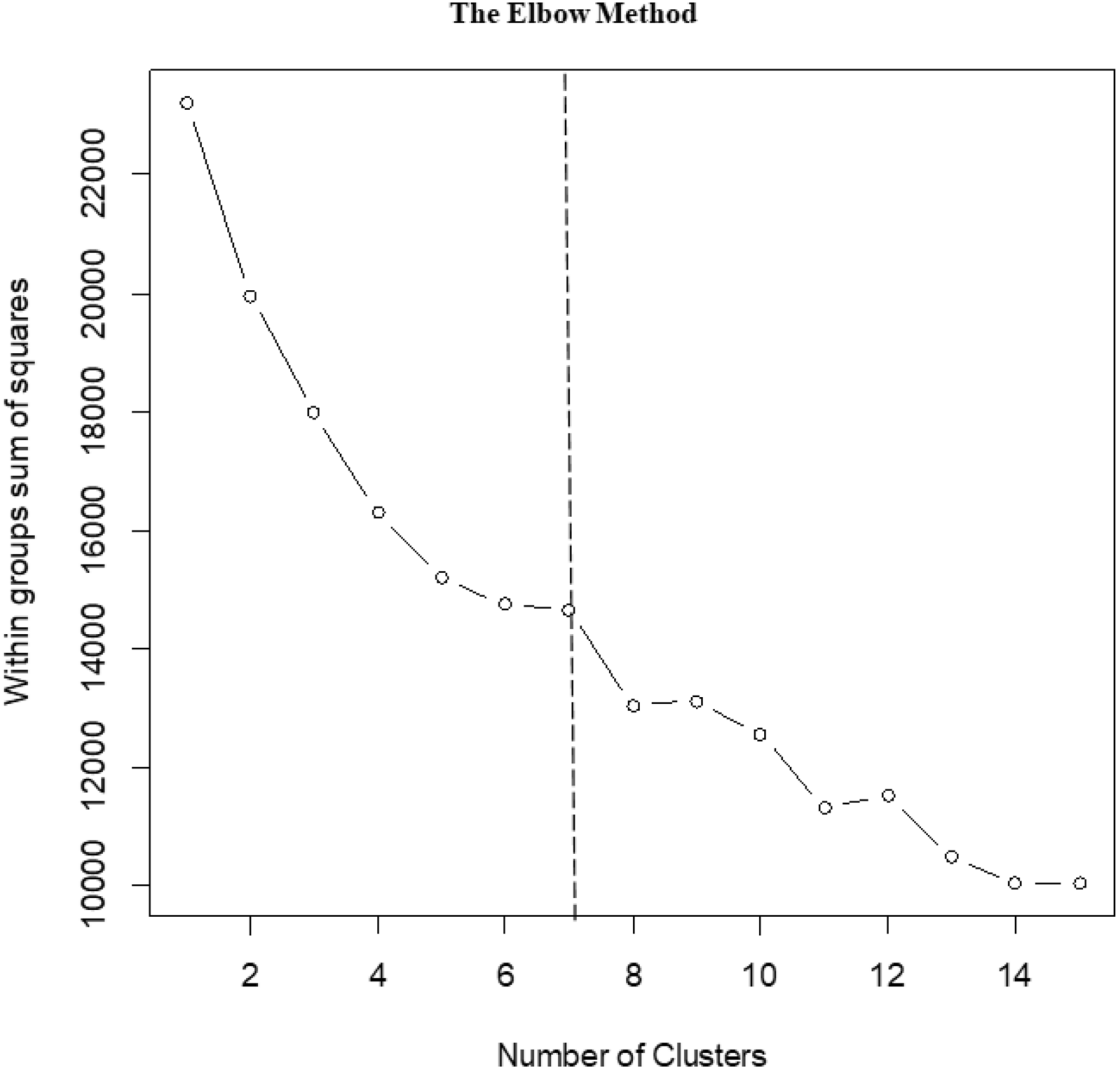
Determination of optimal number of clusters corresponding to plant community types in Sele-Nono forest (Diagram of the Elbow method shows the expected optimal number of clusters. Within group (Cluster) Sum of Squares values represent the sum of squared Euclidean distances between the plots and the centroid. The Elbow is the first breaking point, seen after the flattening of the curve, in our case, 7)

Agglomerative hierarchical cluster analysis using dendrogram was performed for recognizing and describing plant community types as this tool is the most recommended method of classification in synecology (Van der Mareel 2005). For this purpose, the optimum number of clusters (K) was specified first and foremost using the elbow method by employing a program developed for this purpose in R (R Development Core Team 2011). This method is a graphical representation of the total intra-cluster variation [i.e., total within group or cluster sum of square (WSS)] as a function of the number of clusters (Fig. 5); and the optimum cluster number is quickly judged by inspecting the elbow (first breakpoint or bend) of the plotted curve with the assumption that adding another cluster doesn’t improve much better the total WSS (Woldu 2017). Researchers on the area suggest this elbow method as a fairly clear method, if not a naive solution as it is based on intra-cluster variance (Chebaeva et al. 2020; Kongphunphin and Srivanit 2021; Nalli et al. 2021).

Then the dendrogram was performed using Euclidean distances with the ‘cluster’ and ‘gclus’ packages in R. Euclidean distances was also used in other studies to develop the dendrograms in cluster analysis (Senbeta 2006; Woldemariam et al. 2002; Kebede et al. 2013). The agglomerative strategy used in grouping the clusters was Ward’s method as it is the most recommended clustering strategy to produce reasonably compact clusters (McCune and Grace 2002; Woldu 2012). The cophenetic correlation coefficient, which is regarded as a criterion of successful classification, was calculated to infer the goodness of fit of the dendrogram. Synoptic table analysis was used to name and describe community types.

Outlier analysis was conducted to drop out rare species from the analysis (species which occur three or fewer times in the data matrix) using ‘the function dropspc in the library labsdv’ (Roberts 2010) as rare species are noise to the cluster analysis. The abundance data of the remaining plants were Hellinger transformed prior to analyses to reduce skewness (Woldu 2012; Aerts et al. 2006). The normality of the environmental variables was checked visually using histograms and with the Shapiro–Wilk normality test. Based on this analysis all the measured environmental variables except the altitude, slope, aspect, and sand data were log10 (x + 1) transformed as the transformation of variables would not affect the outcome of CCA. Mantel test was first applied to see if there were a significant correlation between the species composition and environmental variables (Legendre et al. 2015). Constrained ordination technique was then used to display the relative contribution of the measured environmental variables using canonical correspondence analysis (CCA) since a gradient length (GL) on a preliminary detrended correspondence analysis (DCA) showed that the species respond unimodal (GL of 4.07 > 4SD) to environmental gradients (Leps and Smilauer 2003). The environmental variables that were only significant in a forward selection procedure (Monte Carlo permutation tests, n = 999) were considered important in explaining the floristic composition of the study area and thus included on the CCA diagram. All data processing was performed using R (R Development Core Team 2011).

Plant diversity measures such as Shannon and Wiener (1949) index of species diversity (H’), richness (S), and evenness (J) were computed in R (R Development Core Team 2011) to analyze the status of plant diversity in the established plant community types (Kent and Coker 1992; McCune and Grace 2002); and to infer the impact of human being on the spatial distribution of the vegetation (Magurran 2004; Leitner and Turner 2001). Moreover, Whittaker alpha and beta diversity was calculated to measure the heterogeneity of plant species within the clusters. Whittaker beta diversity (${\beta }_{w}$-diversity) was also performed to define the overall diversity of the plant species in the Sele-Nono forest along ecological gradients. Analysis of Variance (ANOVA) followed by post hoc test was also performed to evaluate the significance of variation in mean values of calculated diversities among the established plant community types using SPSS version 20.0 (Onaindia et al. 2004). Preference ranking on the major threats of the study area was done following Martin (1995) to screen and find out the key human impacts which are aggressively threatening the forest. Other qualitative information gathered through informant interviews was used to complement the quantitative analysis.

## Results

### Floristic composition

A total of 418 vascular plant species representing 108 plant families (Additional file 5: Appendix S5) were compiled in this study. This floristic list indicates that the Sele-Nono forest constitutes about 6% of the flora of Ethiopia and Eritrea. Out of these species, only 335 species were collected from the plots and the remaining 83 were collected from outside the plots. Although the Sele-Nono forest is characterized by a large number of families (108 families), the top fifteen families (Table 2) were the most important ones that contribute about half of the total species composition. Moreover, out of the total species recorded in this study 59 of them (14%) were new records to Illubabur (IL) floristic region in the flora of Ethiopia and Eritrea (Additional file 6: Appendix S6).

**Table 2:** Top fifteen species rich families recorded in Sele-Nono Forest

Family name	Species No.	Percent contribution of families to the total number of species in Sele-Nono forest (%)
Asteraceae	30	7.17
Fabaceae	25	5.98
Acanthaceae	21	5.02
Poaceae	16	3.82
Euphorbiaceae	13	3.11
Lamiaceae	13	3.11
Rubiaceae	13	3.11
Aspleniaceae	12	2.87
Moraceae	10	2.39
Orchidaceae	10	2.39
Celasteraceae	9	2.15
Cyperaceae	9	2.15
Amaranthaceae	7	1.67
Malvaceae	7	1.67
Solanaceae	7	1.67
Total	202	48

**Fig. 6:**
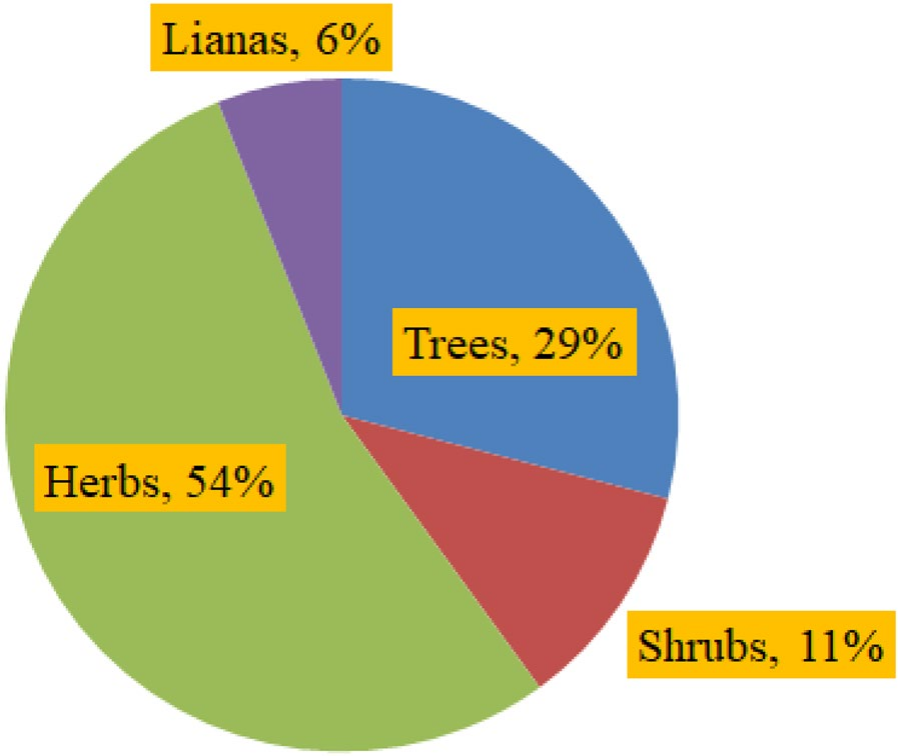
Growth forms of plant species collected in Sele-Nono forest

Among the total plant species documented from the forest, 225 (54%) of them were herbs (including ferns and climbers), 120 (29%) were trees, 26 (6%) were lianas and 47 (11%) were shrubs (Fig. 6). Field observation in the study area also revealed the large abundance of lianas throughout the forest although their species richness accounts only for 6% of the total floristic composition of the area. Of the total plant species compiled in this study, about 25 (6%) of them were endemics to Ethiopia (Kelbessa et al. 1992), and some of these are with some level of IUCN threat category (Vivero et al. 2005, 2006) (Additional file 7: Appendix S7).

### Cluster analysis

**Fig. 7:**
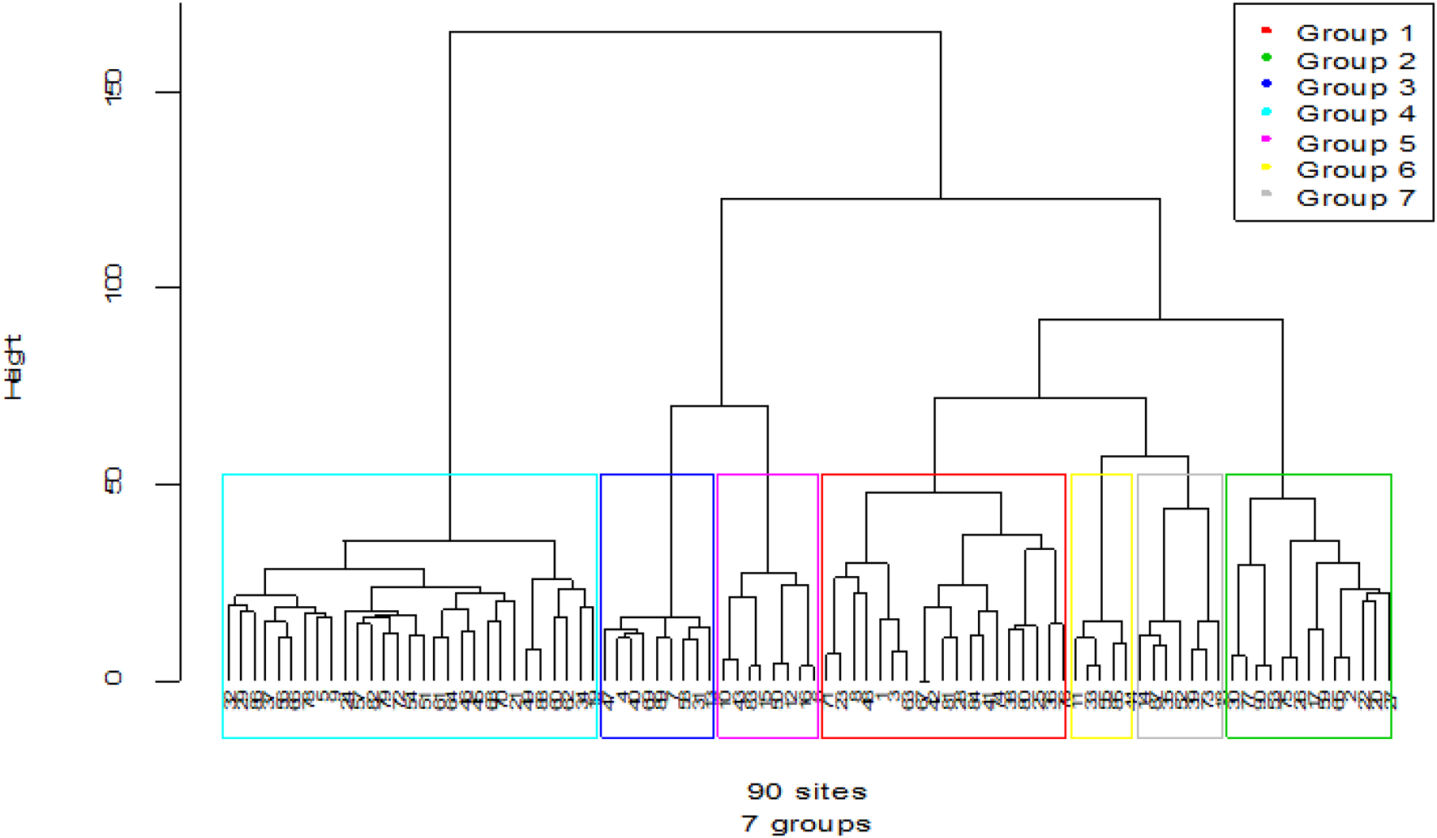
Dendrogram of the vegetation data obtained from hierarchical cluster analysis of Sele-Nono forest using Ward’s method and Euclidean distance (the term ‘Group’ on the legend is synonymous to clusters or plant community types established for the study area, and represented here as C1 to C7 where plots in each community types are presented within the parenthesis as follow: C1(71,23,8,48,1,3,63,67,42,81,28,84,41,74,38,80,25,36,76), C2(30,77,90,53,75,26,17,59,65,22,2,20,27), C3(47,4,40,69,89,70,58,31,13), C4(32,29,86,37,56,66,78,5,9,24,57,82,79,72,54,51,61,64,46,45,68,70,21,49,88,60,62,34,19), C5(10,43,83,15,50,12,16,6), C6 (11,33,55,86,44), C7 (14,87,35,52,39,73,18)

Outlier analysis excluded eight species leaving 327 species for the analysis (Additional file 8: Appendix S8). Using agglomerative clustering we were able to visualize 7 clusters in the dendrogram corresponding to 7 plant community types or “associations” that are expected to be naturally occurring in the study area (Fig. 7). The dendrogram was proved to include more homogenous plots (i.e., plots of high internal similarity with respect to species) in the same cluster (Cophentic correlation coefficient = 0.785, P < 0.001).

### Classification of plant communities in Sele-Nono forest

Seven plant community types were described in the study area and named using two indicators and/or character species identified from synoptic table analysis (Additional file 9: Appendix S9). Indicator species are diagnostic species that are faithful to one community type only; whereas character species are diagnostic species whose mean cover is high in the cluster type under consideration and relatively low in other types (Kent and Coker 1992; Kindt et al. 2011). The use of two indicators and/or character species is the most common approach for naming and describing plant association types (Bekele 1993, 1994; Woldu 1999; Soromessa et al. 2004). The result of synoptic value shows that some of the species are indicator species as they are only faithful to one of the plant community types under consideration and not found in other types. However, many other species occur over wide altitudinal ranges or community types showing a continuous variation of vegetation; but are most dominant in terms of their mean cover within only one of the cluster types.

Accordingly, community one is named as *Olea welwitschii*—*Elaeodendron buchananii* community type, and mainly includes the vegetation within the altitude of 1600–1900 m asl in Sele-Nono forest. This community type is often located on steep topographies and spatially different localities such as MOLAYE, YEBECHA, WANGES (all in Onose Kebele), GAGO (in Gemechesa Kebele), QETO (in Bontu-Korma Kebele); KIMO and NONO-BERBERSA Kebele forests; and river lines of CHETA, GURACHA, and SEJA. Representative woody species in this community includes *Acanthus eminens, Bersama abyssinica, Clausena anisata, Cordia africana, Dracaena steudneri, Embelia schimperi, Erythrococca trichogyne, Hallea rubrostipulata, Isoglossa somalensis, Lobelia giberroa, Maesa lanceolata, Macaranga capensis, Maytenus gracilipes, Millettia ferruginea, Pittosporum viridiflorum, Rytignia neglecta, Sapium ellipticum, Teclea noblis, Vepris dainellii*. Herbaceous species in this community includes *Achyranthes aspera, Aframomum corrorima, Desmodium repandum, Hypoestes triflora, Hypoestes forskaolii, Panicum spp, Paullina pinnata, Piper capensis, Tiliacora troupinii, Salvia nilotica*.

Community two is *Alstonia boonei– Manilkara butugi* community type, and often found on the extreme low land of the study area between an altitude ranges of 840–1250 m asl. This community type is often located on gentle terrains of WAKA, ARBE, KOPI, YAKEMA, GEMECHESA WELKKETESA Kebele forests on their lowland extremes. Common woody membership species in this community type includes *Acalypha ornate, Anthocleista schwinfurthii, Allophylus macrobotrys, Alstonia boonei, Argomuellera macrophylla, Baphia abyssinica, Celtis zenkeri, Dracaena fragrans, Ficus sycomorus, Garcinia buchananii, Garcinia ovalifolia, Lecaniodiscus fraxinifolius, Manilkara butugi, Milicia excels, Pouteria alnifolia, Pouteria altissima, Saba comorensis, Whitfieldia elongate*. Some of the herbaceous species common in this plant community types include *Ageratum conyzoides, Achyrospermum schimperi, Phaulopsis imbricata, Panicum atrosanguineum, Isodon schimperi, Plantago palmate, Guizotia scabra, impatiens hochstetteri, Stictocardia beraviensis*. Field observation by the authors reveal that this community is disturbed prevalently by the conversion of forest land to cropland (Maize, Enset ventricosum, Colocasea esculenta) through anthropogenic fire. Moreover, it was evident that these areas were heavily disturbed by anthropogenic activities such as charcoaling, logging, the gathering of Dioscorea roots, and the likes. Oral communication with elder people, who were born and live in the area, indicates that the size of the forest area becomes highly diminished than before.

Community three is *Arundinaria alpina– Oplismenus hirtellus* community type and is found in the uppermost altitudes of the study sites within altitudes of 2300-2448 m asl. It covers the uplands of TUPI and KOMBOLCHA Kebele forest in the study area. Though it is mainly dominated by one species, *Arundinaria alpina*, it still contains some dominant species, which include *Allophylus abyssinicus, Bersama abyssinica, Dracaena afromontana, Galiniera saxifrage, Polyscias fulva, Prunus africana, Schefflera myriantha, Schefflera volkensii, Syzygium guineense subsp. afromontanum,*. Herbaceous species include *Amauropelta bergiana, Athyrium schimperi, Ajuga integrifolia, Blotiella glabra, Cyathula polycephala, Thunbergia alata, Veronica abyssinica.*

Community four is *Pouteria adolfi-friederici–Dracaena afromontana* community type, and mainly observed between altitude ranges of 1850–2300 m asl in the study area. It covers most parts of the study area such as the forests of DECHA, SOCHOSO, SELASE, DERBETA, and the highlands of WAKA, HARO, KOPI, YAKEMA, BONTU KORMA and GOROSO forest. Common species in this community includes *Acanthopale ethio-germanica, Asystasia gangetica, Barleria ventricosa, Albizia gummifera, Allophylus abyssinicus, Brillantaisia madagascariensis, Clausena anisata, Combretum paniculatum, Cyathea manniana, Dalbergia lacteal, Deinbollia kilimandischarica, Embelia schimperi, Galiniera saxifraga, Hippocratea africana, Ilex mitis, Lepidotrichilia volkensii, Maytenus spp., Olea capensis, Oxyanthus speciosus, Pittosporum viridiflorum, Plectranthus garckeanus, Pouteria adolfi-friederici, Rothmannia urcelliformis, Rytignia neglecta, Ruellia prostrate, Teclea nobilis, Vepris dainellii, Schefflera abyssinica, Syzygium guineense*. Common herbaceous species included in this community types include *Asplenium sandersonii, Arthropteris monocarpa, Celosia argentea, Crassocephalum macropappurm, Desmodium repandum, Dissotis senegambiensis, Elatostemma monticolum, Habenaria holubii, Impatiens ethiopica, Lycopodium c1avatum, Oplismenus hirtellus, Scadox nutans*, *Thalictrum rhynchocarpum, Tristemma mauritianum,* and many others. Interview with District leaders and key informants reveal that these highland forests have been called Kobo forests for honey production by the local people. The Kobo system is a traditional forest management practice in the study area that gives the local people a sense of ownership for a certain block of forests demarcated by big trees and/or other physical features like rivers and small streams and exclusively used for hanging beehives and hunting to sustain their livelihoods.

Community five is *Cyperus longibracteatus- Cyperus dereilema* community type, and it refers to the vegetation predominantly found on fragile wetlands and other pocket swamp bodies. These are permanently or seasonally flooded areas dominated by grasses and/or sedges between 1640 and 1850 m asl. This community is mainly found in the wetlands (and/or grassland during the dry season) of GOROSO, KIMO, QOTI, ASENDABO, TUPI, GEMECHESA, WELKETESA, and the like. Although the primary vegetation in this community is tall grasses, sedges and ferns, scattered woody species are also notable. The major vascular plant species that characterize the wetland habitat include *Cyperus dereilema, Floscopa glomerata, Panicum calvum, Pennisetum macroururn, Pennisetum trachyphyllum, Persicaria setosula, Rumex nepalensis, Snowdenia polystachya, Sporobolus pyramidalis*. The grasses grown in this community type are a common source of livestock feed and thatches, and field observation also proved free grazing on these habitats particularly during the dry season as a threat to this community type.

Community six is *Coffee arabica- Trichilia dregeana* community type, and mainly found within the altitude range of 1600–1800 m asl. It is dominantly observed in ASENDABO, KIMO, DERBA, GEMECHISA, WELKETESA, YAKEMA, KOPI, and the likes. The main plants included in this community are *Apodytes dimidiate, Bridelia micranta, Brucea antidysnterica, Cassipourea malosana, Coffea arabica, Croton macrostachyus, Dracaena steudneri, Ehretia cymosa, Erythrococca trichogyne, Euphorbia ampliphylla, Ficus exasperate, Ficus thonningii, Ficus vasta, Ficus ovata, Ficus sur, Hippocratea goetzei, Hippocratea africana, Nuxia congesta, Oncinotis tenuiloba, Paullinia pinnata, Psidium guajava, Polysicas fulva, Ritchiea albersii, Sapium ellepticum, Senna petersiana, Tiliacora troupinii, Trema orientalis, Trichilia dregeana*. Field observation showed that this community type is more vulnerable due to forest management (thinning the understory shrubs, logging canopy trees, etc.) for increasing the production of coffee beans for the purpose of household consumption. Moreover, informal interview with local elders showed that most local people prefer this area for the expansion of smallholder coffee agriculture as these areas are average between the highland (where there are not many natural coffee plants) and lowlands (where there is much coffee but unconducive area for a living). As a result, many of them have coffee plots in the forests where they collect coffee beans for selling to subsidize their livelihood. On top of this, expansion of settlements and recent development in road construction in this community type is a major threat to the diversity and richness of plants. As a result, the physiognomy of vegetation in this community is not characterized by continuous cover spacing; rather plants are widely spaced out.

Plant community seven is *Trilepisium madagascariense*—*Morus mesozygia* community type and is found at altitudes between 1200 and 1500 m, asl along with the courses of Baro, Genji, and Guracha rivers. Common woody species in this community types includes *Acalypha ornate, Albizia grandibractiata, Alblzia schimperiana, Anthocleista schweinfurthii, Argomuellera macrophylla, Baphia abyssinica, Celtis philippensis, Celtis gomphophylla, Cissampelos mucronata, Dioscorea praehensilis, Diospyros abyssinica, Dracaena fragrans, Ficus exasperata, Ficus sycomorus, Gouania longispicta, Justicia bizuneshiae, Justicia ladanoides, Mimosopes kummel, Morus mesozygia, Phoenix reclinata, Strychnos mitis, Trichilia dregeana, Trilepisium madagascariense, Turraea holstii.* Common herbaceous species includes *Ceropegia cufodontis, Eulophia guineensis, Marantochloa leucantha, Oryra latifolia, Setaria megaphylla.*

### Diversity analysis

Diversity analysis in vegetation ecology is an important tool to measure or infer the effect of human activity (disturbance) on plant diversity. Accordingly, diversity analysis (species diversity, richness, and evenness) for each cluster was calculated using R as shown in Table 3. Moreover, Whittaker’s alpha (α) and beta (${\beta }_{w}$) diversity were also calculated for each cluster (Whittaker 1972; Magurran 1988) so as to measure the degree of heterogeneity of species composition. Alpha diversity (α-diversity) was calculated as the average species richness per plot (i.e., species density). Beta (${\beta }_{w}$) diversity was calculated on the basis of the ratio of the total number of species to the average number of species.

**Table 3:** Diversity analysis for each of the seven plant community types in Sele-Nono Forest

Cluster number	Altitude Range(m, asl)	No. of plots	Species richness (S)	Shannon diversity index		Whittaker’s diversity	
				Species diversity (H)	Species evenness (J)	α-diversity	${\beta }_{w}$-diversity
1	1600–1900	19	101	4.09	0.88	24.11	4.18
2	840–1250	13	71	3.82	0.89	19.69	3.60
3	2300–2448	9	58	3.62	0.89	14.00	4.14
4	1850–2300	29	186	4.36	0.83	29.20	6.36
5	1640–1850	8	55	3.64	0.90	16.25	3.38
6	1600–1800	5	46	3.53	0.92	24.40	1.88
7	1200–1500	7	91	4.16	0.92	30.66	2.96

Based on the result of the data (Table 3), it can be said that cluster 4 is a cluster with high Shannon diversity (H = 4.36) of plant species and is found on the uppermost altitudes of the study area before reaching the bamboo zone. The higher the values of Shannon evenness (J), the more even the plant species are in their distribution within the cluster. Thus, clusters 5, 6 and 7 have a more even distribution of individuals among various species in each cluster (J > 0.90) than the other clusters.

As far as species richness and beta diversity is concerned it can be noted from the above table (Table 3) that they are strongly and positively correlated with the number of plots in the communities (Pearson correlation coefficient, r = 0.92). Similarly, there was positive correlation between plot numbers and species diversity (H) (r = 0.75), and strong negative correlation with eveness (J) (r = − 0.95).

On the basis of species richness, cluster 4 (*Pouteria adolfi-friederici– Dracaena afromontana* community type) and cluster 1 (*Elaeodendron buchananii- Olea welwitschii* community type) contain a large number of species than the other clusters. Actual field observation during the study period revealed that community type 6 is a major coffee growing area and hence most local people prefer it for settlements so as to subsidize their livings; whereas community 5 (pocket wetlands/flooded grasslands) is mainly serving as grazing land for animals particularly during the dry season. Community 3 (the bamboo zone) is the peak and wettest place in the study area.

From the above Whittaker beta diversity analysis (Table 3), it can be seen that the degree of variation of vegetation turnover along the plots in each cluster is not so high (${\beta }_{w}$<5) except for cluster 4 (${\beta }_{w}$>5); whereas too low beta diversity was obtained for cluster 6, which is the species-poor plant community type. Whittaker’s beta diversity (${\beta }_{w}$-diversity) index was also calculated in R to assess the overall heterogeneity of plant species in the study area. The result indicates that there is high beta diversity (${\beta }_{w}$ = 12.04) of plant species across the community types in the Sele-Nono forest, which leads to the conclusion that each plant community type has a heterogeneous species composition.

### Variation on the relative diversity of plants in Sele-Nono forest

**Fig. 8:**
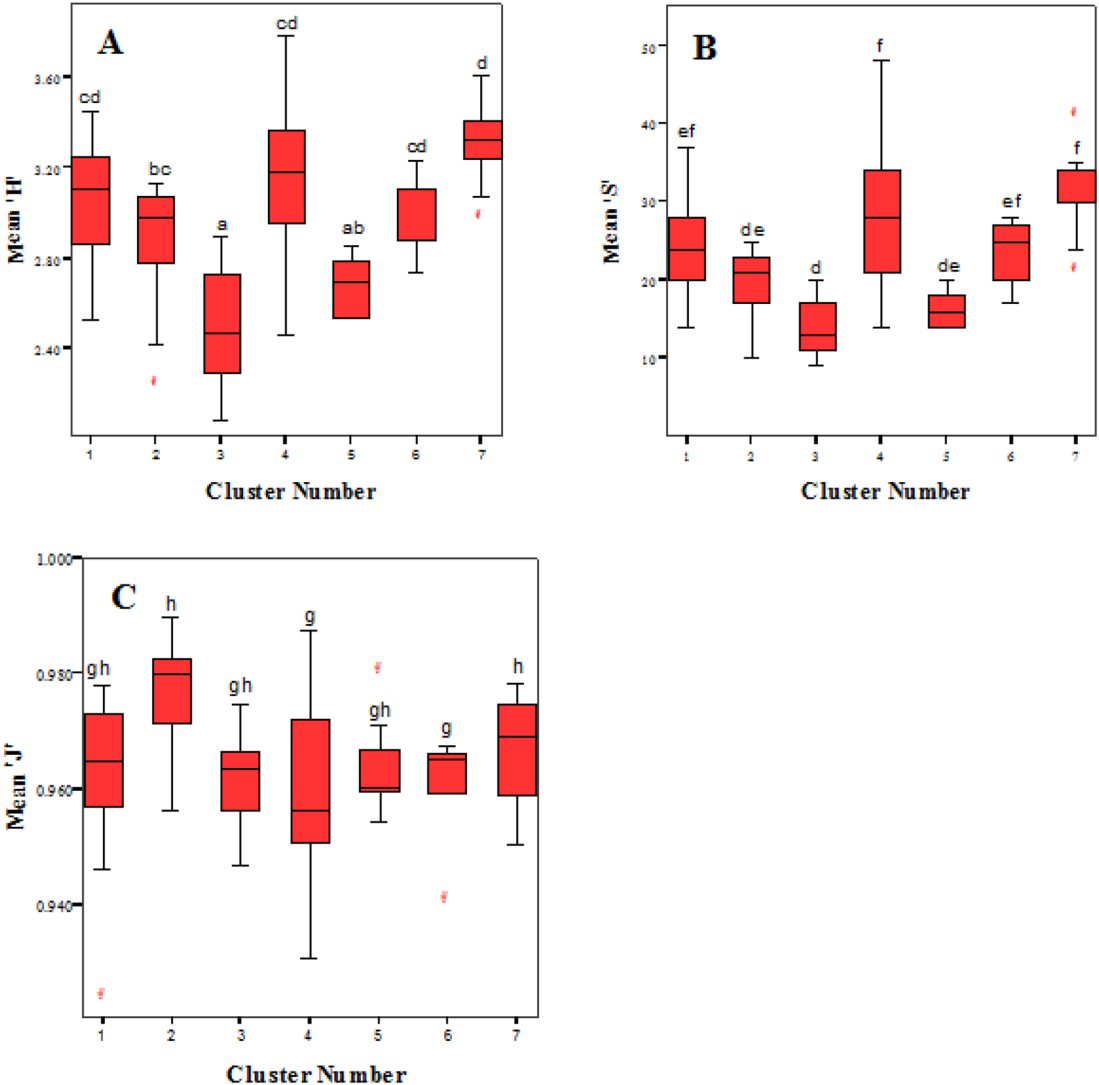
Mean values of plant diversity per plot for the different clusters in the study area. **A** Variation of mean species diversity. **B** Variation of mean species richness. **C** Variation of mean species evenness between the clusters. Boxes represented by the same letters are not significantly different at P < 0.05

Initial analysis of variance (ANOVA) using “aov” approach in R showed that there is a significant difference in diversity such as in species diversity (F = 10.89, P < 0.05), richness (F = 9.83, P < 0.05), and evenness (F = 3.46, P < 0.05) between the plant community types (Fig. 8). ANOVA was performed after checking the assumptions of linearity using QQ plot and Shapiro–Wilk normality test (P < 0.05) and homogeneity of variances using Levene statistic (P > 0.05).

From the above figures (Fig. 8), it can be revealed that mean species diversity was significantly higher at clusters 1, 4 and 7; and lower at cluster 3 (the community with the alpine bamboo thicket) and cluster 5 (the community with a high level of grazing intensity). Similarly, a significantly large number of species per plot were found in cluster 4 and 7; and cluster 3and 5 has significantly less number of species. Regarding species evenness, significantly high species evenness per plot was revealed at cluster 2 and while significantly low evenness was observed at cluster 4.

### Influence of environmental factors on the variation in species composition of plants

The result in this study shows that there is a significant correlation between the species composition and environmental variables (Mantel statistic: r = 0.64, P = 0.001). from the environmental data collected for this study (Additional file 10: Appendix S10), a forward selection procedure (Monte Carlo permutation tests, n = 999) screened out the following environmental variables (Table 4) to be more responsible (P < 0.05) for the distribution of plants and structuring their community composition in the study area. These environmental variables were able to explain 12.5% of the variability in the floristic data as can be understood from the inertia or variance represented by Chisq (Table 4).

**Table 4:** Most influential environmental factors that affect the distribution of plants in Sele-Nono Forest

Environmental factors	Df	Chisq	F	N.Perm	Pr(> F)
Altitude	1	0.4002	3.4289	999	0.001^***^
Disturbance	1	0.3736	3.2007	999	0.001^***^
Slope	1	0.2502	2.1434	999	0.001^***^
Organic matter (OM)	1	0.2177	1.8648	999	0.001^**^
Nitrogen (N)	1	0.1679	1.4388	999	0.013^*^
Residual	84	9.8049			

Signif. codes: 0 ‘***’ 0.001 ‘**’ 0.01 ‘*’ 0.05

**Fig. 9:**
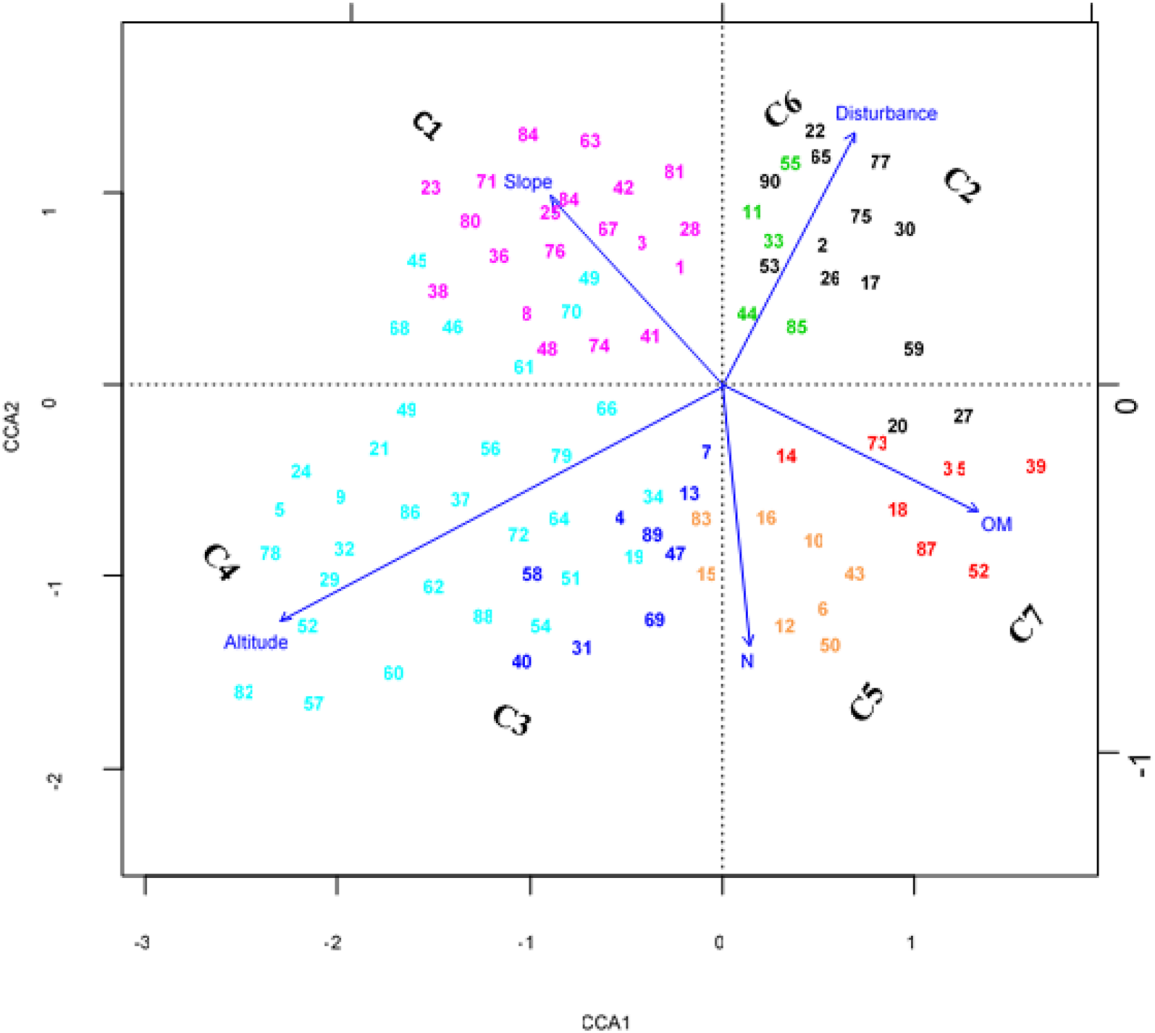
CCA showing the relationship of the environmental factors among themselves and on the distribution of plants in the study area (Numbers inside the ordination diagrams represents plot numbers; and C denotes community number; arrows representing environmental variables; and arrow length shows the strength of the environmental variable)

The correlation of these environmental factors with themselves and their influence on the distribution of vegetation in the study area was projected as follows using the Canonical Correspondence Analysis (CCA) technique (Fig. 9).

The first and second ordination axis of the CCA explained 35.51% and 26.08% of the constrained variation respectively, leading to capture and explain a cumulative of 61.59% of the constained inertia. From the above ordination diagram (Fig. 9) it can be seen that altitude was strongly associated with species composition as indicated by the long arrow in the ordination diagram indicating that it is the main environmental factor responsible for the spatial variation of vegetation in the study area. The plant communities that are distributed in the higher altitude of the study area (C1, C3 andC4), which all together form part of Afromontane forest were ordinated to the left side of the diagram; whereas the clusters that are distributed to the lower altitude (C2 and C7), which altogether forms part of the transitional rainforest were ordinated at the positive end of axis 1. A negative correlation between altitude and disturbance was also evident from the ordination diagram. Vegetation of the swampy areas (C5) has a direct relationship with the nitrogen content of the soil, whereas *Morus mesozygia—Trilepisium madagascariense* community type (C7) is mainly constrained by the high organic matter content of the soil in the area.

### Threat to vegetation of Sele-Nono forest

The semi-structured interview conducted with the informants reveals that the forest vegetation of the study area is depleting from time to time basically due to deforestation and forest degradation. The following are common reasons summarized through the interview,

- Expansion of Agricultural lands (conversion of forest land use to farming land)
- Expansion of settlements/urban boundaries due to population growth
- Fire (causing a wide distraction of vegetation and affecting the regeneration. It is often set deliberately either for farm clearing, or driving out bees during honey collection from the traditional beehives or may get out of control. Sometimes by smokers leaving butts of cigarettes unextinguished in the forest)
- Over-grazing particularly for herbaceous plants (because it hinders regenerations, decreases species diversity, and if it is heavily grazed it paves ways for erosion by wind, water etc.)
- Clearing of forests for wide and far-reaching road constructions
- Thinning of understorey shrubs other than Coffea for improving coffee bean productivity (i. e., conversion of “forest-coffee systems” into “semi-forest coffee systems” as defined by Woldemariam 2003)
- Selective removal of trees and/shrubs for construction purposes, timbering, charcoal production, harvesting of fuelwood, etc. Some of the species frequently preferred for logging by the local people include *Pouteria adolfifriederici, Apodytes dimidiate, Celtis africana, Cordia africana, Croton macrostachyus, Ekebergia capensis, Prunus africana, Olea welwitschii, Polyscias fulva and Syzygium guineense*. These logging practices have been reported to exist in the study forest since the time that dates back beyond the knowledge of the local people. The current participatory forest management (PFM) approach practicing in the study area was reported by clan leaders at least in limiting the uncontrolled logging.

**Fig. 10:**
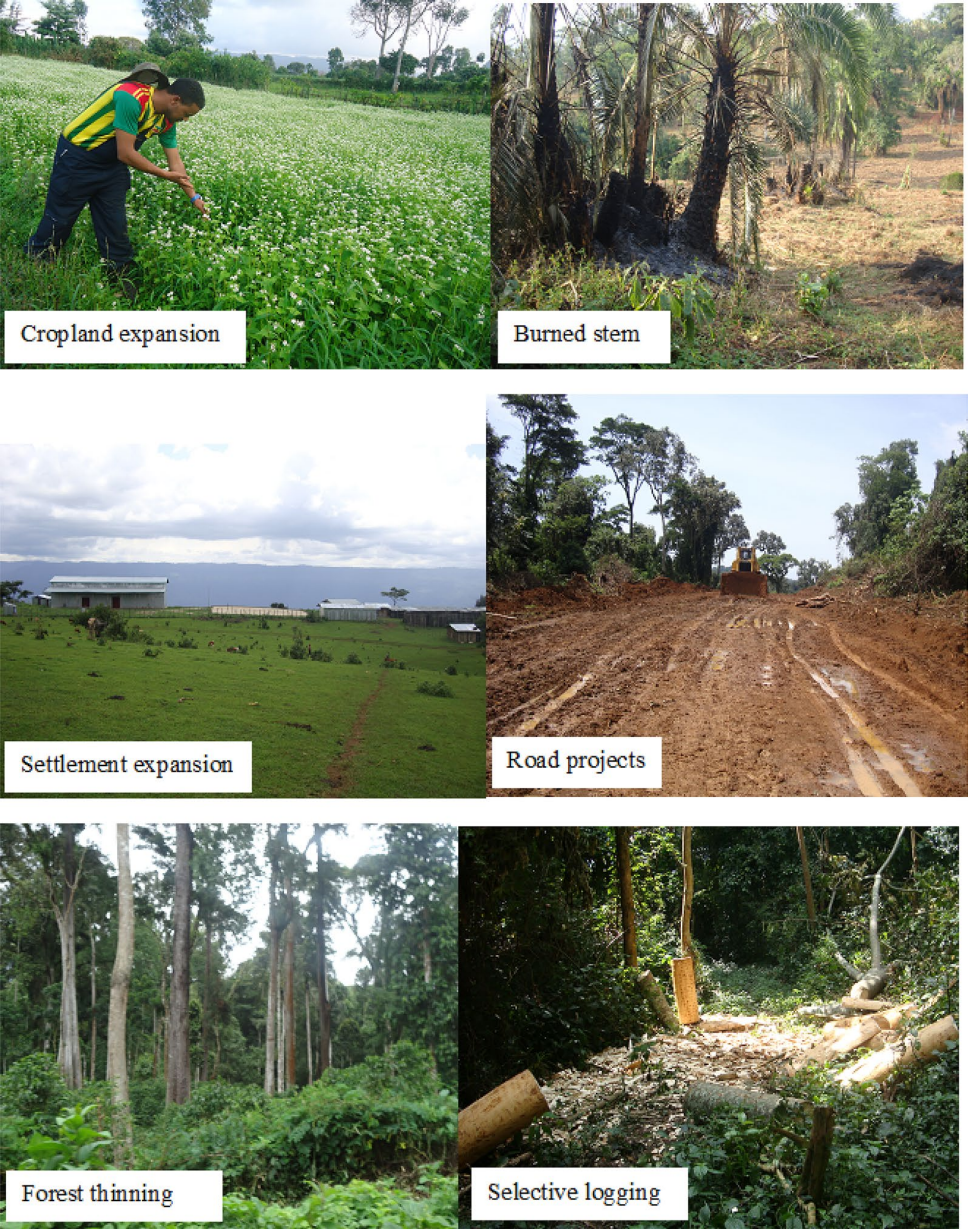
Common threat to the vegetation of Sele-Nono Forest

Moreover, actual field observation (Fig. 10) supported the interview result.

To indicate the above listing of environmental degradation in order of their impact, preference ranking was applied by taking ten key informants following Martin (1995) (Table 5).

**Table 5:** Results of preference ranking values from ten respondents (A-J) on priority ranking of five threats to the vegetation of Sele-Nono forest (based on their degree of causing damage: 1 = least damaging, 7 = most damaging)

Lists of major threats to Sele-Nono forest	Key informants (coded A-J)											Rank
	A	B	C	D	E	F	G	H	I	J	Total	
Agricultural land expansion	7	6	7	7	7	5	7	7	6	7	66	1st
Selective removals Forest thinning	6 5	7 5	5 6	3 5	6 4	6 7	6 5	6 4	7 5	5 6	57 52	2nd 3rd
Settlements boundry expansion	4	4	4	6	5	3	4	5	3	4	42	4th
Road development	3	1	2	4	1	2	3	3	4	1	24	5th
Over-grazing	2	3	1	2	3	4	1	2	1	3	22	6th
Fire	1	2	3	1	2	1	2	1	2	2	17	7th

From the above table (Table 5) it can be seen that the study forest is primarily threatened by forest clearing for agriculture purposes and forest degradation through selective removals of woody species and forest thinning practices for optimizing coffee bean production. Moreover, settlement expansion or population increment and road projects are also notable threats to the Sele-Nono forest. Overgrazing and fire were found to be the least serious threat to the plant biodiversity of the Sele-Nono forest.

## Discussion

### Floristic composition of Sele-Nono Forest

It is frequently claimed that understanding floristic composition is the primary phase for the management of forest ecosystems (Kneeshaw et al. 2000; Simberloff 2001; Sanjit and Bhatt 2005; Ssegawa and Nkuutu 2006; Negesse and Woldearegay 2022). In agreement with this sentiment, this study showed that the Sele-Nono forest sheltered a large number of vascular plants (418 species) falling into diverse plant families (108 families) and considerable growth forms (herbs, shrubs, lianas, and trees). The finding of this study also demonstrates that the Sele-Nono forest contains species for moist montane on the highlands plant community types (C1 and C4) and transitional rainforest characteristic species (Friis et al. 2011) on the lowland plant community types (C2 and C7) suggesting that Sele-Nono forest belongs to moist montane and transitional rain forest, which had not been included on the vegetation map of Ethiopia developed by Friis (1992).

The finding of this study also revealed that the Sele-Nono forest contains more species than the species reported for Bonga forest (220 species) by Schmitt et al. (2010), Gura Ferda forest (196 species) by Denu (2006), Masha forest (130 species) by Assefa et al. (2013), Yayu forest (220 species) by Woldemariam et al. (2002), Sheko forest (374 species) by Senbeta et al. (2007), Gesha and Sayilem forest (300 species) by Addi et al. (2020), Gerba Dima forest (180 species) by Dibaba et al. (2022). This indicates that the current study area is one of the richest forest ecosystems in the southwest forest of Ethiopia. This may be attributed to its largness in size (about 150,000 ha forest area) that goes with the ecological concept of “largness and singularity” in the theory of island biogeography which focuses on the overarching importance of single but large natural reserves or habitates to harbor more number of species than if it would have been divided into several small natural habitates with the same total area (Diamond 1975; Whittaker and Fernandez—Palacios 2007; Triantis and Bhagwat 2011; Wilson and MacArthur 2016). The large number of species records in Sele-Nono may also be attributed to prsecence of relatively less anthropogenic pressure to the forest due to low population number (CSA 2007) and inaccessible roads.

The richest plant families reported in the current study area are Asteraceae, Fabaceae, Acanthaceae, Euphorbiaceae, Rubiaceae, and the like. Most of these families are also among the top families in the flora of Ethiopia and Eritrea (Kelbessa et al. 1992) in general and in the different vegetation patches of the moist southwest forests in particular (Yeshitela and Bekele 2002, Woldemariam 2003, Yeshitela and Bekele 2003; Senbeta et al. 2005; Kelbessa and Soromessa 2008; Addi et al. 2020; Dibaba et al. 2022). The prevalence of the family Asteraceae in the study area indicate that the forest might have been under a certain level of disturbance as plants of the Asteraceae family are often ruderal (early colonizers); and normally prefer open and disturbed lands to grow (Malcolm 1994; Turner 1996; Tadesse 2004; Awoke et al. 2022).

This study also reports the presence of a large number of trees and shrubs next to herbs. This may suggest that there was less selective utilization of tree species as opposed to some other forest, for instance, the Bishan Gari/Kimphee natural reserve in Southern Ethiopia (Senbeta and Teketay 2003). Moreover, the higher floristic composition of the forest floor may be a consequence of low grazing intensity in the forest (Onaindia et al. 2004). The occurrence of a large proportion of herbs is a common phenomenon in other moist forests of Ethiopia (Addi et al. 2016; Senbeta et al. 2007). Moreover, the relatively high number of liana species in the forest may indicate that there have been encroachments followed by continuous disturbances in the area (Putz 1984; Villagra et al. 2013). The presence of high numbers of lianas has also been noted elsewhere in the moist southwest forests of Ethiopia (Senbeta et al. 2005) and elsewhere in moist forests of Africa (Girma 2011; Addo-Fordjour et al. 2013; Uwalaka et al. 2021).

The finding in this study also reported that about 6% of the total plant species compiled in this study are endemics to Ethiopia (Additional file 2: Appendix S2). The restriction of these taxa is probably a consequence of limited dispersal ability. The low endemicity of plants is a common phenomenon for the vegetation of the moist SW forest of Ethiopia in particular (Friis 2009) and for the vegetation of the country at large (Kelbessa et al.^1992^).

### Community types and diversity of plants in Sele-Nono forest

For the purpose of plant biodiversity assessments of a forest ecosystem, ecologists often recommend studying it at a certain finest floristic unit so as to draw a better picture of the richness and diversity of plant species of an area (Mueller-Dombois and Ellenberg 1974; Kent and Coker 1992). In line with this sentiment, this study established and described seven plant community types. None of these community types were poor with respect to vascular plant diversity (H > 1.5). According to Kent and Coker (1992), the optimal value of the Shannon diversity Index (H) lies between 1.5 and 3.5. This diversity of vascular plant species is comparable with other moist forests of Ethiopia (Nigatu and Tadesse 1989; Lulekal et al. 2008; Gurmessa et al. 2013; Kebede et al. 2013).

Among the community types described in this study community types 1 and 4, which are mainly represented by the moist evergreen Afromontane forest (MAF) parts of the study area, had relatively high species diversity and richness of plants. The high diversity and richness of plants in the moist evergreen Afromontane forest of Ethiopia were also reported by Friis (2011). One possible reason for having a high richness and diversity of plants for these community types in the current study area could be a consequence of the large sample size. Another possibility may be the altitude gradient since these community types were found at reasonably high altitudes that provide optimal environmental conditions required for their physiological needs and favor vegetation growth (Rosenzweig 1995; Schmitt et al. 2013). Moreover, the tradition of the local people named as KOBO system might have contributed for the higher richness and diversity of plants in these plant community types as this culture emphasizes on the use of the forest for traditional apicultural practices and other non-timber forest products. The same observations was made in the previous study on the area (Tamene 2010) and elsewhere in the southwest forest (Wood et al. 2019). This communal culture has also been reported elsewhere to have conservation value in other southwest forests of Ethiopia (Tadesse and Woldemariam 2007; Woldemariam and Getaneh 2011). On the contrary, lower richness and diversity of plants were found in the second community type (C2) which was found in the lower altitude of the study area (840–1200 m, asl). Possible reason could be high level of disturbance and effect of local climatic variation. Moreover, geographical isolation might also have contributed to the low diversity of plants in this forest since this community type is bordered by large tracts of dry and lowland vegetation of Gambella Regional state (Friis 1992).

The findings of this study revealed that community types 3, 5, and 6 are with relatively low species richness and diversity of plants. The low diversity and richness of species in community type 3, which was mainly represented on the peak of mount TUPI (in Tupi Kebele) of the study area, might be a consequence of the chilly climatic nature that may be too harsh to permit other plants to grow well except few species such as the alpine bamboo. Moreover, the low richness and diversity of plants in this community type may be attributed to its small size. Inline with this Rahbek (1997) reported that areas on top of conical mountains are not only cooler but they are also smaller; and are often poor in plant species. The low richness and diversity of species in community type 5 may be associated with the absence of a number of woody plants. This absence might be due to the fact that the stagnant water in the swampy places restricts the root growth of woody plants due to poor soil aeration. Lower diversity of the existing species in this community type could also be attributed to the anthropogenic influences such as over-harvesting of grasses and sedges important for beautifying floors in the culture of the local people mainly for holidays, and for making roofs of the huts in the rural areas. Moreover, this plant community type is the sole place for grazing; and consequently, these places are over-grazed by livestock. Similar observations on the wetlands of southwest forests of Ethiopia were noted in previous studies (Woldu 1999; Hailu 2003; Woldu and Yeshitela 2003; Tolossa et al. 2022). The low diversity and richness of plant species in community type 6 might also be attributed to their occurrence close to settlements that were mainly situated at 1600–1800 m asl. Studies elsewhere also suggested that areas close to settlements are often prone to poor diversity of plants (Fahrig 2003; Somashekar et al. 2003; Specht et al. 2015). The preference of the local people to settle at this altitude zone (or community type) is perhaps a consequence of the occurrence of rich coffee plant resources, which is an important cash crop in the study area (Ango et al. 2020). According to the report in Wikipedia Free Encyclopedia (2015), the use of Coffee beans as a cash crop in the area is believed to start during the time of Dejazmach Ganame, governor of Illubabur during the time of Minilik II (1889–1913). The dominancy of wild coffee plants in this altitude zone may be attributed to its dispersal limitation and species selection. Given this community type is more abundant in wild coffee (*Coffea arabica*), which is a flagship species at the international level, it would help ecologists to consider this matter under any possible conservation activities. This altitude zone is also reported to be a dominant habitat for naturally growing coffee (*C. arabica*) population in other Afromontane rainforests of Ethiopia (Teketay 1999; Woldemariam et al. 2002; Senbeta and Denich 2006; Schmitt et al. 2009; Geeraert et al. 2019; Hwang et al. 2020).

In this study we also noted a positive and strong correlation between sample plots and species number of the identified communities and consequently with species diversity and beta diversity. Similar observation were noted from the findings of similar works elsewhere in the country (Tadesse et al. 2017; Awoke et al. 2022; Sewale and Mammo 2022). This is perhabs the larger the plot numbers along a specific gradient the higher will be the probability of having diverse ecological niche that will be responsible for more number of species to exist (Peterson et al. 2011).

Based on the beta diversity analysis the study area can be regarded as a heterogenous forest (${\beta }_{w}$ = 12.04) at landscape level (Woldu 2017). This could be due to the spatial and environmental heterogeneity of the area. At plant community level cluster 4 is the more heterogeneous (${\beta }_{w}$ > 5) forest part indicating a high turnover of plant species among the sample plots. This can be further illustrated in the box plot that shows high variability of data set between the samples. The reason for high beta diversity or variability between the samples could be the prescence of large sample size in this community type and relatively large elevation gradient. On the other hand, community 6 and7 scored the lowest beta diversity indicating each of these community types are relatively homogenous, and characterized by high compositional similarity in the sampled plots as it can also be noted from high Shannon evenness value (J = 0.92). This is still associated with the fewer number of sample plots found in the community types. Similar annotations were indicated by Yastrebov (1996) and Jurasinski et al. (2009).

### Influence of environmental factors on the variation in species composition of plants in Sele-Nono forest

It is known that the vegetation cover of a given area is developed due to its long-term interaction with biotic and abiotic factors. Consequently, the finding of this study showed that the existing composition and diversity of plant species in the Sele-Nono forest is partially (12.5%) judged by environmental factors such as terrain variable (altitude and slopes), edaphic variables such as total nitrogen (N) and organic matter (OM) and disturbance factors.

The findings of this study show that altitude is a substantial factor for the high richness and diversity of plants on highland forests, particularly in community type 4 of the study area; and for the low richness and diversity of plants in community type 3. This may be attributed to the influence of altitude to build favorable microclimates (temperature and moisture) at the altitude range of 1850–2300 m asl for optimal growth of plants; and hostile microclimate on the extreme altitude (2300–2448 m asl) to limit the growth of plants in the study area due to the induced change to the soil environment (soil chemistry, nutrient release, organic matter decomposition). A similar explanation was given by Schmitt et al. (2013) about vegetation variation along an altitude gradient in moist montane forests of Ethiopia and elsewhere (Kabrick et al. 2008; Fonge et al. 2013).In this regard, altitude is often claimed as an important factor that has a strong influence on the vegetation diversity of moist southwest forest of Ethiopia in particular (Woldu et al. 1989; Nune 2008; Schmitt et al. 2010; Friis et al. 2011) and most montane ecosystem of the world in general (Lovett 1993; Eilu et al. 2004; Cirimwami et al. 2019; Asefa et al. 2020).

The finding of this study also shows that slope is an important environmental factor for community type 1 (C1) to harbor a large number of plant species. This may be attributed to the improperness of these areas to access it for use such as for plowing and crop cultivation, which are often regarded as serious threats to forests (Woldemariam 2003; Tadele et al. 2014; Asefa et al. 2020).

The study also showed that a high level of disturbance was one of the chief environmental factors near the lower limit of the study area. Clearing of forests and their conversion into croplands, and selective removal of trees were the main disturbance factors in C2 and C6 respectively. These factors mainly lead to the decline in species richness and diversity of plants in these community types. This could be because the clearing of forests and conversion into croplands in the study area is expected to reduce litter production, increase soil erosion rates and decomposition of organic matters (OMs) by oxidation (Lawton et al. 2001; Fahrig 2003); whereas disturbance through selective exploitation of canopies creates openings in the forest that may alter the local microclimate which would dry the soil rapidly and cause loss of nutrients through run-off (Berry et al. 2008; Ruger et al. 2008; Jiren et al. 2020). In both cases, disturbance reduces the natural stability of the study area which subsequently might impede the recovery of trees and shrubs. Results of many other studies in Ethiopia (MOA 1990) and elsewhere (Lovett 1999; Aubert et al. 2003; Onaindia et al. 2004; Rad et al. 2018; Chapagain et al. 2021) also shows similar findings in that anthropogenic disturbance leads to low species richness.

This study has also assessed the influence of edaphic factors on the diversity of plants. The influence of soil factors on floristic composition was also noted elsewhere (Infante-Mata et al. 2011). Accordingly, this study revealed that soil organic matter (OM), and total nitrogen contents (N) were the most significant edaphic factors in the Sele-Nono forest for the distribution of plants. Among the plant community types described in the lower altitude of the study area, (< 1500 m asl) community type 7 is with a high diversity of species. This might be related to the high organic matters (OM)content of the area, which could give the soil a buffering capacity by resupplying the soil solution when important nutrients are removed by uptake or leaching (Cole 1995; McCauley et al. 2017) and hence contribute for the rapid growth of plants. The high temperature that favors the decomposition of forest litter might have resulted in to have high-level of soil organic matter (OM) in this community type (community type 7). The presence of high temperature in this altitude zone of the moist southwest forest was also evident by Friis et al. (2011). In relation to these assumptions, vegetation ecologists have claimed that, unlike dry forests where moisture is the most decisive factor for decomposition and turnover rates, so does is the temperature in moist forests. On the contrary total nitrogen content of the soil is a more responsible edaphic factor for the vegetation pattern in community type 3, which is mainly represented by the vegetation of swampy lands. This may be partly because the wetlands/swampy areas are often presented as symmetric ball/cup-shaped curves circumscribed by hilly slopes terrains, and flooded with the runoff rains washed to gorges and downslopes during the rainy seasons. These conditions may form nitrate pollutions, especially when mixed with animal dugs leftover during the dry season while they were grazing. A similar observation was found in the Bonga forest by Nune (2008). Nitrogen in its nature is easily leachable from the soil along sloppy terrains that are exposed to high precipitation (Batjes and Dijkshoorn 1998; Widowati and De Neve 2016).

### Threats to Sele-Nono forests

It is well-known that disturbance influences species composition and diversity of a forest ecosystem. However, not all aspects of disturbances are equally threatening the composition of forest vegetation. Thus, it is essential to know which disturbances are critical to the forest to bring meaningful changes in the conservation aspect (Simberloff 2001; Onaindia et al. 2004). Accordingly, this study found that clearing of forests for cropland expansion by the local people followed by selective removal of trees was the most notorious threat factor in the Sele-Nono forest. Clearing of forests for agricultural expansion is also reported as the main threatening factor accounting for the loss of 60% of tropical moist forests (Wright 2010; Chakravarty et al. 2012). However, this finding contradicts previous reports for other southwest forests of Ethiopia (e.g. Sheka and Bonga forest) that reported the conversion of forest land into commercial tea plantation as a leading threat of the forest ecosystem (Woldu 1999; Woldemariam and Getaneh 2011; Senbeta et al. 2013). This is because unlike the forests in Sheka and Bonga, the conversion of forest land into other commercial investments such as tea plantations in Sele-Nono is prohibited by the rule of the District administration office.

Moreover, the uncontrolled selective removal of trees that was reported as the second main threat in this study is also reported as the main threat to other southwest forests of Ethiopia (Ersado 2001; Abebe 2002). The presence of abundant number of disturbance-associated plant species such as Asteraceae and lianas in the current study may indicate that the area might have faced selective logging. Similar assertions were reported in other moist forests of Ethiopia (Nigatu and Tadesse 1989; Berhan and Assefa 2004; Senbeta et al. 2005; Lulekal et al. 2008) and elsewhere in tropical forests (Putz 1984; Gerwing 2002) who described the abundance of vines and/or Asteraceae plants in openings of a disturbed forest through selective logging. It is said that there was uncontrolled forest exploitation during the past times since the 1950s in the southwest forest mainly for the purpose of timber and timber-related purposes (Chaffey 1979; Ersado 2001; Tadesse and Woldemariam 2007). Most of the species reported in this study to be preferred for timber and timber-related purposes by the local people were also mentioned in other southwest forests of Ethiopia (Chaffey 1979; EFAP 1994; Schmitt et al. 2010; Desalegn et al. 2012; Dibaba et al. 2022). Some of these species are *Pouteria adolfifriederici, Apodytes dimidiate, Celtis africana, Cordia africana, Croton macrostachyus, Ekebergiacapensis, Prunus africana, Olea welwitschii, Polyscias fulva, and Syzygium guineense*. However, the current participatory forest management (PFM) system in the study area is playing a better opportunity in at least controlling the uncontrolled logging practices that have been prevailing in the forest. Moreover, thinning of forests for optimizing coffee bean productivity is also a substantial factor for the degradation of forests in areas where coffee growing is abundant. This threat is also a common phenomenon in other southwest forests of Ethiopia (Friis 1979; Teketay 1999; Woldemariam et al. 2002; Senbeta and Denich 2006; Schmitt et al. 2009; Ango et al. 2020).

Moreover, the finding of this study shows that clearing of forests for settlement expansion due to growing population is another source of threat to the study area. In regard to this finding Yilak and Debelo (2019) indicated that settlement and population growth are the main causes for forest degradation (decline of forest quality) and deforestation in the forests of southwest Ethiopia. On top of this, the recently wide-spreading new road construction projects in the study forest is an emerging threat of plant biodiversity in the Sele-Nono forest as it involves clearing of trees and fragment habitat. Similar observation was found elsewhere (Olander et al. 1998; Ledec and Posas 2003; Bera et al. 2006; Caliskan 2013).On the other hand, over-grazing and fire are among the least notorious threats to the study area. This could be attributed to the moist nature of the forest that could extinguish fire incidences. Moreover, the long rainy season permits rehab the grazed land. These are also reported as the least damaging threat in other moist forests elsewhere (Chakravarty et al. 2012; Tranquilli et al. 2014).

## Conclusion and recommendation

### Conclusion

The present study revealed that the Sele-Nono forest covering 150, 325.27 ha is characterized by mosaic of landscapes. Some of these landscapes have an intact and more protected vegetation as in for example in community types 1 and 4, which may be due to their occurance on suitable habitate and the role of the traditional knowledge of the local people to the forest (also called the KOBO system). On the contrary, some part of the forest especially those that are represented by community type 2 and 6 were more degraded. It can be also concluded that Sele-Nono forest at the landscape level is a reservoir of diverse vascular plants (414 species with high Shannon and beta diversity; H > 3, ${\beta }_{w}$ > 12). Of which 25 of them (6%) were endemic. Given the presence of a high number of plant species along with flagship species such as coffee and other ecological assets it could qualify to be labeled as a keystone ecosystem. Among the environmental factor treated in this study, altitude plays a key ecological role to influence the spatial distribution of plants in the Sele-Nono forest. Human-induced disturbance such as deforestation for cropland expansion and settlements and degradation through selective logging and thinning forest were major threats for the study area.

### Recommendations

1. Sele-Nono forest, being a large forest and rich plant biodiversity shall be developed as a Biosphere Reserve as such system provide the possibility to compartmentalize the forest into core zone, transition zone and buffer zone. This approach further would provide adequate protection to the forest and suitable use of the resources for the communities around the forest.
2. Provision of environmental education to strengthen the existing participatory forest management (PFM) and KOBO system to the local people to minimize uncontrol logging in the forest.
3. The endangered and endemic plant species identified in this study shall be a prior species for conservation by the nearby higher institutions and other plant conservation affiliated research centers.
4. Among the plant community types established in this study, Cluster 2 and 6 shall be among the prioritized area for any possible protection as these areas have been under high level of threat.
5. Designing future vegetation monitoring studies are essential to evaluate the status of the forest and take appropriate actions for any significant changes that may occur in the forest (temporal effect).

## Supplementary Information

**Additional file 1: Appendix S1.** GPS points for sample plots used for the study.

**Additional file 2: Appendix S2.** Modified Braun-Blanquet scale for cover-abundance values (Van der maarel, 2005).

**Additional file 3: Appendix S3.** Checklist used to determine level of disturbance of a sample plot in Sele-Nono forest.

**Additional file 4: Appendix S4.** Semi-structured interview checklist.

**Additional file 5: Appendix S5.** Lists of plant species recorded from Sele-Nono forest.

**Additional file 6: Appendix S6.** new species records to Illubabur (IL) floristic region in the flora of Ethiopia and Eritrea.

**Additional file 7: Appendix S7.** Endemic plant recorded from Sele-Nono forest.

**Additional file 8: Appendix S8.** Outlier species excluded from the analysis.

**Additional file 9: Appendix S9.** Synoptic value of plants species for each clusters.

**Additional file 10: Appendix S10.** Enviromental data recorded for each sample plot in Sele-Nono forest.

## Data Availability

The data used and analyzed in this study can be provided from the respective author for scientifc, non-proft purpose.
