# Supplementary material for: Plant diversity and community analysis of Sele-Nono forest, Southwest Ethiopia: implication for conservation planning
Source: Bot Stud. 2022 Jul 19;63:23. doi: 10.1186/s40529-022-00353-w (PMC9294133; doi:10.1186/s40529-022-00353-w)
Supplement: Supplementary file 1 — Additional file 1: Appendix S1. GPS points for sample plots used for the study. [file 40529_2022_353_MOESM1_ESM.doc]

Appendix 1. GPS points for sample plots used for the study

| Plot No. | Northing | Easting | Altitude | strata number |
| --- | --- | --- | --- | --- |
| 1 | 7^o^47’00’’ | 35^o^15’00’’ | 1640 | 4 |
| 2 | 7^o^53’00’’ | 35^o^14’00’’ | 1165 | 5 |
| 3 | 7^o^48’00’’ | 35^o^16’00’’ | 1640 | 4 |
| 4 | 7^o^43’00’’ | 35^o^09’00’’ | 2400 | 1 |
| 5 | 7^o^47’00’’ | 35^o^06’00’’ | 2240 | 1 |
| 6 | 7^o^58’00’’ | 35^o^16’00’’ | 1640 | 4 |
| 7 | 7^o^45’00’’ | 35^o^10’00’’ | 2430 | 1 |
| 8 | 7^o^52’00’’ | 35^o^26’00’’ | 1872 | 3 |
| 9 | 7^o^49’00’’ | 35^o^08’00’’ | 2180 | 2 |
| 10 | 7^o^57’00’’ | 35^o^17’00’’ | 1740 | 3 |
| 11 | 7^o^54’00’’ | 35^o^24’00’’ | 1620 | 3 |
| 12 | 7^o^57’00’’ | 35^o^07’00’’ | 1830 | 3 |
| 13 | 7^o^46’00’’ | 35^o^09’00’’ | 2380 | 1 |
| 14 | 8^o^06’00’’ | 35^o^08’00’’ | 1430 | 4 |
| 15 | 7^o^56’00’’ | 35^o^23’00’’ | 1850 | 3 |
| 16 | 7^o^46’00’’ | 35^o^17’00’’ | 1640 | 4 |
| 17 | 7^o^57’00’’ | 35^o^14’00’’ | 1230 | 5 |
| 18 | 7^o^59’00’’ | 35^o^16’00’’ | 1280 | 4 |
| 19 | 7^o^56’00’’ | 35^o^19’00’’ | 1880 | 3 |
| 20 | 8^o^02’00’’ | 35^o^15’00’’ | 1100 | 5 |
| 21 | 7^o^42’00’’ | 35^o^12’00’’ | 1860 | 3 |
| 22 | 8^o^05’00’’ | 35^o^17’00’’ | 1200 | 5 |
| 23 | 7^o^58’00’’ | 35^o^10’00’’ | 1820 | 3 |
| 24 | 7^o^51’00’’ | 35^o^08’00’’ | 2010 | 2 |
| 25 | 8^o^04’00’’ | 35^o^17’00’’ | 1600 | 4 |
| 26 | 7^o^58’00’’ | 35^o^14’00’’ | 1190 | 5 |
| 27 | 7^o^45’00’’ | 34^o^58’00’’ | 940 | 6 |
| 28 | 7^o^51’00’’ | 35^o^15’00’’ | 1636 | 4 |
| 29 | 7^o^51’00’’ | 35^o^01’00’’ | 1900 | 3 |
| 30 | 8^o^07’00’’ | 35^o^07’00’’ | 1250 | 5 |
| 31 | 7^o^46’00’’ | 35^o^08’00’’ | 2300 | 1 |
| 32 | 7^o^53’00’’ | 34^o^60’00’’ | 1905 | 3 |
| 33 | 7^o^56’00’’ | 35^o^22’00’’ | 1800 | 3 |
| 34 | 7^o^54’00’’ | 35^o^19’00’’ | 1885 | 3 |
| 35 | 8^o^00’00’’ | 35^o^15’00’’ | 1350 | 5 |
| 36 | 7^o^58’00’’ | 35^o^22’00’’ | 1394 | 4 |
| 37 | 7^o^55’00’’ | 35^o^03’00’’ | 2070 | 2 |
| 38 | 7^o^54’00’’ | 35^o^16’00’’ | 1625 | 4 |
| 39 | 8^o^04’00’’ | 35^o^16’00’’ | 1500 | 4 |
| 40 | 7^o^44’00’’ | 35^o^08’00’’ | 2350 | 1 |
| 41 | 7^o^52’00’’ | 35^o^14’00’’ | 1900 | 2 |
| 42 | 7^o^49’00’’ | 35^o^16’00’’ | 1600 | 4 |
| 43 | 7^o^58’00’’ | 35^o^19’00’’ | 1840 | 3 |
| 44 | 7^o^52’00’’ | 35^o^13’00’’ | 1630 | 4 |
| 45 | 8^o^00’00’’ | 35^o^10’00’’ | 1865 | 3 |
| 46 | 7^o^59’00’’ | 35^o^09’00’’ | 1910 | 3 |
| 47 | 7^o^43’00’’ | 35^o^08’00’’ | 2353 | 1 |
| 48 | 7^o^45’00’’ | 35^o^14’00’’ | 1645 | 4 |
| 49 | 7^o^51’00’’ | 35^o^10’00’’ | 2272 | 1 |
| 50 | 7^o^56’00’’ | 35^o^08’00’’ | 1655 | 3 |
| 51 | 7^o^54’00’’ | 35^o^10’00’’ | 2120 | 2 |
| 52 | 7^o^55’00’’ | 35^o^15’00’’ | 1420 | 4 |
| 53 | 8^o^03’00’’ | 35^o^15’00’’ | 1160 | 5 |
| 54 | 7^o^53’00’’ | 35^o^10’00’’ | 2163 | 2 |
| 55 | 7^o^54’00’’ | 35^o^12’00’’ | 1800 | 2 |
| 56 | 8^o^02’00’’ | 35^o^20’00’’ | 1895 | 3 |
| 57 | 7^o^52’00’’ | 35^o^07’00’’ | 2083 | 2 |
| 58 | 7^o^45’00’’ | 35^o^08’00’’ | 2415 | 1 |
| 59 | 7^o^55’00’’ | 35^o^14’00’’ | 1126 | 5 |
| 60 | 7^o^52’00’’ | 35^o^19’00’’ | 2058 | 2 |
| 61 | 7^o^55’00’’ | 35^o^10’00’’ | 2100 | 2 |
| 62 | 7^o^53’00’’ | 35^o^20’00’’ | 2160 | 2 |
| 63 | 7^o^48’00’’ | 35^o^11’00’’ | 1865 | 3 |
| 64 | 7^o^59’00’’ | 35^o^10’00’’ | 1870 | 3 |
| 65 | 8^o^03’00’’ | 35^o^12’00’’ | 1220 | 5 |
| 66 | 7^o^55’00’’ | 35^o^06’00’’ | 2040 | 2 |
| 67 | 7^o^49’00’’ | 35^o^17’00’’ | 1634 | 4 |
| 68 | 7^o^54’00’’ | 35^o^04’00’’ | 2095 | 2 |
| 69 | 7^o^44’00’’ | 35^o^09’00’’ | 2344 | 1 |
| 70 | 7^o^46’00’’ | 35^o^12’00’’ | 1870 | 3 |
| 71 | 7^o^58’00’’ | 35^o^09’00’’ | 1880 | 3 |
| 72 | 7^o^53’00’’ | 35^o^09’00’’ | 2150 | 2 |
| 73 | 7^o^53’00’’ | 35^o^15’00’’ | 1276 | 5 |
| 74 | 7^o^52’00’’ | 35^o^16’00’’ | 1600 | 4 |
| 75 | 7^o^59’00’’ | 35^o^14’00’’ | 1160 | 5 |
| 76 | 7^o^57’00’’ | 35^o^22’00’’ | 1390 | 4 |
| 77 | 8^o^06’00’’ | 35^o^12’00’’ | 1134 | 5 |
| 78 | 7^o^50’00’’ | 35^o^04’00’’ | 2041 | 2 |
| 79 | 7^o^54’00’’ | 35^o^09’00’’ | 2020 | 2 |
| 80 | 8^o^04’00’’ | 35^o^11’00’’ | 1900 | 2 |
| 81 | 7^o^50’00’’ | 35^o^16’00’’ | 1640 | 4 |
| 82 | 7^o^53’00’’ | 35^o^08’00’’ | 2018 | 2 |
| 83 | 7^o^57’00’’ | 35^o^09’00’’ | 1850 | 3 |
| 84 | 7^o^50’00’’ | 35^o^14’00’’ | 1887 | 2 |
| 85 | 8^o^00’00’’ | 35^o^13’00’’ | 1760 | 2 |
| 86 | 7^o^50’00’’ | 35^o^01’00’’ | 2090 | 2 |
| 87 | 7^o^56’00’’ | 35^o^13’00’’ | 1420 | 4 |
| 88 | 7^o^51’00’’ | 35^o^19’00’’ | 2100 | 2 |
| 89 | 7^o^45’00’’ | 35^o^09’00’’ | 2440 | 1 |
| 90 | 8^o^03’00’’ | 35^o^14’00’’ | 1190 | 5 |
