# Supplementary material for: Plant diversity and community analysis of Sele-Nono forest, Southwest Ethiopia: implication for conservation planning
Source: Bot Stud. 2022 Jul 19;63:23. doi: 10.1186/s40529-022-00353-w (PMC9294133; doi:10.1186/s40529-022-00353-w)
Supplement: Supplementary file 2 — Additional file 2: Appendix S2. Modified Braun-Blanquet scale for cover-abundance values (Van der maarel, 2005). [file 40529_2022_353_MOESM2_ESM.doc]

Appendix 1. Modified Braun-Blanquet scale for cover-abundance values (Van der maarel, 2005)

| Scale | Cover/abundance |
| --- | --- |
| 1 | Rare, generally represented by 1-3 individuals (Cover < 0.5%) |
| 2 | Cover 0.5-1.5% |
| 3 | Cover 1.5-3% |
| 4 | Cover 3-5% |
| 5 | Cover 5-12.5% |
| 6 | Cover 12.5-25% |
| 7 | Cover 25-50% |
| 8 | Cover 50-75% |
| 9 | Cover > 75% |
