# Supplementary material for: Plant diversity and community analysis of Sele-Nono forest, Southwest Ethiopia: implication for conservation planning
Source: Bot Stud. 2022 Jul 19;63:23. doi: 10.1186/s40529-022-00353-w (PMC9294133; doi:10.1186/s40529-022-00353-w)
Supplement: Supplementary file 3 — Additional file 3: Appendix S3. Checklist used to determine level of disturbance of a sample plot in Sele-Nono forest. [file 40529_2022_353_MOESM3_ESM.doc]

Appendix 1. Checklist used to determine level of disturbance of a sample plot in Sele-Nono forest

Date: _________ Northing: __________ Slope: _________ Altitude: __________

Plot no: _______ Easting: ____________ Aspect: _________ Plot features: ____________

Disturbance: _________ (as judged from the following criteria). Note that overall disturbance in a sample plot is calculated from the sum of the scales for each points of disturbance and dividing it by the total number of points of disturbances indicated below in the table.

Please set ranking between 0 (not at all), 1 (very low), low (2), moderate (3), high (4), and 5 (very high) to the following lists of disturbance if occurred in the sample plot

| S/N | Points of disturbance indicator/s | 0 | 1 | 2 | 3 | 4 | 5 | Remarks |
| --- | --- | --- | --- | --- | --- | --- | --- | --- |
| 1 | Evidence of Crops |  |  |  |  |  |  |  |
| 2 | Stumps |  |  |  |  |  |  |  |
| 3 | Fallen woody stems |  |  |  |  |  |  |  |
| 4 | Evidence of trails |  |  |  |  |  |  |  |
| 5 | Browsed or grazed plants |  |  |  |  |  |  |  |
| 6 | Cattle droppings |  |  |  |  |  |  |  |
| 7 | Cattle footprint |  |  |  |  |  |  |  |
| 8 | Evidence of fire (e.g. Burned tree stems) |  |  |  |  |  |  |  |
| 9 | Soil extraction for pottery |  |  |  |  |  |  |  |
| 10 | Diggings for Dioscorea roots or other crops |  |  |  |  |  |  |  |
| 11 | Other (if any) |  |  |  |  |  |  |  |

Note that each points or indicators of disturbance listed in the table above were scaled following Anderson and Currier (1973) with some modification. Each points of disturbance were scaled 0–5, with “0” if the plot was not disturbed with that point of disturbance, “1” when 0-20% of the quadrat disturbed with that specific point of disturbance, “2” when 21-40% of the quadrat disturbed with that particular point of disturbance, “3” if 41-60% of the quadrat disturbed with that specific point of disturbance, “4” if 61- 80% of the quadrat disturbed with that specific point of disturbance, “5” when 81-100% of the quadrat disturbed by that specific point of disturbance.
