# Supplementary material for: Plant diversity and community analysis of Sele-Nono forest, Southwest Ethiopia: implication for conservation planning
Source: Bot Stud. 2022 Jul 19;63:23. doi: 10.1186/s40529-022-00353-w (PMC9294133; doi:10.1186/s40529-022-00353-w)
Supplement: Supplementary file 4 — Additional file 4: Appendix S4. Semi-structured interview checklist. [file 40529_2022_353_MOESM4_ESM.doc]

Appendix 1. Semi-structured interview checklist

Participant code: _______________

Village code: ___________________

Interview date: _________________

1. Personal history: Gender: ______ Age: ______ Language: ________
2. Where are you from originally?
3. Born here (village):_________ (mention the specific village)
4. Not born here: ____________(mention the place or village)
5. If your answer to question number 2 is a, for how long have you lived here? _____________________________________________________
6. If your answer to question number 2 is b, why did you move here? ____________________________________________________
7. How would you describe the importance of the forest to the local people?

____________________________________________________________________________________________________________________________________________________________________________________________________________________________________________________________________________________

1. Has forest coverage in the area changed (e.g. area covered by forest diminished than before)?
2. If yes, Please explain what factors were responsible for this? ____________________________________________________________________________________________________________________________________________________________________________________________________________________________________________________________________________________
3. Do you know exactly which areas (or villages) of the forest are cleared more? _____________________________________________________________________
4. If yes, why? (explain) ____________________________________________________________________________________________________________________________________________________________________________________________________________________________________________________________________________________
5. Has forest quality in the area changed (e.g. more/less trees than before)? __________
6. If yes, what were the main reasons responsible for this? (Please explain your answer)

____________________________________________________________________________________________________________________________________________________________________________________________________________________________________________________________________________________

1. When did these factors started to affect the forest? as far back as you can remember __________________________________________________________________________________________________________________________________________
2. Have your views to disturb the forest changed since you were a child?
3. Yes
4. No
5. Please explain your answer for question number 13? ____________________________________________________________________________________________________________________________________________________________________________________________________________________________________________________________________________________
6. Are there people inside/outside your community trying to convince villagers to conserve forests, other than the government?
7. Yes
8. No
9. If yes, who are they and what do they do? ____________________________________________________________________________________________________________________________________________________________________________________________________________________________________________________________________________________
10. Are there any forest laws /regulations or customs exist in the area (Village/District) that might have played in maintaining the forest coverage? _______________________________________________________________________________________________________________________________________________________________________________________________________________
11. Have your views on these laws/regulations or customary practices changed over time?
12. Yes
13. No
14. If yes; how and why? _______________________________________________________________________________________________________________________________________________________________________________________________________________If no; why? _______________________________________________________________________________________________________________________________________________________________________________________________________________
