# Supplementary material for: Plant diversity and community analysis of Sele-Nono forest, Southwest Ethiopia: implication for conservation planning
Source: Bot Stud. 2022 Jul 19;63:23. doi: 10.1186/s40529-022-00353-w (PMC9294133; doi:10.1186/s40529-022-00353-w)
Supplement: Supplementary file 5 — Additional file 5: Appendix S5. Lists of plant species recorded from Sele-Nono forest. [file 40529_2022_353_MOESM5_ESM.doc]

Appendix 1. Lists of plant species recorded from Sele-Nono forest

| **Family** | **Botanical name** | **Local name** | **GF** | **Remark** |
| --- | --- | --- | --- | --- |
| Acanthaceae | *Acanthopale aethiogermanica* Ensermu | Dergu/Koparo | H | Erect herb |
| Acanthaceae | *Acanthus eminens* C.B.Clarke | Kosoru /Pe'cho | S |  |
| Acanthaceae | *Asystasia gangetica* (L.) T. Anders. subsp *micrantha* (Nees) Ensermu | Dergu | H | Erect herb |
| Acanthaceae | *Barleria ventricosa* Hochst. ex Nees | Dergu/ Kero | H | Erect herb |
| Acanthaceae | *Brillantaisia madagascariensis* T. Anders. | Korro | H | Erect herb |
| Acanthaceae | *Dicliptera maculata* Nees. | Dergu | H | Erect herb |
| Acanthaceae | *Dyschoriste multicaulis* (A.Rich.) O. Kuntze |  | H | Erect herb |
| Acanthaceae | *Hygrophila schulli* (Hamilt.)MR. & S.M Almeida | Bala weranti | H | Erect herb |
| Acanthaceae | *Hypoestes forskaolii* Roem. & Schult. |  | H | Erect herb |
| Acanthaceae | *Hypoestes triflora* (Forssk.) Soland.ex Roem. & Schult. |  | H | Erect herb |
| Acanthaceae | *Isoglossa somalensis* Lindau | Dergu/Kero | H | Erect herb |
| Acanthaceae | *Justicia bizuneshiae* Ensermu |  | H | Erect herb |
| Acanthaceae | *Justicia diclipteroides* Lindau subsp.*aethiopica* Hedren |  | H | Erect herb |
| Acanthaceae | *Justicia ladanoides* Lam. |  | H | Erect herb |
| Acanthaceae | *Justicia schimperiana* T. Anders | Dhumuga/umuga/sheshero | S |  |
| Acanthaceae | *Mellera lobulata* S. Moore | Dergu/Kero | H | Erect herb |
| Acanthaceae | *Monothecium glandulosum* Hochst. |  | S |  |
| Acanthaceae | *Phaulopsis imbricata* (Forssk.) Sweet | Dergu/Kero | H | Erect herb |
| Acanthaceae | *Ruellia prostrata* Poir. |  | H | Erect herb |
| Acanthaceae | *Thunbergia alata* Boj.ex Sims |  | H | Scarmbling herb |
| Acanthaceae | *Whitfieldia elongata* (P.Beauv) De Wild. & T.Dur | Erikoy | S |  |
| Alangiaceae | *Alangium chinense* (Lour)Harms | Shotto | T |  |
| Amaranthaceae | *Achyranthes aspera* L. | Metene | H | Erect herb |
| Amaranthaceae | *Amaranthus hybridus* L. |  | H | Erect herb |
| Amaranthaceae | *Celosia argentea* L. | Ababo | H | Erect herb |
| Amaranthaceae | *Cyathula cylinderica* Moq. | Meten | H | Erect herb |
| Amaranthaceae | *Cyathula polycephala* Bak. | Meten | H | Erect herb |
| Amaranthaceae | *Cyathula prostrata* (L.) Blume | Kero | H | Erect herb |
| Amaranthaceae | *Cyathula uncinulata* (Schard.) Schinz | Bokkoosso | H | Erect herb |
| Amaryllidaceae | *Crinum ornatum* (Ait.) Bury | Kulubi werabesa | H | Erect herb |
| Amaryllidaceae | *Scadoxus nutans* Friis & Nordal | Qicuu | H | Erect herb |
| Anacardiaceae | *Lannea schimperi* (A. Rich.) Engl. |  | T |  |
| Anacardiaceae | *Lannea schweinfurthii* (Engl.) Engl. |  | T |  |
| Anacardiaceae | *Rhus glutinosa* A. Rich. | Xaxesa | S |  |
| Anthericaeae | *Chlorophytum macrophyllum* (A. Rich.) Aschers |  | H | Erect herb |
| Apiaceae | *Centella asiatica* (L.) Urban. | Gura hantuta | H | Erect herb |
| Apiaceae | *Hydrocotyle mannii* Hook.f. | Mijidikkoo | H | Erect herb |
| Apiaceae | *Sanicula elata* Buch.Ham. ex D. Don | Metene | H | Erect herb |
| Apocynaceae | *Alstonia boonei* De Wild. | Joga | T |  |
| Apocynaceae | *Landolphia buchananii* (Hallf ) Stapf | Gebo/yebo | L |  |
| Apocynaceae | *Oncinotis tenuiloba* Stapf. | Hidda gebo qemele | L |  |
| Apocynaceae | *Saba comorensis* (Boj.) Pichon | Gelenchi/Shogaye | L |  |
| Aquifoliaceae | *Ilex mitis* (L.) Radlk. | Qeto | T |  |
| Araceae | *Amorphophallus gallaensis* (Engl.) N.E.Br. | Qicuu | H | Erect herb |
| Araceae | *Arisaema schimperianaum* Schott. | Qicuu | H | Erect herb |
| Araceae | *Colocasia esculatum* (L.)Schott |  | H | Erect herb |
| Araceae | *Culcasia falcifolia* Engl. |  | H | Epiphytic herb |
| Araliaceae | *Polyscias fulva* (Hiern) Harms | Kereso/Keresho | T |  |
| Araliaceae | *Schefflera abyssinica* Harms | Getema/menjo | T |  |
| Araliaceae | *Schefflera myriantha* (Bak.) Drake | Kermato | L |  |
| Araliaceae | *Schefflera volkensii* (Engl.) Harms | Qerro | T |  |
| Arecaceae | *Phoenix reclinata* Jacq. | Mexi | T |  |
| Asclepiadaceae | *Tylophora sylvatica* Decne. |  | L |  |
| Asclepidaceae | *Ceropegia cufodontis* Chiov. |  | H | Scarmbling herb |
| Asclepidaceae | *Leptadeni hastata* (Pers.) Decne |  | H | Scarmbling herb |
| Asparagaceae | *Asparagus africanus* Lam. | Seriti | H | Scarmbling herb |
| Asparagaceae | *Asparagus racemosus* Willd. | Seriti | H/L |  |
| Aspleniaceae | *Aspidium gemmiferum* (Fee) Ching. | Shesso | H | Epiphytic herb |
| Aspleniaceae | *Asplenium aethiopicum* (Burm. F.) Becherer | Shesso | H | Epiphytic herb |
| Aspleniaceae | *Asplenium bugoiense* Hieron. | Shesso | H | Epiphytic herb |
| Aspleniaceae | *Asplenium ceii* Pich.Serm. | Shesso | H | Epiphytic herb |
| Aspleniaceae | *Asplenium elliottii* C.H.Wright | Shesso | H | Epiphytic herb |
| Aspleniaceae | *Asplenium erectum* Bory ex Willd. | Shesso | H | Epiphytic herb |
| Aspleniaceae | *Asplenium friesiorum* C.Chr. | Shesso | H | Epiphytic herb |
| Aspleniaceae | *Asplenium hypomelas* Kuhn | Shesso | H | Epiphytic herb |
| Aspleniaceae | *Asplenium linckii* Kuhn | Shesso | H | Epiphytic herb |
| Aspleniaceae | *Asplenium mannii* Hook. | Shesso | H | Epiphytic herb |
| Aspleniaceae | *Asplenium sandersonii* Hook. | Shesso | H | Epiphytic herb |
| Aspleniaceae | *Asplenium theciferum* (HBK) Mett | Shesso | H | Epiphytic herb |
| Asteraceae | *Acmella caulirhiza* Del. | Guticha gura | H | Erect herb |
| Asteraceae | *Adenostemma mauritianum* DC. |  | H | Erect herb |
| Asteraceae | *Ageratum conyzoides* L. |  | H | Erect herb |
| Asteraceae | *Bidens pilosa* L. |  | H | Erect herb |
| Asteraceae | *Bidens prestinaria* (Sch.Bip.) Cufod. | Adeye Abeba | H | Erect herb |
| Asteraceae | *Bothriocline schimperi* Oliv. & Hiern ex Benth. | Illebu | S |  |
| Asteraceae | *Carduus leptacanthus* Fresen. |  | H | Erect herb |
| Asteraceae | *Conyza agrostophylla* F.G. Davies |  | H | Erect herb |
| Asteraceae | Crassocephalum crepidioides S. Moore |  | H | Erect herb |
| Asteraceae | *Crassocephalum macropappum* S. Moore |  | H | Erect herb |
| Asteraceae | *Crassocephalum montuosum* (S.Moore) Milne-Redh. |  | H | Erect herb |
| Asteraceae | *Dichrocephala integrifolia* O. Kuntze | Areema | H | Erect herb |
| Asteraceae | *Guizotia scabra* (Vis) Chiov. | Haddaa | H | Erect herb |
| Asteraceae | *Guizotia schimperi* Sch.Bip.ex walp. | Tuffo | H | Erect herb |
| Asteraceae | *Helichrysum schimperi* Moesner |  | H | Erect herb |
| Asteraceae | *Lactuca paradoxa* Sch.Bip.ex A. Rich. |  | H | Scarmbling herb |
| Asteraceae | *Laggera crispate* (Vahl.)Hepper & Wood | Nop'ee/Nop'oo | H | Erect herb |
| Asteraceae | *Mikaniopsis clematoides* (Sch. Bip. ex A. Rich.) Milne-Redh. |  | L/S |  |
| Asteraceae | *Solanecio gigas* (Vatke) C.Jeffrey | Debeka | S/T |  |
| Asteraceae | *Solanecio mannii* (Hook.f.)C.Jeffrey | Eqibelo | S/T |  |
| Asteraceae | *Sonchus bipontini* Asch. | Ananna | H | Erect herb |
| Asteraceae | *Tagetes minuta* L. |  | H | Erect herb |
| Asteraceae | *Tridax procumbens* L. |  | H | Scarmbling herb |
| Asteraceae | *Vernonia amygdalina* Del. | Ebicha | S/T |  |
| Asteraceae | *Vernonia auriculifera* Hiern | Reji | S/T |  |
| Asteraceae | *Vernonia hochstetteri* Sch-Bip. | Ilebu kefekatu | S |  |
| Asteraceae | *Vernonia karaguensis* Oliv. & Hiern | Soyema dima | S |  |
| Asteraceae | *Vernonia leopoldi* Vatke | Soyema adi | S |  |
| Asteraceae | *Xanthium strumarium* L. |  | H | Erect herb |
| Balanitaceae | *Balanites aegyptiaca* (L.) Dei. |  | T |  |
| Balsaminaceae | *Impatiens ethiopica* Grey-Wilson | Ensosela kenfekatu/tebecho | H | Erect herb |
| Balsaminaceae | *Impatiens hochstetteri* Warb. | Tebecho | H | Erect herb |
| Balsaminaceae | *Impatiens rothii* Hook.f . |  | H | Erect herb |
| Balsaminaceae | *Impatiens tinctoria* A.Rich. | Ensosiillaa/Tebecho | H | Erect herb |
| Basellaceae | *Basella alba* L. |  | H | Scarmbling herb |
| Begoniaceae | *Begonia wallastonii* Bak. |  | H | Erect herb |
| Bignoniaceae | *Kigelia africana* (Lam.) Benth. |  | T |  |
| Bignoniaceae | *Spathoda campanulata* P.Beauv |  | T |  |
| Bignoniaceae | *Stereospermum kunthianum* Cham. | Botorro | T |  |
| Blechnaceae | *Blechnum tabulare* (Thunb.) Kuhn |  | H | Erect herb |
| Boraginaceae | *Cordia africana* Lam. | Wedessa/Deo | T |  |
| Boraginaceae | *Cynoglossum amplifolium* DC. | Metene adi | H | Erect herb |
| Boraginaceae | *Ehertia cymosa* Thonn. | Ulagga | L/S/T |  |
| Campanulaceae | *Canarina eminii* Schwein. |  | H | Scarmbling herb |
| Campanulaceae | *Lobelia giberroa* Hemsl. | Dumberko/Gederano | T |  |
| Cannaceae | *Canna indica* L. |  | H | Erect herb |
| Capparidaceae | *Gynandropsis gynandra* (L.) Briq. | Rafu mejenger | H | Erect herb |
| Capparidaceae | *Maerua oblongifolia* (Forssk.) A. Rich. |  | T |  |
| Capparidaceae | *Ritchiea albersii* Gilg. | Deqoo | T |  |
| Caryophyllaceae | *Drymaria cordata* (L.) Schultes |  | H | Scarmbling herb |
| Caryophyllaceae | *Stellaria sennii* Chiov. |  | H | Erect herb |
| Celasteraceae | *Catha edulis* (Vahl) Forssk. ex Endl. |  | T |  |
| Celasteraceae | *Elaeodendron buchananii* (Loes.) Loes. | Loko adi/wasso | T |  |
| Celasteraceae | *Hippocratea africana* (Willd) Loes | Hidda galle | L |  |
| Celasteraceae | *Hippocratea goetzei* Loes. | Hidda tiyo | L |  |
| Celasteraceae | *Hippocratea pallens* Planch. Ex oliver | Hidda qawo | L |  |
| Celasteraceae | *Maytenus arbutifolia* (A. Rich.) Wilczek | Qorati/kombolecha | T |  |
| Celasteraceae | *Maytenus gracilipes* (Welw.ex Oliv.) Exell | Qorati/kombolecha | S/T |  |
| Celasteraceae | *Maytenus obscura* (A.Rich.)Cui |  | S/T |  |
| Celasteraceae | *Maytenus undata* (Thunb.) Blakelok | Qorati/kombolecha | S/T |  |
| Combretaceae | *Combretum adenogonium* Steud ex A. Rich. |  | T |  |
| Combretaceae | *Combretum molle* R. Br. ex G. Don |  | T |  |
| Combretaceae | *Combretum paniculatum* Vent. | Begge | L |  |
| Combretaceae | *Terminalia schimperiana* Hochst. | Debeqa | S/T |  |
| Commelinaceae | *Commelina africana* L. | O'ka jebi (yellow) | H | Erect herb |
| Commelinaceae | *Commelina benghalensis* L. | O'ka jebi | H | Erect herb |
| Commelinaceae | *Commelina diffusa* Burm.f. | Qorexobo (Blue) | H | Erect herb |
| Commelinaceae | *Floscopa glomerata* (Willd. ex J.A. Schuf t. & J.H Schuft.) Hassk. |  | H | Erect herb |
| Commelinaceae | *Pollia condensata* C. B. Clarke |  | H | Erect herb |
| Convolvulaceae | *Ipomoea purpurea* (L.)Roth. | Kelala | H | Scarmbling herb |
| Convolvulaceae | *Stiotocardia beraviensis* (Vatke) Hall. F. |  | L |  |
| Costaceae | *Costus afer* Ken-Gawl | Ogiyo Sambiro | H | Erect herb |
| Costaceae | *Costus lucanusianus* J. Braun & K. Schum | Ogiyo Sambiro | H | Erect herb |
| Crassulaceae | *Kalanchoe densiflora* Rolfe. |  | H | Erect herb |
| Cucurbitaceae | *Momordica foetida* Schumach. | Humbaho | H | Scarmbling herb |
| Cucurbitaceae | *Peponium vogelii* (Hook.f.) Engl. | Toojjo (edible) | H | Scarmbling herb |
| Cucurbitaceae | *Zehneria scabra* (Lf) Sond. |  | H | Erect herb |
| Cyatheaceae | *Cyathea manniana* Hook. | Sessino/Gixo | T |  |
| Cyperaceae | *Carex chlorosaccus* C.B. Clarke |  | H | Erect herb |
| Cyperaceae | *Carex thomasii* Nelmes |  | H | Erect herb |
| Cyperaceae | *Coleochloa abyssinica* (Hochst. ex A. Rich.) Gilly |  | H | Erect herb |
| Cyperaceae | *Cyperus dereilema* Steud. | Quni | H | Erect herb |
| Cyperaceae | *Cyperus digitatus* Roxb. | Quni | H | Erect herb |
| Cyperaceae | *Cyperus longibracteatus* (Cherm.) Kük. | Quni | H | Erect herb |
| Cyperaceae | *Cyperus rotundus* L. | Quni | H | Erect herb |
| Cyperaceae | *Cyperus schimperianus* Steud. | Quni | H | Erect herb |
| Dioscoreaceae | *Dioscorea praehensilis* Benth. | Buri mejenger | L |  |
| Dioscoreaceae | *Dioscorea quartiniana* A.Rich. | Muke funo | S/T |  |
| Dracaenaceae | *Dracaena afromontana* Mildbr. | Serxe bala qeqello | S/T |  |
| Dracaenaceae | *Dracaena fragrans* (L.) Ker-Gawl. | Serxe bala Mesengo/Algae/Fishino | T |  |
| Dracaenaceae | *Dracaena steudneri* Scw.ex Engl. | Serxe/Yuddii/yubedi | T |  |
| Dryopteridaceae | *Didymochlaena truncatula* (Swartz) J. Sm. |  | H | Epiphytic herb |
| Dryopteridaceae | *Polystichum transvaalense* N.C. Anthony |  | H | Erect herb |
| Ebenaceae | *Diospyros abyssinica* F. White | Loko guracha/Kuri | T |  |
| Euphorbiaceae | *Acalypha acrogyna* Pax | Derie | S |  |
| Euphorbiaceae | *Acalypha ornata* A. Rich. | Derie | S |  |
| Euphorbiaceae | *Argomuellera macrophylla* Pax | qintebus | S |  |
| Euphorbiaceae | *Bridelia micranta*(Hochst.) Baill. | Rigaraba | S/T |  |
| Euphorbiaceae | *Bridelia scleroneura* Muell. Arg. |  | S |  |
| Euphorbiaceae | *Croton macrostachyus* Del. | Bekenisa/mekenisa/Shomo | T |  |
| Euphorbiaceae | *Erythrococca trichogyne* Prain | Chakko/Bichirkucho | S |  |
| Euphorbiaceae | *Euphorbia ampliphylla* Pax | Adami | T |  |
| Euphorbiaceae | *Macaranga capensis* (Baill.) Sim | O'ngo/werango | T |  |
| Euphorbiaceae | *Phyllanthus limmuensis* Cuf. |  | S |  |
| Euphorbiaceae | *Phyllanthus ovalifolius* Forssk. |  | S |  |
| Euphorbiaceae | *Ricinus communis* L. | Qobbo | S/T |  |
| Euphorbiaceae | *Sapium ellipticum* (Krauss) Pax | Bossoqqa/Sheddo | S/T |  |
| Fabaceae | *Caesalpinia decapetala* (Roth) Alston. | Yeferenj Kontir | H | Scarmbling herb |
| Fabaceae | *Crotalaria brevidens* Benth. | Kishekishe | H | Erect herb |
| Fabaceae | *Crotalaria gillettii* Polhill | Gura wango | H | Erect herb |
| Fabaceae | *Dalbergia lactea* Vatke | Gimero | L |  |
| Fabaceae | *Desmodium repandum* Vahl | Metene | H | Erect herb |
| Fabaceae | *Entada abyssinica* Steud. ex A. Rich. | Ambelta | S |  |
| Fabaceae | *Erythrina abyssinica* (Lam. ex. DC.) |  | T |  |
| Fabaceae | *Glycine wightii* (Wight & Am) Verde. | Kelala hoolla/Kelalo | H | Scarmbling herb |
| Fabaceae | *Indigofera atriceps* Hook.f. |  | S |  |
| Fabaceae | *Piliostigma thonningii* (Schumach.) Milne-Redh. |  | T |  |
| Fabaceae | *Pterolobium stellatum* Brenan | Aregema chaka | H | Scarmbling herb |
| Fabaceae | *Senna petersiana* (Bolle)Lock | Semenek | S/T |  |
| Fabaceae | *Senna septemtrionali* (Viv.) Irwin&Barneby | Semenek | S |  |
| Fabaceae | *Trifolium baccarinii* Chiov. | Sidisa | H | Erect herb |
| Fabaceae | *Trifolium mattirolianum* Chiov. | Sidisa | H | Erect herb |
| Fabaceae | *Vigna membranacea* A. Rich. |  | H | Scarmbling herb |
| Fabaceae | *Vigna vexillata* (L.) A. Rich. | Kelala hoolla | H | Scarmbling herb |
| Fabaceae | *Albizia grandibracteata* Taub. | Ambelta | T |  |
| Fabaceae | *Albizia gummifera* (J. F. Gmel.) C. A. Sm. | Ambebesa | T |  |
| Fabaceae | *Albizia schimperiana* Oliv. | Ambebesa gemoji | T |  |
| Fabaceae | *Baphia abyssinica* Brummitt | Dewie | T |  |
| Fabaceae | *Calpurina aurea* (Ait.) Benth | Ceeka | S |  |
| Fabaceae | *Erythrina brucei* Schweinf. | Wellensu | T |  |
| Fabaceae | *Millettia ferruginea* (Hochst.) Baker | Sotolo/Yaggo | T |  |
| Flacourtiaceae | *Flacourtia indica* (Burm.f.) Merrill | Akuku/shuretto | S/T |  |
| Flacourtiaceae | *Oncoba spinosa* Forssk. | Shurato | S/T |  |
| Geraniaceae | *Geranium arabicum* Forrsk. |  | H | Erect herb |
| Guttiferae | *Garcinia ovalifolia* Oliver | Karawayu | S/T |  |
| Guttiferae | *Garcinia buchananii* Bak. | Gerjeja | S/T |  |
| Hypolepidaceae | *Blotiella glabra* (Bory) R.M. Tryon |  | H | Epiphytic herb |
| Hypolepidaceae | *Pteridium aquilinum* (L.) Kuhn |  | H | Epiphytic herb |
| Icacinaceae | *Apodytes dimidiata* E. Mey. ex. Arn. | Wendebiyo | T |  |
| Lamiaceae | *Achyrospermum parviflorum* S.Moore | Kerro | S |  |
| Lamiaceae | *Achyrospermum schimperi* (Hochst. ex Briq) Perkins (EH) | Korro | S |  |
| Lamiaceae | *Ajuga integrifolia* Buch. Ham. ex D.Don |  | H | Erect herb |
| Lamiaceae | *Ajuga leucantha* Lukhoba |  | H | Erect herb |
| Lamiaceae | *Isodon schimperi* (Vatke)JK. Morton |  | H | Erect herb |
| Lamiaceae | *Leucas calostachys* Oliv. |  | H | Erect herb |
| Lamiaceae | *Ocimum grattissimum* L. | Anchebi | H | Erect herb |
| Lamiaceae | *Ocimum lamiifolium* Hochst ex. Bent. | Demakessie | H/S |  |
| Lamiaceae | *Plectranthus garckeanus* (Vatke) J. K. Morton | Yeriyo/yeriho | H | Erect herb |
| Lamiaceae | *Plectranthus punctatus* (L.f.)L'Her. |  | H | Erect herb |
| Lamiaceae | *Pycnostachys abyssinica* Fresen. |  | H | Erect herb |
| Lamiaceae | *Salvia nilotica* Juss. ex Jacq. |  | H | Erect herb |
| Lamiaceae | *Satureja paradoxa* (Vatke) Engl. | Naddo | H | Erect herb |
| Lauraceae | *Ocotea kenyensis* (Chiov.) Robyns & Wilcz |  | T |  |
| Loganiaceae | *Anthocleista schweinfurthii* Gilg. | Belety | T |  |
| Loganiaceae | *Nuxia congesta* R.Br.ex Fresen. | Qeyisa | T |  |
| Loganiaceae | *Strychnos mitis* S.Moore | Satto/shifu | T |  |
| Lomariopsidaceae | *Elaphoglossum deckenii* (Kuhn) C.Chr. |  | H | Epiphytic herb |
| Lomariopsidaceae | *Elaphoglossum lastii* (Bak.) |  | H | Epiphytic herb |
| Loranthaceae | *Tapinanthus globiferus* (A. Rich.) Tieghem | Dheretu/eretu/harme ada | H | Epiphytic herb |
| Lycopodiaceae | *Huperzia dacrydioides* (Baker ) Pic.Serm. |  | H | Epiphytic herb |
| Lycopodiaceae | *Lycopodiella cernua* (L.) Pic.Serm. |  | H | Erect herb |
| Lycopodiaceae | *Lycopodium clavatum* L. |  | H | Epiphytic herb |
| Malvaceae | *Abutilon longicuspe* Hochst. ex A.Rich. | Qunch | S |  |
| Malvaceae | *Hibiscus berberidifolius* A. Rich | Inchini | S |  |
| Malvaceae | *Hibiscus calyphyllus* Cavan. | Inchini | S |  |
| Malvaceae | *Kosteletzkya begoniifolia* (Ulbr.) Ulbr. | Debessie | S |  |
| Malvaceae | *Pavonia schimperiana* Hochst .ex A.Rich. | Debessie adi | H | Erect herb |
| Malvaceae | *Pavonia urens* Cav. | Debessie dimma | S |  |
| Malvaceae | *Sida rhombifolia* L. | Kereba | H | Erect herb |
| Marantaceae | *Marantochloa leucantha* (K.Schun).Milne-Redh. | Berebere sambiro | H | Erect herb |
| Marattiaceae | *Marattia fraxinea* Sm. | Shesso | H | Epiphytic herb |
| Melastomataceae | *Dissotis senegambiensis* Triana |  | H | Erect herb |
| Melastomataceae | *Tristemma mauritianum* J. F. Gmel |  | H | Erect herb |
| Meliaceae | *Ekebergia capensis* Sparrm. | Sombo/ororo | T |  |
| Meliaceae | *Lepidotrichilia volkensii* (Gurke) Leory | Alele/shawa | T |  |
| Meliaceae | *Pseudocedrela kotschyi* (Schweinf.) Harms |  | T |  |
| Meliaceae | *Trichilia dregeana* Sond. | Luya/yuya | T |  |
| Meliaceae | *Turraea holstii* Guerke |  | S/T |  |
| Melianthaceae | *Bersama abyssinica* Fresen. | Lolchisa/bokko | T |  |
| Menispermaceae | *Cissampelos mucronata* A.Rich. | Kelala/maki mesengo | H | Scarmbling herb |
| Menispermaceae | *Stephania abyssinica* (Dill & A. Rich.) Walp |  | H | Scarmbling herb |
| Menispermaceae | *Tiliacora troupinii* Cufod. | Liqixi/acho | L |  |
| Moraceae | *Antiaris toxicaria* Lesch subsp. *toxicaria* |  | T |  |
| Moraceae | *Ficus exasperata* Vahl. | Balantaie | T |  |
| Moraceae | *Ficus ovata* Vahl. | Dembi guracha/qilinexu | T |  |
| Moraceae | *Ficus sur* Forssk. | Harbu/hexo | T |  |
| Moraceae | *Ficus sycomorus* L. | Dembi gemoji/hexo | T |  |
| Moraceae | *Ficus thonningii* Blume | Dembi shifi | S/T |  |
| Moraceae | *Ficus vasta* Vahl. | Dembi dimma/qilexu | T |  |
| Moraceae | *Milicia excelsa* (Welw.) C. C. Berg |  | T |  |
| Moraceae | *Morus mesozygia* Stapf | Sacho | T |  |
| Moraceae | *Trilepisium madagascariense* DC. | Semeko/Che'ii anneno | T |  |
| Musaceae | *Ensete ventricosum* (Welw.) Cheesman | Qocho sexana/ae'co | H | Erect herb |
| Myrsinaceae | *Embelia schimperi* Vatke |  | L/S |  |
| Myrsinaceae | *Maesa lanceolata* Forssk. | Abeyii/Cheggo | S/T |  |
| Myrsinaceae | *Myrsine africana* L. |  | S |  |
| Myrtaceae | *Psidium guajava* L. |  | S |  |
| Myrtaceae | *Syzygium guineense* (Wild.) DC. Subsp. afromontanum | Beddesa/Yino/Gejo | T |  |
| Nephrolepidaceae | *Nephrolepis biserrata* (Sw.) Schott |  | H | Epiphytic herb |
| Nephrolepidaceae | *Nephrolepis undulata* (Afzel. ex Sw.)J.Sm. |  | H | Epiphytic herb |
| Oleacae | *Jasminum abyssinicum* DC. | Ilchime | L |  |
| Oleaceae | *Chionanthus mildbraedii* Stearn | Shigewo | T |  |
| Oleaceae | *Olea capensis* L.Subsp.macrocarpa (C.A. Wright.) Verdc. | Gegema/shega'o | T |  |
| Oleaceae | *Olea welwitschii* (Knobl.) Gilg & Schellenb | Baha/yaho | T |  |
| Oleandraceae | *Arthropteris monocarpa* (Cordem.) C.Chr. |  | H | Epiphytic herb |
| Onagraceae | *Epilobium stereophyllum* Fresen. |  | H | Erect herb |
| Orchidaceae | *Aerangis brachycarpa* (Rich) Reichb.f. |  | H | Epiphytic herb |
| Orchidaceae | *Aerangis thomsonii* (Rolfe)Schltr |  | H | Epiphytic herb |
| Orchidaceae | *Bulbophyllum intertextum* Lindl. |  | H | Epiphytic herb |
| Orchidaceae | *Bulbophyllum josephii* (Kuntze) Summerh. |  | H | Epiphytic herb |
| Orchidaceae | *Corymborkis corymbis* Thouars |  | H | Erect herb |
| Orchidaceae | *Diaphananthe adoxa* Rasm. |  | H | Epiphytic herb |
| Orchidaceae | *Eulophia guineensis* Lindl. |  | H | Erect herb |
| Orchidaceae | *Habenaria holubii* Rolfe |  | H | Erect herb |
| Orchidaceae | *Polystachya cultriformis* (Thon.) Sprengel |  | H | Epiphytic herb |
| Orchidaceae | *Polystachya steudneri* Rchlo.f |  | H | Epiphytic herb |
| Oxalidaceae | *Oxalis radicosa* A.Rich. |  | H | Erect herb |
| Passifloraceae | *Passiflora edulis* Sims. | Aburuta | H | Scarmbling herb |
| Phytolaccaceae | *Phytolacca dodecandra* L.Herit. |  | L |  |
| Piperaceae | *Peperomia abyssinica* Miq. |  | H | Epiphytic herb |
| Piperaceae | *Peperomia molleri* C. DC. |  | H | Erect herb |
| Piperaceae | *Peperomia retusa* (L.f.) A. Dietr. |  | H | Erect herb |
| Piperaceae | *Peperomia tetraphylla* (Forst.) Hook. & Arn |  | H | Epiphytic herb |
| Piperaceae | *Piper capense* L.f. | Turfo | H | Erect herb |
| Piperaceae | *Piper umbellatum* L. | Tunjo deljessa | H | Erect herb |
| Pittosporaceae | *Pittosporum viridiflorum* Sims | Soole/Shoole | T |  |
| Plantaginaceae | *Plantago lanceolata* L. |  | H | Erect herb |
| Plantaginaceae | *Plantago palmata* Hook.f. |  | H | Erect herb |
| Poaceae | *Arundinaria alpina* K.Schum. | Hoto | H | Erect herb |
| Poaceae | *Digitaria abyssinica* (Hochst.ex A. Rich.)Stapf | Merga | H | Erect herb |
| Poaceae | *Eleusine floccifolia* (Forssk.) Spreng. |  | H | Erect herb |
| Poaceae | *Heteropogon contortus* Roem. & Schult. |  | H | Erect herb |
| Poaceae | *Hyparrhenia pilgeriana* C.E. Hubb. |  | H | Erect herb |
| Poaceae | *Oplismenus hirtellus* (L.) P. Beauv. | Mergagogori/Qemexe/Shuto | H | Erect herb |
| Poaceae | *Oryra latifolia* L. | Go'a | S |  |
| Poaceae | *Panicum atrosanguineum* A. Rich. |  | H | Erect herb |
| Poaceae | *Panicum calvum* Stapf. |  | H | Erect herb |
| Poaceae | *Panicum maximum* Jacq. |  | H | Erect herb |
| Poaceae | *Paspalum scrobiculatum* L. | Merga | H | Erect herb |
| Poaceae | *Pennisetum macroururn* Trin. | Duko | H | Erect herb |
| Poaceae | *Pennisetum trachyphyllum* Pilg. | Duko | H | Erect herb |
| Poaceae | *Setaria megaphylla* (Steud.)Th. Dur. | Jejeba | H | Erect herb |
| Poaceae | *Snowdenia polystachya*(Fresen.) Pilg. | Merga/Muja | H | Erect herb |
| Poaceae | *Sporobolus pyramidalis* P.Beauv. |  | H | Erect herb |
| Podocarpaceae | *Podocarpus falcatus*Thunb. | Birbirsa | T |  |
| Polygonaceae | *Persicaria setosula* (A. Rich.) K.L. Wilson | Wetland herb | H | Erect herb |
| Polygonaceae | *Rumex abyssinicus* Jacq. |  | H | Erect herb |
| Polygonaceae | *Rumex nepalensis* Spreng | Soroto | H | Erect herb |
| Polypodiaceae | *Drynaria volkensii* Hieron | Baala belesa | H | Epiphytic herb |
| Polypodiaceae | *Lepisorus excavatus* (Willd.) Ching. |  | H | Epiphytic herb |
| Polypodiaceae | *Loxogramme abyssinica* (Baker) M.G. Price |  | H | Epiphytic herb |
| Polypodiaceae | *Pleopeltis macrocarpa* (Willd.) Kaul |  | H | Epiphytic herb |
| Pteridaceae | *Pteris dentata* Forssk. |  | H | Epiphytic herb |
| Pteridaceae | *Pteris pteridioides* (Hook.) Ballard |  | H | Erect herb |
| Ranunculaceae | *Clematis hirsuta* Perr. & Guill. | Shego | L |  |
| Ranunculaceae | *Clematis longicauda* Steud.ex A.Rich. | Emano | H | Scarmbling herb |
| Ranunculaceae | *Clematis simensis* Fresen. |  | L |  |
| Ranunculaceae | *Ranunculus multifidus* Forssk. | Gubedu/Qoricha ilekana | H | Erect herb |
| Ranunculaceae | *Thalictrum rhynchocarpum* Dill. & A. Rich | Shererit/Serebizu | H | Erect herb |
| Resedaceae | *Caylusea abyssinica* (Fresen.) Fisch. & Mey |  | H | Erect herb |
| Rhamnaceae | *Gouania longispicta* Engl. | Homecho | L |  |
| Rhamnaceae | *Rhamnus prinoides* L.Herit. | Gesho | S/T |  |
| Rhizophoraceae | *Cassipourea malosana* (Baker) Alston | Loko ququme/Werallo | T |  |
| Rosaceae | *Alchemilla fischeri* Engl. |  | H | Erect herb |
| Rosaceae | *Prunus africana* (Hook.f.) Kalkam | Omi/Omo | T |  |
| Rosaceae | *Rubus apetalus* Poir. | Gora/Injori | S |  |
| Rosaceae | *Rubus steudneri* Schweinf. |  | H | Scarmbling herb |
| Rubiaceae | *Canthium oligocarpum* Hiern | Doqonu/Mesho | S/T |  |
| Rubiaceae | *Coffea arabica* L. | Bunna/Bunno | S/T |  |
| Rubiaceae | *Galiniera saxifraga* (Hochst.) Bridson | Simereru/Diddo | T |  |
| Rubiaceae | *Gardenia ternifolia* Schumach. & Thonn. |  | S/T |  |
| Rubiaceae | *Hallea rubrostipulata* (K.Schum.)J.F.Leroy | Bootto/Ooboo | T |  |
| Rubiaceae | *Oxyanthus speciosus* DC. | Abrango deljesa/O'pero | S/T |  |
| Rubiaceae | *Pavetta abyssinica* Fresen. | Buna qemele/Cheka | S |  |
| Rubiaceae | *Pavetta oliveriana* Hiern | Buna qemele/Cheka | S |  |
| Rubiaceae | *Pentas lanceolata* (Forssk.) Defl. Subsp. lanceolata |  | S |  |
| Rubiaceae | *Psychotria orophila* Petit | Rocha/a'emato/aberango | S/T |  |
| Rubiaceae | *Rothmannia urcelliformis* (Hiern) Robyns | Dibbo | S/T |  |
| Rubiaceae | *Rytigynia neglecta* (Hiern) Robyns | Mixo sere/Nechato | S |  |
| Rubiaceae | *Vangueria madagascariensis* Gmel. | Bururi adi | S |  |
| Rutaceae | *Clausena anisata* (Wild.) Benth. | Ulmayii/eremicho | S |  |
| Rutaceae | *Fagaropsis angolensis* (Engl.) Dale | Muukke/Guraye/yahoo | T |  |
| Rutaceae | *Teclea noblis* Del. | Molaye/Eleke/Kono | T |  |
| Rutaceae | *Vepris dainellii* (Pichi-Serm.)Kokwaro | Haddesa/Mergetto | T |  |
| Rutaceae | *Zanthoxylum usambarense* (Engl.) Kokwaro | Muke-armie | T |  |
| Sapindaceae | *Allophylus abyssinicus* (Hochst.) Radlkofer | Sheo | T |  |
| Sapindaceae | *Allophylus macrobotrys* Gilg | Guresede | T |  |
| Sapindaceae | *Blighia unijugata* Bak. | Ade jebbo | T |  |
| Sapindaceae | *Deinbollia kilimandscharica* Taub. | Kesso | T |  |
| Sapindaceae | *Lecaniodiscus fraxinifolius* Bak. | Keyeni | T |  |
| Sapindaceae | *Paullinia pinnata* L. | Hidda gefersa | L |  |
| Sapotaceae | *Manilkara butugi* Chiov. |  | T |  |
| Sapotaceae | *Mimusops kummel* A.DC. | Qolati/Qoladi | T |  |
| Sapotaceae | *Pouteria adolfi-friederici* (Engl.) Baehni | Qerero/shao | T |  |
| Sapotaceae | *Pouteria alnifolia* (Bak.)Roberty | Gommo | T |  |
| Sapotaceae | *Pouteria altissima* (A.Chev.)Baehni | Semeko/Che'ii anneno | T |  |
| Scrophulariaceae | *Veronica abyssinica* Fres. |  | H | Erect herb |
| Selaginellaceae | *Selaginella kalbreyeri* Bak. |  | H | Epiphytic herb |
| Selaginellaceae | *Selaginella kraussiana* (Kunze) A.Braun |  | H | Epiphytic herb |
| Simaroubaceae | *Brucea antidysenterica* J. F. Mill | Qomegno/Nukasho | T |  |
| Sinopteridaceae | *Doryopteris concolor* (Langsd. & Fisch.) Kuhn |  | H | Epiphytic herb |
| Sinopteridaceae | *Pellaea viridis* (Forssk.) Prantl |  | H | Epiphytic herb |
| Smilacaceae | *Smilax anceps* Willd. |  | L |  |
| Smilaceae | *Smilax aspera* L. |  | H | Scarmbling herb |
| Solanaceae | *Datura stramonium* L. |  | H | Erect herb |
| Solanaceae | *Nicotiana glauca* Graham |  | S |  |
| Solanaceae | *Physalis peruviana* L. | Xossi | H | Erect herb |
| Solanaceae | *Solanium incanum* L | Hiddi | H | Erect herb |
| Solanaceae | *Solanum anguivi* Lam. |  | S |  |
| Solanaceae | *Solanum nigrum* L. | Awiti ken bokkee | H | Erect herb |
| Solanaceae | *Solanum pseudocapsicum* L. |  | H | Erect herb |
| Sterculiaceae | *Dombeya torrida* (J.F.Gmel.)P.Bamps | Adanisa | S/T |  |
| Sterculiaceae | *Sterculia africana* (Lour.) Fiori. |  | T |  |
| Tectariaceae | *Tectaria gemmifera* (Fee) Alston |  | H | Epiphytic herb |
| Thelypteridaceae | *Amauropelta bergiana* (Schltdl.) Holttum |  | H | Epiphytic herb |
| Tilaceae | *Triumfetta rhomboidea* Jacq. |  | S |  |
| Tiliaceae | *Grewia ferruginea* Hochst. ex A.Rich. |  | T |  |
| Tiliaceae | *Grewia mollis* A. Juss. |  | S |  |
| Tiliaceae | *Triumfetta brachyceras* K. Schum. | Inchini | S |  |
| Typhaceae | *Typha latifolia* L. |  | H | Erect herb |
| Ulmaceae | *Celtis africana* Burm. f. |  | T |  |
| Ulmaceae | *Celtis gomphophylla* Bak. |  | T |  |
| Ulmaceae | *Celtis philippensis* Blanco | Chai/Kokaey | T |  |
| Ulmaceae | *Celtis toka* (Forssk.) Hepper & Wood | Chai | T |  |
| Ulmaceae | *Celtis zenkeri* Engl | Kobay | T |  |
| Ulmaceae | *Trema orientalis* (L.) Blume | Sooxoo/huduferda | S/T |  |
| Urticaceae | *Elatostema monticolum* Hook.f | Merga bosonu | H | Erect herb |
| Urticaceae | *Pilea rivularis* Wedd. | Kachi/Kafi | H | Erect herb |
| Urticaceae | *Urera hypselodendron* (A.Rich.)Wedd. | Hidda chepo/Lanqisa | L |  |
| Urticaceae | *Urtica simensis* Steudel | Dobi | H | Erect herb |
| Verbenaceae | *Clerodendrum myricoides* Vatke | Merassisa/Misirch | L |  |
| Verbenaceae | *Lantana trifolia* L. |  | S |  |
| Verbenaceae | *Lippia adoensis* Hochst. ex Walp. | Kusaye | S |  |
| Verbenaceae | *Premna schimperi* Engl. | Uregessa | S |  |
| Verbenaceae | *Verbena officinalis* L. | Arenchy/Arencho | H | Erect herb |
| Violaceae | *Rinorea friisii* M.Gilbert |  | S |  |
| Vitaceae | *Ampelocissus schimperiana* (Hochst ex A.Rich.) | Cheep'oo | H | Scarmbling herb |
| Vitaceae | *Cyphostemma adenocaule* (Steud ex A. Rich.) Desc. ex Wild & Drummond | Hidda renefa | H | Scarmbling herb |
| Vitaceae | *Cyphostemma dembianense* (Chiov.) Vollesen |  | H | Scarmbling herb |
| Vittariaceae | *Antrophyum mannianum* Hook.f. |  | H | Epiphytic herb |
| Vittariaceae | *Vittaria guineensis* Desv |  | H | Epiphytic herb |
| Woodsiaceae | *Athyrium scandicinum* (Willd.) C. Persl. |  | H | Epiphytic herb |
| Woodsiaceae | *Athyrium schimperi* Mouq. ex Fee |  | H | Epiphytic herb |
| Zingiberaceae | *Aframomum corrorima* (Braun) Jansen | Ogiyo | H | Erect herb |
| Zingiberaceae | *Aframomum zambesiacum*(Baker) K. Schum. | Ogiyo deljessa | H | Erect herb |
