# Supplementary material for: Plant diversity and community analysis of Sele-Nono forest, Southwest Ethiopia: implication for conservation planning
Source: Bot Stud. 2022 Jul 19;63:23. doi: 10.1186/s40529-022-00353-w (PMC9294133; doi:10.1186/s40529-022-00353-w)
Supplement: Supplementary file 6 — Additional file 6: Appendix S6. new species records to Illubabur (IL) floristic region in the flora of Ethiopia and Eritrea. [file 40529_2022_353_MOESM6_ESM.doc]

Appendix 1. new species records to Illubabur (IL) floristic region in the flora of Ethiopia and Eritrea

| S/N | Botanical name | Family | Local name | GF | Remark |
| --- | --- | --- | --- | --- | --- |
| 1 | *Aerangis brachycarpa* (Rich) Reichb.f. | Orchidaceae |  | H | Epiphytic herb |
| 2 | *Aerangis thomsonii* (Rolfe)Schltr | Orchidaceae |  | H | Epiphytic herb |
| 3 | *Ageratum conyzoides* L. | Asteraceae |  | H | Erect herb |
| 4 | *Ajuga integrifolia* Buch. Ham. ex D.Don | Lamiaceae |  | H | Erect herb |
| 5 | *Allophylus abyssinicus* (Hochst.) Radlkofer | Sapindaceae | Sheo | T |  |
| 6 | *Amaranthus hybridus* L. | Amaranthaceae |  | H | Erect herb |
| 7 | *Arundinaria alpina* K.Schum. | Poaceae | Hoto | H | Erect herb |
| 8 | *Asparagus africanus* Lam. | Asparagaceae | Seriti | H | Scarmbling herb |
| 9 | *Asparagus racemosus* Willd. | Asparagaceae | Seriti | H/L |  |
| 10 | *Bulbophyllum josephii* (Kuntze) Summerh. | Orchidaceae |  | H | Epiphytic herb |
| 11 | *Caesalpinia decapetala* (Roth) Alston. | Fabaceae | Yeferenj Kontir | H | Scarmbling herb |
| 12 | *Calpurina aurea* (Ait.) Benth | Fabaceae | Ceeka | S |  |
| 13 | *Canarina eminii* Schwein. | Campanulaceae |  | H | Scarmbling herb |
| 14 | *Catha edulis* (Vahl) Forssk. ex Endl. | Celasteraceae |  | T |  |
| 15 | *Ceropegia cufodontis* Chiov. | Asclepidaceae |  | H | Scarmbling herb |
| 16 | *Clematis simensis* Fresen. | Ranunculaceae |  | L |  |
| 17 | *Commelina africana* L. | Commelinaceae | O'ka jebi (yellow) | H | Erect herb |
| 18 | *Desmodium repandum* Vahl | Fabaceae | Metene | H | Erect herb |
| 19 | *Dracaena steudneri* Scw.ex Engl. | Dracaenaceae | Serxe/Yuddii/yubedi | T |  |
| 20 | *Elaeodendron buchananii* (Loes.) Loes. | Celasteraceae | Loko adi/wasso | T |  |
| 21 | *Eleusine floccifolia* (Forssk.) Spreng. | Poaceae |  | H | Erect herb |
| 22 | *Epilobium stereophyllum* Fresen. | Onagraceae |  | H | Erect herb |
| 23 | *Ficus ovata* Vahl. | Moraceae | Dembi guracha/qilinexu | T |  |
| 24 | *Garcinia ovalifolia* Oliver | Guttiferae | Karawayu | S/T |  |
| 25 | *Helichrysum schimperi* Moesner | Asteraceae |  | H | Erect herb |
| 26 | *Impatiens rothii* Hook.f . | Balsaminaceae |  | H | Erect herb |
| 27 | *Impatiens tinctoria* A.Rich. | Balsaminaceae | Ensosiillaa/Tebecho | H | Erect herb |
| 28 | *Indigofera atriceps* Hook.f. | Fabaceae |  | S |  |
| 29 | *Ipomoea purpurea* (L.)Roth. | Convolvulaceae | Kelala | H | Scarmbling herb |
| 30 | *Isodon schimperi* (Vatke)JK. Morton | Lamiaceae |  | H | Erect herb |
| 31 | *Lannea schimperi* (A. Rich.) Engl. | Anacardiaceae |  | T |  |
| 32 | *Lantana trifolia* L. | Verbenaceae |  | S |  |
| 33 | *Leucas calostachys* Oliv. | Lamiaceae |  | H | Erect herb |
| 34 | *Lippia adoensis* Hochst. ex Walp. | Verbenaceae | Kusaye | S |  |
| 35 | *Maytenus undata* (Thunb.) Blakelok | Celasteraceae | Qorati/kombolecha | S/T |  |
| 36 | *Nicotiana glauca* Graham | Solanaceae |  | S |  |
| 37 | *Panicum atrosanguineum* A. Rich. | Poaceae |  | H | Erect herb |
| 38 | *Plantago palmata* Hook.f. | Plantaginaceae |  | H | Erect herb |
| 39 | *Plectranthus garckeanus* (Vatke) J. K. Morton | Lamiaceae | Yeriyo/yeriho | H | Erect herb |
| 40 | *Premna schimperi* Engl. | Verbenaceae | Uregessa | S |  |
| 41 | *Rhus glutinosa* A. Rich. | Anacardiaceae | Xaxesa | S |  |
| 42 | *Rubus steudneri* Schweinf. | Rosaceae |  | H | Scarmbling herb |
| 43 | *Sanicula elata* Buch.Ham. ex D. Don | Apiaceae | Metene | H | Erect herb |
| 44 | *Smilax anceps* Willd. | Smilacaceae |  | L |  |
| 45 | *Smilax aspera* L. | Smilaceae |  | H | Scarmbling herb |
| 46 | *Solanium incanum* L | Solanaceae | Hiddi | H | Erect herb |
| 47 | *Solanum nigrum* L. | Solanaceae | Awiti ken bokkee | H | Erect herb |
| 48 | *Solanum pseudocapsicum* L. | Solanaceae |  | H | Erect herb |
| 49 | *Sporobolus pyramidalis* P.Beauv. | Poaceae |  | H | Erect herb |
| 50 | *Stephania abyssinica* (Dill & A. Rich.) Walp | Menispermaceae |  | H | Scarmbling herb |
| 51 | *Syzygium guineense* (Wild.) DC. Subsp. *afromontanum* | Myrtaceae | Beddesa/Yino/Gejo | T |  |
| 52 | *Tagetes minuta* L. | Asteraceae |  | H | Erect herb |
| 53 | *Trilepisium madagascariense* DC. | Moraceae | Semeko/Che'ii anneno | T |  |
| 54 | *Typha latifolia* L. | Typhaceae |  | H | Erect herb |
| 55 | *Vangueria madagascariensis* Gmel. | Rubiaceae | Bururi adi | S |  |
| 56 | *Verbena officinalis* L. | Verbenaceae | Arenchy/Arencho | H | Erect herb |
| 57 | *Vernonia leopoldi* Vatke | Asteraceae | Soyema adi | S |  |
| 58 | *Veronica abyssinica* Fres. | Scrophulariaceae |  | H | Erect herb |
| 59 | *Zanthoxylum usambarense* (Engl.) Kokwaro | Rutaceae | Muke-armie | T |  |
