# Supplementary material for: Plant diversity and community analysis of Sele-Nono forest, Southwest Ethiopia: implication for conservation planning
Source: Bot Stud. 2022 Jul 19;63:23. doi: 10.1186/s40529-022-00353-w (PMC9294133; doi:10.1186/s40529-022-00353-w)
Supplement: Supplementary file 7 — Additional file 7: Appendix S7. Endemic plant recorded from Sele-Nono forest. [file 40529_2022_353_MOESM7_ESM.doc]

Appendix 1. Endemic plant recorded from Sele-Nono forest

| Family | Botanical name | Local name | Status | GF | Remark |
| --- | --- | --- | --- | --- | --- |
| Acanthaceae | *Justicia bizuneshiae* Ensermu |  |  | H | Erect herb |
| Acanthaceae | *Justicia diclipteroides* Lindau subsp.*aethiopica* Hedren |  |  | H | Erect herb |
| Amaryllidaceae | *Scadoxus nutans* Friis & Nordal | Qicuu |  | H | Erect herb |
| Anacardiaceae | *Rhus glutinosa* A. Rich. | Xaxesa |  | S |  |
| Araceae | *Amorphophallus gallaensis* (Engl.) N.E.Br. | Qicuu |  | H | Erect herb |
| Asteraceae | *Bothriocline schimperi* Oliv. & Hiern ex Benth. | Illebu |  | S |  |
| Asteraceae | *Mikaniopsis clematoides* (Sch. Bip. ex A. Rich.) Milne-Redh. |  | LC | L/S |  |
| Asteraceae | *Solanecio gigas* (Vatke) C.Jeffrey | Debeka | LC | S/T |  |
| Asteraceae | *Vernonia leopoldi* Vatke | Soyema adi |  | S |  |
| Balsaminaceae | *Impatiens rothii* Hook.f . |  |  | H | Erect herb |
| Euphorbiaceae | *Phyllanthus limmuensis* Cuf. |  | VU | S |  |
| Fabaceae | *Crotalaria gillettii* Polhill | Gura wango |  | H | Erect herb |
| Fabaceae | *Trifolium mattirolianum* Chiov. | Sidisa |  | H | Erect herb |
| Fabaceae | *Erythrina brucei* Schweinf. | Wellensu |  | T |  |
| Fabaceae | *Millettia ferruginea* (Hochst.) Baker | Sotolo/Yaggo | LC | T |  |
| Lamiaceae | *Plectranthus garckeanus* (Vatke) J. K. Morton | Yeriyo/yeriho |  | H | Erect herb |
| Lamiaceae | *Pycnostachys abyssinica* Fresen. |  |  | H | Erect herb |
| Lamiaceae | *Satureja paradoxa* (Vatke) Engl. | Naddo |  | H | Erect herb |
| Menispermaceae | *Tiliacora troupinii* Cufod. | Liqixi/acho | VU | L |  |
| Ranunculaceae | *Clematis longicauda* Steud.ex A.Rich. | Emano |  | H | Scarmbling herb |
| Rutaceae | *Vepris dainellii* (Pichi-Serm.)Kokwaro | Haddesa/Mergetto | LC | T |  |
| Urticaceae | *Urtica simensis* Steudel | Dobi |  | H | Erect herb |
| Verbenaceae | *Lippia adoensis* Hochst. ex Walp. | Kusaye | LC | S |  |
| Violaceae | *Rinorea friisii* M.Gilbert |  |  | S |  |
| Zingiberaceae | *Aframomum corrorima* (Braun) Jansen | Ogiyo | VU | H | Erect herb |
