# Supplementary material for: Plant diversity and community analysis of Sele-Nono forest, Southwest Ethiopia: implication for conservation planning
Source: Bot Stud. 2022 Jul 19;63:23. doi: 10.1186/s40529-022-00353-w (PMC9294133; doi:10.1186/s40529-022-00353-w)
Supplement: Supplementary file 8 — Additional file 8: Appendix S8. Outlier species excluded from the analysis. [file 40529_2022_353_MOESM8_ESM.doc]

Appendix 1. Outlier species excluded from the analysis

| S/N | Botanical name | Family |
| --- | --- | --- |
| 1 | *Kigelia africana* (Lam.) Benth. | Bignoniaceae |
| 2 | *Maerua oblongifolia* (Forssk.) A. Rich. | Capparidaceae |
| 3 | *Erythrina abyssinica* (Lam. ex. DC.) | Fabaceae |
| 4 | *Erythrina brucei* Schweinf. | Fabaceae |
| 5 | *Ocimum lamiifolium* Hochst ex. Bent. | Lamiaceae |
| 6 | *Pseudocedrela kotschyi* (Schweinf.) Harms | Meliaceae |
| 7 | *Podocarpus falcatus*Thunb. | Podocarpaceae |
| 8 | *Sterculia africana* (Lour.) Fiori. | Sterculiaceae |
