# Supplementary material for: Plant diversity and community analysis of Sele-Nono forest, Southwest Ethiopia: implication for conservation planning
Source: Bot Stud. 2022 Jul 19;63:23. doi: 10.1186/s40529-022-00353-w (PMC9294133; doi:10.1186/s40529-022-00353-w)
Supplement: Supplementary file 9 — Additional file 9: Appendix S9. Synoptic value of plants species for each clusters. [file 40529_2022_353_MOESM9_ESM.doc]

Appendix 1. Synoptic value of plants species for each clusters

|  | Cluster number | Cluster 1 | Cluster 2 | Cluster 3 | Cluster 4 | Cluster 5 | Cluster 6 | Cluster 7 |
| --- | --- | --- | --- | --- | --- | --- | --- | --- |
| S/N | Cluster size | 19 | 13 | 9 | 29 | 8 | 5 | 7 |
| 1 | *Abutilon longicuspe* | 0.000 | 0.000 | 0.000 | 0.172 | 0.000 | 0.000 | 0.000 |
| 2 | *Acalypha acrogyna* | 0.235 | 0.923 | 0.000 | 0.000 | 0.000 | 0.000 | 0.222 |
| 3 | *Acalypha ornata* | 0.411 | 2.307 | 0.000 | 0.000 | 0.000 | 0.000 | 2.555 |
| 4 | *Acanthopale aethiogermanica* | 0.000 | 0.000 | 0.000 | 0.241 | 0.000 | 0.000 | 0.000 |
| 5 | *Acanthus eminens* | 0.502 | 0.000 | 0.000 | 0.862 | 0.000 | 0.000 | 0.000 |
| 6 | *Achyranthes aspera* | 0.310 | 0.000 | 0.444 | 0.275 | 0.000 | 0.000 | 0.555 |
| 7 | *Achyrospermum parviflorum* | 0.000 | 0.153 | 0.000 | 0.034 | 0.000 | 0.000 | 0.000 |
| 8 | *Achyrospermum schimperi* | 0.000 | 0.272 | 0.000 | 0.068 | 0.000 | 0.400 | 0.000 |
| 9 | *Acmella caulirhiza* | 0.000 | 0.000 | 3.111 | 0.103 | 0.000 | 0.000 | 0.000 |
| 10 | *Adenostemma mauritianum* | 0.000 | 0.000 | 0.000 | 0.000 | 0.875 | 0.000 | 0.000 |
| 11 | *Aframomum corrorima* | 2.705 | 0.000 | 0.000 | 0.137 | 0.000 | 0.000 | 1.22 |
| 12 | *Aframomum zambesiacum* | 0.941 | 0.076 | 0.000 | 0.000 | 0.000 | 0.000 | 0.000 |
| 13 | *Ageratum conyzoides* | 0.000 | 0.383 | 0.000 | 0.000 | 0.750 | 0.000 | 0.000 |
| 14 | *Ajuga integrifolia* | 0.000 | 0.000 | 0.255 | 0.034 | 0.000 | 0.000 | 0.000 |
| 15 | *Ajuga leucantha* | 0.000 | 0.000 | 0.000 | 0.034 | 0.500 | 0.000 | 0.000 |
| 16 | *Alangium chinense* | 0.294 | 0.000 | 0.000 | 0.172 | 0.000 | 0.000 | 0.000 |
| 17 | *Albizia grandibracteata* | 1.941 | 1.461 | 0.000 | 0.137 | 0.000 | 0.000 | 2.888 |
| 18 | *Albizia gummifera* | 0.882 | 0.384 | 0.000 | 1.275 | 0.000 | 0.000 | 0.888 |
| 19 | *Albizia schimperiana* | 0.000 | 2.307 | 0.000 | 0.000 | 0.000 | 0.000 | 0.555 |
| 20 | *Alchemilla ellenbeckii* | 0.000 | 0.000 | 0.000 | 0.000 | 0.625 | 0.000 | 0.000 |
| 21 | *Alchemilla fischeri* | 0.000 | 0.000 | 0.000 | 0.103 | 1.750 | 0.000 | 0.000 |
| 22 | *Allophylus abyssinicus* | 0.941 | 0.000 | 1.033 | 0.827 | 0.000 | 0.000 | 0.000 |
| 23 | *Allophylus macrobotrys* | 0.000 | 1.846 | 0.000 | 0.000 | 0.000 | 0.000 | 0.000 |
| 24 | *Alstonia boonei* | 0.000 | 5.000 | 0.000 | 0.000 | 0.000 | 0.000 | 1.444 |
| 25 | *Amauropelta bergiana* | 0.000 | 0.000 | 1.888 | 0.000 | 0.000 | 0.000 | 0.000 |
| 26 | *Amorphophallus gallaensis* | 0.000 | 0.000 | 0.000 | 0.068 | 0.000 | 0.000 | 0.000 |
| 27 | *Ampelocissus schimperiana* | 0.000 | 0.461 | 0.000 | 0.068 | 0.000 | 0.000 | 0.000 |
| 28 | *Anthocleista schweinfurthii* | 0.000 | 1.384 | 0.000 | 0.000 | 0.000 | 0.000 | 3.777 |
| 29 | *Antiaris toxicaria* | 0.000 | 0.615 | 0.000 | 0.000 | 0.000 | 0.000 | 0.000 |
| 30 | *Apodytes dimidiata* | 0.000 | 0.769 | 0.000 | 2.655 | 0.000 | 1.048 | 0.000 |
| 31 | *Argomuellera macrophylla* | 0.000 | 3.230 | 0.000 | 0.000 | 0.000 | 3.600 | 2.071 |
| 32 | *Arisaema schimperianaum* | 0.000 | 0.000 | 0.000 | 0.137 | 0.000 | 0.000 | 0.666 |
| 33 | *Arthropteris monocarpa* | 0.000 | 0.000 | 0.666 | 1.983 | 0.750 | 0.000 | 0.000 |
| 34 | *Arundinaria alpina* | 0.000 | 0.000 | 7.666 | 0.000 | 0.000 | 0.000 | 0.000 |
| 35 | *Asparagus africanus* | 0.000 | 0.000 | 0.000 | 0.034 | 0.000 | 0.000 | 0.000 |
| 36 | *Asparagus racemosus* | 0.000 | 0.000 | 1.333 | 0.034 | 0.000 | 0.000 | 0.000 |
| 37 | *Asplenium sandersonii* | 0.470 | 0.000 | 0.603 | 0.137 | 0.000 | 0.000 | 0.000 |
| 38 | *Asystasia gangetica*subsp *micrantha* | 0.117 | 0.461 | 0.000 | 0.103 | 0.000 | 0.000 | 0.000 |
| 39 | *Athyrium scandicinum* | 0.000 | 0.000 | 0.000 | 0.034 | 0.000 | 0.000 | 0.000 |
| 40 | *Athyrium schimperi* | 0.000 | 0.000 | 2.222 | 0.000 | 0.000 | 0.000 | 0.000 |
| 41 | *Baphia abyssinica* | 0.000 | 2.461 | 0.000 | 0.000 | 0.000 | 0.000 | 2.362 |
| 42 | *Barleria ventricosa* | 0.058 | 0.384 | 0.000 | 0.206 | 0.000 | 0.000 | 0.000 |
| 43 | *Basella alba* | 0.000 | 0.000 | 0.000 | 0.000 | 0.500 | 0.000 | 0.000 |
| 44 | *Begonia wollastonii* | 0.000 | 0.000 | 0.000 | 0.103 | 0.000 | 0.000 | 0.000 |
| 45 | *Bersama abyssinica* | 1.294 | 0.000 | 1.084 | 1.620 | 0.000 | 4.000 | 0.000 |
| 46 | *Bidens prestinaria* | 0.000 | 0.000 | 3.333 | 0.000 | 0.000 | 0.000 | 0.000 |
| 47 | *Blechnum tabulare* | 0.000 | 0.000 | 0.000 | 0.000 | 0.625 | 0.000 | 0.000 |
| 48 | *Blighia unijugata* | 0.000 | 1.230 | 0.000 | 0.000 | 0.000 | 0.000 | 1.666 |
| 49 | *Blotiella glabra* | 0.000 | 0.000 | 1.777 | 0.000 | 0.000 | 0.000 | 0.000 |
| 50 | *Bridelia micranta* | 0.000 | 0.000 | 0.000 | 0.000 | 0.000 | 1.089 | 0.555 |
| 51 | *Brillantaisia madagascariensis* | 0.000 | 0.000 | 0.000 | 0.482 | 0.000 | 0.000 | 0.000 |
| 52 | *Brucea antidysenterica* | 0.588 | 0.000 | 0.000 | 0.482 | 0.000 | 1.620 | 0.000 |
| 53 | *Calpurina aurea* | 0.058 | 0.000 | 0.000 | 0.000 | 0.000 | 0.000 | 0.000 |
| 54 | *Canthium oligocarpum* | 0.235 | 0.000 | 0.000 | 0.862 | 0.000 | 0.000 | 0.000 |
| 55 | *Carex chlorosaccus* | 0.058 | 0.000 | 0.000 | 0.000 | 0.000 | 0.000 | 0.000 |
| 56 | *Carex thomasii* | 0.000 | 0.000 | 0.000 | 0.000 | 1.250 | 0.000 | 0.000 |
| 57 | *Cassipourea malosana* | 0.000 | 0.000 | 0.000 | 0.000 | 0.000 | 0.000 | 0.777 |
| 58 | *Caylusea abyssinica* | 0.000 | 0.000 | 0.000 | 0.000 | 0.750 | 0.000 | 0.000 |
| 59 | *Celosia argentea* | 0.000 | 0.000 | 0.000 | 0.034 | 0.000 | 1.800 | 0.000 |
| 60 | *Celtis africana* | 0.000 | 0.230 | 0.000 | 0.620 | 0.000 | 0.000 | 0.000 |
| 61 | *Celtis gomphophylla* | 0.000 | 0.692 | 0.000 | 0.000 | 0.000 | 0.000 | 0.000 |
| 62 | *Celtis philippensis* | 0.000 | 2.153 | 0.000 | 0.000 | 0.000 | 0.000 | 1.000 |
| 63 | *Celtis zenkeri* | 0.000 | 1.461 | 0.000 | 0.000 | 0.000 | 0.000 | 0.000 |
| 64 | *Centella asiatica* | 0.000 | 0.000 | 2.666 | 0.241 | 0.000 | 0.000 | 0.000 |
| 65 | *Ceropegia cufodontis* | 0.000 | 0.000 | 0.000 | 0.000 | 0.000 | 0.400 | 0.111 |
| 66 | *Chionanthus mildbraedii* | 0.000 | 0.000 | 0.000 | 0.137 | 0.000 | 0.000 | 0.000 |
| 67 | *Chlorophytum macrophyllum* | 0.000 | 0.000 | 0.000 | 0.000 | 0.000 | 0.600 | 0.333 |
| 68 | *Cissampelos mucronata* | 0.000 | 0.000 | 0.000 | 0.034 | 0.000 | 0.000 | 0.704 |
| 69 | *Clausena anisata* | 0.352 | 0.076 | 0.000 | 0.862 | 0.000 | 0.000 | 0.000 |
| 70 | *Clematis hirsuta* | 0.000 | 0.153 | 0.000 | 0.000 | 0.000 | 0.000 | 0.000 |
| 71 | *Clematis longicauda* | 0.000 | 0.153 | 0.000 | 0.034 | 0.000 | 0.000 | 0.000 |
| 72 | *Clematis simensis* | 0.058 | 0.000 | 0.000 | 0.000 | 0.000 | 0.000 | 0.000 |
| 73 | *Clerodendrum myricoides* | 0.352 | 0.000 | 0.000 | 0.103 | 0.000 | 0.000 | 0.000 |
| 74 | *Coffea arabica* | 2.647 | 1.923 | 0.000 | 0.000 | 0.000 | 2.600 | 1.888 |
| 75 | *Combretum paniculatum* | 0.411 | 0.000 | 0.000 | 0.448 | 0.000 | 1.200 | 0.555 |
| 76 | *Commelina africana* | 0.000 | 0.000 | 0.444 | 0.103 | 0.000 | 0.000 | 0.000 |
| 77 | *Commelina benghalensis* | 0.000 | 0.000 | 0.111 | 0.103 | 0.000 | 0.000 | 0.000 |
| 78 | *Commelina diffusa* | 0.000 | 0.000 | 0.000 | 0.034 | 0.000 | 0.000 | 0.000 |
| 79 | *Conyza agrostophylla* | 0.000 | 0.000 | 0.000 | 0.034 | 0.375 | 0.000 | 0.000 |
| 80 | *Cordia africana* | 3.294 | 0.769 | 0.000 | 0.620 | 0.000 | 4.600 | 1.666 |
| 81 | *Costus afer* | 0.000 | 0.461 | 0.000 | 0.000 | 0.000 | 0.000 | 0.444 |
| 82 | *Costus lucanusianus* | 0.000 | 0.000 | 0.000 | 0.000 | 0.000 | 0.000 | 0.555 |
| 83 | *Crassocephalum crepidioides* | 0.117 | 0.000 | 0.000 | 0.000 | 0.000 | 0.000 | 0.000 |
| 84 | *Crassocephalum macropappum* | 0.235 | 0.000 | 0.000 | 0.251 | 0.000 | 0.000 | 0.000 |
| 85 | *Crassocephalum montuosum* | 0.000 | 0.000 | 0.000 | 0.103 | 0.000 | 0.000 | 0.000 |
| 86 | *Crinum ornatum* | 0.294 | 0.000 | 0.000 | 0.000 | 0.000 | 0.000 | 0.000 |
| 87 | *Crotalaria brevidens* | 0.000 | 0.000 | 0.000 | 0.034 | 0.000 | 0.000 | 0.000 |
| 88 | *Crotalaria gillettii* | 0.000 | 0.000 | 0.000 | 0.034 | 0.000 | 0.000 | 0.000 |
| 89 | *Croton macrostachyus* | 1.352 | 0.000 | 0.000 | 1.517 | 0.000 | 2.053 | 0.000 |
| 90 | *Culcasia falcifolia* | 0.000 | 0.000 | 0.000 | 0.172 | 0.000 | 1.000 | 0.000 |
| 91 | *Cyathula cylinderica* | 0.000 | 0.000 | 0.000 | 0.034 | 0.000 | 0.000 | 0.000 |
| 92 | *Cyathea manniana* | 0.000 | 0.000 | 0.000 | 0.413 | 0.000 | 0.000 | 0.000 |
| 93 | *Cyathula polycephala* | 0.000 | 0.000 | 0.333 | 0.000 | 0.375 | 0.000 | 0.000 |
| 94 | *Cyathula prostrata* | 0.000 | 0.000 | 0.111 | 0.000 | 0.000 | 0.000 | 0.000 |
| 95 | *Cyathula uncinulata* | 0.000 | 0.000 | 0.000 | 0.103 | 0.000 | 0.000 | 0.000 |
| 96 | *Cynoglossum amplifolium* | 0.058 | 0.000 | 0.222 | 0.172 | 0.000 | 0.000 | 0.000 |
| 97 | *Cyperus aterrimus* | 0.000 | 0.000 | 0.000 | 0.000 | 0.500 | 0.000 | 0.000 |
| 98 | *Cyperus dereilema* | 0.000 | 0.000 | 0.000 | 0.068 | 5.375 | 0.000 | 0.000 |
| 99 | *Cyperus digitatus* | 0.058 | 0.000 | 0.000 | 0.000 | 0.000 | 0.000 | 0.222 |
| 100 | *Cyperus longibracteatus* | 0.000 | 0.000 | 0.777 | 0.000 | 5.500 | 0.000 | 0.000 |
| 101 | *Cyperus rotundus* | 0.000 | 0.000 | 0.000 | 0.137 | 0.000 | 0.000 | 1.111 |
| 102 | *Cyperus schimperianus* | 0.000 | 0.000 | 0.000 | 0.103 | 0.000 | 0.000 | 0.000 |
| 103 | *Cyphostemma adenocaule* | 0.235 | 0.000 | 0.000 | 0.137 | 0.000 | 0.000 | 0.000 |
| 104 | *Cyphostemma dembianense* | 0.058 | 0.000 | 0.000 | 0.000 | 0.000 | 0.000 | 0.000 |
| 105 | *Dalbergia lactea* | 0.000 | 0.076 | 0.000 | 0.992 | 0.000 | 0.000 | 0.000 |
| 106 | *Deinbollia kilimandscharica* | 1.235 | 0.000 | 0.000 | 3.103 | 0.000 | 1.000 | 0.000 |
| 107 | *Desmodium repandum* | 0.352 | 0.000 | 1.777 | 1.551 | 0.000 | 0.000 | 1.000 |
| 108 | *Dichrocephala integrifolia* | 0.000 | 0.000 | 0.666 | 0.137 | 0.500 | 0.000 | 0.000 |
| 109 | *Dicliptera maculata* | 0.058 | 0.000 | 0.000 | 0.034 | 0.000 | 0.000 | 0.000 |
| 110 | *Digitaria abyssinica* | 0.000 | 0.307 | 0.000 | 0.000 | 0.625 | 0.000 | 0.000 |
| 111 | *Diospyros abyssinica* | 2.235 | 1.923 | 0.000 | 0.620 | 0.000 | 4.000 | 3.111 |
| 112 | *Dioscorea praehensilis* | 0.000 | 0.000 | 0.000 | 0.000 | 0.000 | 0.000 | 0.222 |
| 113 | *Dissotis senegambiensis* | 0.000 | 0.000 | 0.000 | 0.326 | 0.125 | 0.000 | 0.000 |
| 114 | *Dombeya torrida* | 0.000 | 0.000 | 0.000 | 0.172 | 0.000 | 0.000 | 0.000 |
| 115 | *Doryopteris concolor* | 0.823 | 0.000 | 0.000 | 0.482 | 0.000 | 2.200 | 0.777 |
| 116 | *Dracaena afromontana* | 0.294 | 0.000 | 0.988 | 4.413 | 0.000 | 0.000 | 0.000 |
| 117 | *Dracaena fragrans* | 1.294 | 1.692 | 0.000 | 0.103 | 0.000 | 4.600 | 3.666 |
| 118 | *Dracaena steudneri* | 2.000 | 0.000 | 0.000 | 0.172 | 0.000 | 2.018 | 4.666 |
| 119 | *Drymaria cordata* | 0.000 | 0.000 | 0.555 | 0.000 | 0.000 | 0.000 | 0.000 |
| 120 | *Dyschoriste multicaulis* | 0.117 | 0.000 | 0.000 | 0.034 | 0.000 | 0.000 | 0.000 |
| 121 | *Ehertia cymosa* | 1.117 | 0.000 | 0.000 | 0.068 | 0.000 | 2.066 | 0.000 |
| 122 | *Ekebergia capensis* | 0.647 | 0.000 | 0.000 | 1.103 | 0.000 | 0.000 | 0.000 |
| 123 | *Elaeodendron buchananii* | 3.941 | 0.307 | 0.000 | 2.517 | 0.000 | 0.000 | 0.666 |
| 124 | *Elatostema monticolum* | 0.411 | 0.000 | 0.000 | 1.310 | 1.125 | 0.000 | 1.333 |
| 125 | *Eleusine floccifolia* | 0.000 | 0.000 | 0.000 | 0.034 | 0.000 | 0.000 | 0.000 |
| 126 | *Embelia schimperi* | 0.502 | 0.076 | 0.000 | 0.817 | 0.000 | 0.000 | 0.000 |
| 127 | *Ensete ventricosum* | 0.117 | 0.076 | 0.000 | 0.034 | 0.000 | 0.000 | 0.333 |
| 128 | *Entada abyssinica* | 1.823 | 0.846 | 0.000 | 0.103 | 0.000 | 0.000 | 1.444 |
| 129 | *Epilobium stereophyllum* | 0.000 | 0.000 | 0.666 | 0.000 | 0.625 | 0.000 | 0.000 |
| 130 | *Erythrococca trichogyne* | 1.647 | 0.000 | 0.000 | 0.000 | 0.000 | 2.036 | 1.111 |
| 131 | *Eulophia guineensis* | 0.000 | 0.000 | 0.000 | 0.000 | 0.000 | 0.000 | 0.333 |
| 132 | *Euphorbia ampliphylla* | 0.000 | 0.000 | 0.000 | 0.103 | 0.000 | 2.011 | 0.000 |
| 133 | *Fagaropsis angolensis* | 0.764 | 0.307 | 0.000 | 0.344 | 0.000 | 0.000 | 0.000 |
| 134 | *Ficus exasperata* | 0.000 | 0.769 | 0.000 | 0.000 | 0.000 | 0.209 | 1.444 |
| 135 | *Ficus ovata* | 1.176 | 0.384 | 0.000 | 0.000 | 0.000 | 2.061 | 2.333 |
| 136 | *Ficus sur* | 1.058 | 0.384 | 0.000 | 2.068 | 0.000 | 2.320 | 1.222 |
| 137 | *Ficus sycomoru* | 0.176 | 1.076 | 0.000 | 0.000 | 0.000 | 2.000 | 0.444 |
| 138 | *Ficus thonningii* | 0.470 | 0.000 | 0.000 | 0.000 | 0.000 | 1.870 | 0.777 |
| 139 | *Ficus vasta* | 0.941 | 0.000 | 0.000 | 0.000 | 0.000 | 2.061 | 0.000 |
| 140 | *Flacourtia indica* | 0.000 | 0.000 | 0.000 | 0.689 | 0.000 | 0.000 | 0.000 |
| 141 | *Floscopa glomerata* | 0.000 | 0.000 | 0.555 | 0.034 | 0.750 | 0.000 | 0.000 |
| 142 | *Galiniera saxifraga* | 0.588 | 0.000 | 1.202 | 1.517 | 0.000 | 0.000 | 0.000 |
| 143 | *Garcinia buchananii* | 0.000 | 1.461 | 0.000 | 0.000 | 0.000 | 0.000 | 0.000 |
| 144 | *Garcinia ovalifolia* | 0.000 | 2.230 | 0.000 | 0.000 | 0.000 | 0.000 | 0.000 |
| 145 | *Geranium arabicum* | 0.000 | 0.000 | 0.000 | 0.034 | 0.000 | 0.000 | 0.000 |
| 146 | *Glycine wightii* | 0.000 | 0.000 | 0.000 | 0.068 | 0.000 | 0.000 | 0.000 |
| 147 | *Gouania longispicta* | 0.000 | 0.000 | 0.000 | 0.137 | 0.000 | 1.800 | 0.333 |
| 148 | *Guizotia scabra* | 0.000 | 2.130 | 1.001 | 0.034 | 0.000 | 0.000 | 0.000 |
| 149 | *Guizotia schimperi* | 0.000 | 0.000 | 1.333 | 0.000 | 0.000 | 0.000 | 0.000 |
| 150 | *Gynandropsis gynandra* | 0.000 | 0.153 | 0.000 | 0.000 | 0.000 | 0.000 | 0.000 |
| 151 | *Habenaria holubii* | 0.000 | 0.000 | 0.000 | 0.820 | 0.625 | 0.000 | 0.000 |
| 152 | *Hallea rubrostipulata* | 1.024 | 0.000 | 0.000 | 0.344 | 0.000 | 1.000 | 0.000 |
| 153 | *Helichrysum formosissimum* | 0.000 | 0.000 | 0.000 | 0.000 | 0.500 | 0.000 | 0.000 |
| 154 | *Heteropogon contortus* | 0.000 | 0.000 | 0.000 | 0.000 | 0.500 | 0.000 | 0.000 |
| 155 | *Hippocratea africana* | 0.941 | 0.000 | 0.000 | 1.001 | 0.000 | 1.000 | 0.777 |
| 156 | *Hippocratea goetzei* | 1.388 | 0.461 | 0.000 | 0.000 | 0.000 | 1.610 | 1.000 |
| 157 | *Hippocratea pallens* | 1.176 | 0.307 | 0.000 | 0.793 | 0.000 | 0.000 | 1.222 |
| 158 | *Hydrocotyle mannii* | 0.000 | 0.000 | 0.000 | 0.068 | 0.000 | 0.000 | 0.000 |
| 159 | *Hygrophila schulli* | 0.000 | 0.000 | 0.000 | 0.034 | 0.750 | 0.000 | 0.000 |
| 160 | *Hyparrhenia pilgeriana* | 0.000 | 0.000 | 0.000 | 0.000 | 0.750 | 0.000 | 0.000 |
| 161 | *Hypoestes forskaolii* | 0.550 | 0.000 | 0.000 | 0.103 | 0.000 | 0.000 | 0.000 |
| 162 | *Hypoestes triflora* | 0.701 | 0.000 | 0.666 | 0.103 | 0.000 | 0.000 | 0.000 |
| 163 | *Ilex mitis* | 0.470 | 0.000 | 0.000 | 1.241 | 0.000 | 0.000 | 0.000 |
| 164 | *Impatiens ethiopica* | 0.000 | 0.000 | 0.444 | 0.103 | 0.000 | 0.000 | 0.000 |
| 165 | *Impatiens hochstetteri* | 0.000 | 0.957 | 0.333 | 0.172 | 0.000 | 0.000 | 0.000 |
| 166 | *Impatiens tinctoria* | 0.000 | 0.000 | 0.000 | 0.103 | 0.000 | 0.000 | 0.000 |
| 167 | *Indigofera atriceps* | 0.058 | 0.000 | 0.111 | 0.000 | 0.000 | 0.000 | 0.000 |
| 168 | *Ipomoea purpurea* | 0.235 | 0.000 | 0.000 | 0.000 | 0.000 | 0.000 | 0.000 |
| 169 | *Isodon schimperi* | 0.000 | 0.505 | 0.000 | 0.034 | 0.000 | 0.000 | 0.000 |
| 170 | *Isoglossa somalensis* | 0.511 | 0.000 | 0.000 | 0.206 | 0.000 | 0.000 | 0.000 |
| 171 | *Jasminum abyssinicum* | 0.117 | 0.076 | 0.000 | 0.275 | 0.000 | 0.000 | 0.000 |
| 172 | *Justicia bizuneshiae* | 0.117 | 0.000 | 0.000 | 0.103 | 0.000 | 0.000 | 0.707 |
| 173 | *Justicia diclipteroides*subsp*.aethiopica* | 0.000 | 0.000 | 0.000 | 0.068 | 0.000 | 0.000 | 0.000 |
| 174 | *Justicia ladanoides* | 0.117 | 0.384 | 0.000 | 0.034 | 0.000 | 0.000 | 0.600 |
| 175 | *Justicia schimperiana* | 0.000 | 0.000 | 0.000 | 0.827 | 0.000 | 0.000 | 0.000 |
| 176 | *Lactuca paradoxa* | 0.000 | 0.000 | 0.000 | 0.103 | 0.000 | 0.000 | 0.000 |
| 177 | *Laggera crispate* | 0.000 | 0.000 | 0.000 | 0.137 | 0.000 | 0.000 | 0.000 |
| 178 | *Landolphia buchananii* | 1.647 | 0.000 | 0.000 | 1.103 | 0.000 | 0.000 | 0.000 |
| 179 | *Lantana trifolia* | 0.117 | 0.000 | 0.000 | 0.000 | 0.000 | 0.000 | 0.000 |
| 180 | *Lecaniodiscus fraxinifolius* | 0.000 | 1.384 | 0.000 | 0.000 | 0.000 | 0.000 | 0.000 |
| 181 | *Lepidotrichilia volkensii* | 0.235 | 0.000 | 0.000 | 1.689 | 0.000 | 1.600 | 0.000 |
| 182 | *Leptadeni hastata* | 0.000 | 0.000 | 0.000 | 0.000 | 0.000 | 0.000 | 0.222 |
| 183 | *Leucas calostachys* | 0.000 | 0.000 | 0.000 | 0.000 | 0.000 | 0.000 | 0.111 |
| 184 | *Lippia adoensis* | 0.000 | 0.000 | 0.000 | 0.034 | 0.000 | 0.000 | 0.000 |
| 185 | *Lobelia giberroa* | 0.820 | 0.000 | 0.000 | 0.068 | 0.000 | 0.000 | 0.000 |
| 186 | *Lycopodiella cernua* | 0.000 | 0.000 | 0.000 | 0.068 | 0.000 | 0.000 | 0.000 |
| 187 | *Lycopodium clavatum* | 0.000 | 0.000 | 0.000 | 0.505 | 0.250 | 0.000 | 0.000 |
| 188 | *Macaranga capensis* | 1.370 | 0.000 | 0.000 | 1.241 | 0.000 | 0.000 | 0.000 |
| 189 | *Maesa lanceolata* | 1.308 | 0.000 | 0.000 | 0.068 | 0.000 | 0.000 | 0.777 |
| 190 | *Manilkara butugi* | 0.000 | 4.769 | 0.000 | 0.000 | 0.000 | 0.000 | 0.444 |
| 191 | *Marattia fraxinea* | 0.294 | 0.000 | 0.000 | 0.344 | 0.000 | 0.000 | 0.000 |
| 192 | *Marantochloa leucantha* | 0.000 | 0.000 | 0.000 | 0.000 | 0.000 | 0.000 | 0.666 |
| 193 | *Maytenus arbutifolia* | 0.000 | 0.000 | 0.000 | 0.172 | 0.000 | 0.000 | 0.000 |
| 194 | *Maytenus gracilipes* | 0.117 | 0.000 | 0.000 | 0.793 | 0.000 | 0.000 | 0.000 |
| 195 | *Maytenus obscura* | 0.000 | 0.000 | 0.555 | 0.000 | 0.000 | 0.000 | 0.000 |
| 196 | *Maytenus undata* | 0.000 | 0.000 | 0.000 | 0.103 | 0.000 | 0.000 | 0.000 |
| 197 | *Mellera lobulata* | 0.000 | 0.384 | 0.000 | 0.103 | 0.000 | 0.000 | 0.000 |
| 198 | *Mikaniopsis clematoides* | 0.000 | 0.000 | 0.444 | 0.000 | 0.625 | 0.000 | 0.000 |
| 199 | *Milicia excelsa* | 0.000 | 0.769 | 0.000 | 0.000 | 0.000 | 0.000 | 0.000 |
| 200 | *Millettia ferruginea* | 0.823 | 0.000 | 0.000 | 0.551 | 0.000 | 0.000 | 2.111 |
| 201 | *Mimusops kummel* | 1.058 | 2.307 | 0.000 | 0.000 | 0.000 | 0.800 | 2.666 |
| 202 | *Momordica foetida* | 0.000 | 0.000 | 0.000 | 0.103 | 0.000 | 0.000 | 0.000 |
| 203 | *Morus mesozygia* | 2.000 | 1.923 | 0.000 | 0.000 | 0.000 | 4.200 | 4.888 |
| 204 | *Myrsine africana* | 0.117 | 0.000 | 0.000 | 0.000 | 0.000 | 0.000 | 0.000 |
| 205 | *Nephrolepis biserrata* | 0.058 | 0.076 | 0.000 | 0.034 | 0.500 | 0.000 | 0.000 |
| 206 | *Nuxia congesta* | 0.000 | 0.692 | 0.000 | 0.000 | 0.000 | 1.006 | 0.000 |
| 207 | *Ocimum grattissimum* | 0.000 | 0.000 | 0.000 | 0.034 | 0.000 | 0.000 | 0.000 |
| 208 | *Ocotea kenyensis* | 0.000 | 0.000 | 0.000 | 0.344 | 0.000 | 0.000 | 0.000 |
| 209 | *Olea capensis* subsp.*macrocarpa* | 0.411 | 0.000 | 0.000 | 1.241 | 0.000 | 0.000 | 0.666 |
| 210 | *Olea welwitschii* | 4.000 | 0.000 | 0.000 | 2.827 | 0.000 | 0.000 | 0.444 |
| 211 | *Oncinotis tenuiloba* | 0.588 | 0.000 | 0.000 | 0.413 | 0.000 | 0.801 | 0.444 |
| 212 | *Oncoba spinosa* | 0.000 | 0.153 | 0.000 | 0.000 | 0.000 | 0.000 | 0.000 |
| 213 | *Oplismenus hirtellus* | 1.176 | 0.000 | 6.000 | 1.517 | 1.125 | 0.000 | 1.111 |
| 214 | *Oryra latifolia* | 0.647 | 0.000 | 0.000 | 0.137 | 1.375 | 0.000 | 1.444 |
| 215 | *Oxalis radicosa* | 0.000 | 0.000 | 0.444 | 0.000 | 0.375 | 0.000 | 0.000 |
| 216 | *Oxyanthus speciosus* | 0.411 | 0.000 | 0.000 | 0.827 | 0.000 | 0.000 | 0.000 |
| 217 | *Panicum atrosanguineum* | 0.500 | 0.615 | 0.000 | 0.000 | 0.750 | 0.000 | 0.000 |
| 218 | *Panicum calvum* | 0.552 | 0.000 | 0.000 | 0.000 | 0.625 | 0.000 | 0.000 |
| 219 | *Paspalum scrobiculatum* | 0.000 | 0.153 | 0.000 | 0.000 | 0.875 | 0.000 | 0.000 |
| 220 | *Paullinia pinnata* | 0.604 | 0.000 | 0.000 | 0.068 | 0.000 | 1.220 | 0.444 |
| 221 | *Pavetta abyssinica* | 0.000 | 0.000 | 0.000 | 0.206 | 0.000 | 0.000 | 0.000 |
| 222 | *Pavetta oliveriana* | 0.000 | 0.000 | 0.000 | 0.137 | 0.000 | 0.000 | 0.000 |
| 223 | *Pellaea viridis* | 0.411 | 0.000 | 0.000 | 0.034 | 0.000 | 0.800 | 0.000 |
| 224 | *Pennisetum macroururn* | 0.000 | 0.000 | 0.000 | 0.000 | 2.250 | 0.000 | 2.222 |
| 225 | *Pennisetum trachyphyllum* | 0.000 | 0.000 | 0.000 | 0.471 | 0.588 | 0.000 | 0.222 |
| 226 | *Pentas lanceolata* | 0.000 | 0.000 | 0.000 | 0.034 | 0.000 | 0.000 | 0.000 |
| 227 | *Peperomia molleri* | 0.000 | 0.000 | 0.000 | 0.068 | 0.000 | 0.000 | 0.000 |
| 228 | *Peperomia retusa* | 0.000 | 0.000 | 0.000 | 0.068 | 0.000 | 0.000 | 0.000 |
| 229 | *Peponium vogelii* | 0.000 | 0.000 | 0.000 | 0.068 | 0.000 | 0.000 | 0.000 |
| 230 | *Persicaria setosula* | 0.000 | 0.000 | 0.000 | 0.000 | 0.625 | 0.000 | 0.000 |
| 231 | *Phaulopsis imbricata* | 0.000 | 0.307 | 0.000 | 0.103 | 0.000 | 0.000 | 0.000 |
| 232 | *Phoenix reclinata* | 0.176 | 0.384 | 0.000 | 0.275 | 0.000 | 3.200 | 3.444 |
| 233 | *Phyllanthus limmuensis* | 0.000 | 0.153 | 0.000 | 0.000 | 0.125 | 0.000 | 0.000 |
| 234 | *Phyllanthus ovalifolius* | 0.000 | 0.000 | 0.000 | 0.068 | 0.000 | 0.000 | 0.111 |
| 235 | *Physalis peruviana* | 0.000 | 0.000 | 0.000 | 0.000 | 0.000 | 0.200 | 0.111 |
| 236 | *Phytolacca dodecandra* | 0.117 | 0.000 | 0.000 | 0.000 | 0.000 | 0.000 | 0.000 |
| 237 | *Pilea rivularis* | 0.000 | 0.000 | 0.000 | 0.034 | 0.500 | 0.000 | 0.000 |
| 238 | *Piper capense* | 0.647 | 0.000 | 0.000 | 0.586 | 0.000 | 0.000 | 1.777 |
| 239 | *Piper umbellatum* | 0.000 | 0.000 | 0.000 | 0.000 | 0.000 | 0.000 | 0.666 |
| 240 | *Pittosporum viridiflorum* | 0.470 | 0.000 | 0.000 | 0.655 | 0.000 | 2.800 | 0.000 |
| 241 | *Plantago lanceolata* | 0.000 | 0.000 | 0.000 | 0.034 | 0.000 | 0.000 | 0.000 |
| 242 | *Plantago palmata* | 0.000 | 0.460 | 0.000 | 0.000 | 0.500 | 0.000 | 0.000 |
| 243 | *Plectranthus garckeanus* | 0.000 | 0.000 | 0.000 | 0.034 | 0.000 | 0.000 | 0.000 |
| 244 | *Plectranthus punctatus* | 0.117 | 0.000 | 0.000 | 0.000 | 0.000 | 0.000 | 0.222 |
| 245 | *Pollia condensata* | 0.000 | 0.000 | 0.000 | 0.000 | 0.000 | 0.200 | 0.222 |
| 246 | *Polyscias fulva* | 0.294 | 0.384 | 0.701 | 0.896 | 0.000 | 1.012 | 1.000 |
| 247 | *Pouteria adolfi-friederici* | 0.000 | 0.000 | 0.000 | 5.517 | 0.000 | 0.000 | 0.000 |
| 248 | *Pouteria alnifolia* | 0.000 | 1.538 | 0.000 | 0.000 | 0.000 | 0.000 | 0.000 |
| 249 | *Pouteria altissima* | 0.000 | 0.769 | 0.000 | 0.000 | 0.000 | 0.000 | 0.000 |
| 250 | *Premna schimperi* | 0.117 | 0.000 | 0.000 | 0.068 | 0.000 | 0.000 | 0.000 |
| 251 | *Prunus africana* | 0.000 | 0.000 | 1.725 | 0.689 | 0.000 | 0.000 | 0.000 |
| 252 | *Psidium guajava* | 0.000 | 0.000 | 0.000 | 0.000 | 0.000 | 0.400 | 0.333 |
| 253 | *Psychotria orophila* | 0.058 | 0.153 | 0.000 | 1.448 | 0.000 | 0.200 | 0.555 |
| 254 | *Pteris dentata* | 0.000 | 0.000 | 0.000 | 0.896 | 0.000 | 0.000 | 0.000 |
| 255 | *Pteris pteridioides* | 0.000 | 0.000 | 0.000 | 0.068 | 0.000 | 0.000 | 0.000 |
| 256 | *Pterolobium stellatum* | 0.000 | 0.000 | 0.000 | 0.379 | 0.000 | 0.000 | 0.222 |
| 257 | *Pycnostachys abyssinica* | 0.000 | 0.000 | 0.000 | 0.068 | 0.000 | 0.000 | 0.000 |
| 258 | *Ranunculus multifidus* | 0.000 | 0.000 | 0.000 | 0.000 | 0.625 | 0.000 | 0.000 |
| 259 | *Rhamnus prinoides* | 0.000 | 0.000 | 1.333 | 0.103 | 0.000 | 0.000 | 0.000 |
| 260 | *Rhus glutinosa* | 0.000 | 0.000 | 0.000 | 0.000 | 0.000 | 0.000 | 0.555 |
| 261 | *Rinorea friisii* | 0.000 | 0.615 | 0.000 | 0.000 | 0.000 | 0.000 | 0.000 |
| 262 | *Ritchiea albersii* | 0.000 | 0.000 | 0.000 | 0.000 | 0.000 | 1.340 | 3.111 |
| 263 | *Rothmannia urcelliformis* | 0.764 | 0.307 | 0.000 | 0.655 | 0.000 | 0.000 | 0.000 |
| 264 | *Rubus steudneri* | 0.000 | 0.000 | 0.000 | 0.137 | 0.000 | 0.000 | 0.000 |
| 265 | *Ruellia prostrata* | 0.000 | 0.000 | 0.000 | 0.241 | 0.000 | 0.200 | 0.000 |
| 266 | *Rumex abyssinicus* | 0.000 | 0.000 | 0.000 | 0.000 | 0.500 | 0.000 | 0.000 |
| 267 | *Rumex nepalensis* | 0.000 | 0.000 | 0.000 | 0.034 | 0.375 | 0.000 | 0.000 |
| 268 | *Rytigynia neglecta* | 0.580 | 0.307 | 0.000 | 0.931 | 0.000 | 0.000 | 0.000 |
| 269 | *Saba comorensis* | 0.000 | 1.000 | 0.000 | 0.000 | 0.000 | 1.200 | 0.000 |
| 270 | *Salvia nilotica* | 0.427 | 0.000 | 0.444 | 0.000 | 0.000 | 0.000 | 0.000 |
| 271 | *Sanicula elata* | 0.117 | 0.000 | 0.888 | 0.068 | 0.875 | 0.000 | 0.000 |
| 272 | *Sapium ellipticum* | 3.176 | 0.000 | 0.000 | 0.965 | 0.000 | 5.400 | 0.000 |
| 273 | *Satureja paradoxa* | 0.000 | 0.000 | 0.666 | 0.000 | 0.000 | 0.000 | 0.000 |
| 274 | *Scadoxus nutans* | 0.000 | 0.000 | 0.000 | 0.310 | 0.000 | 0.000 | 0.000 |
| 275 | *Schefflera abyssinica* | 1.176 | 0.000 | 0.000 | 4.441 | 0.000 | 0.000 | 2.333 |
| 276 | *Schefflera myriantha* | 0.000 | 0.000 | 2.033 | 0.172 | 0.000 | 0.000 | 0.000 |
| 277 | *Schefflera volkensii* | 0.000 | 0.000 | 2.182 | 1.517 | 0.000 | 0.000 | 0.000 |
| 278 | *Selaginella kraussiana* | 0.000 | 0.000 | 0.000 | 0.068 | 0.000 | 0.000 | 0.000 |
| 279 | *Senna petersiana* | 0.117 | 0.000 | 0.000 | 0.000 | 0.000 | 0.523 | 0.000 |
| 280 | *Senna septemtrionali* | 0.000 | 0.000 | 0.000 | 0.068 | 0.000 | 0.600 | 0.000 |
| 281 | *Setaria megaphylla* | 0.000 | 0.000 | 0.000 | 0.068 | 1.625 | 0.000 | 2.444 |
| 282 | *Smilax anceps* | 0.000 | 0.000 | 0.000 | 0.000 | 0.000 | 0.000 | 0.111 |
| 283 | *Smilax aspera* | 0.000 | 0.000 | 0.000 | 0.034 | 0.000 | 0.000 | 0.000 |
| 284 | *Snowdenia polystachya* | 0.000 | 0.000 | 0.000 | 0.000 | 0.750 | 0.000 | 0.000 |
| 285 | *Solanecio gigas* | 0.294 | 0.000 | 0.000 | 0.137 | 0.000 | 0.000 | 0.000 |
| 286 | *Solanecio mannii* | 0.058 | 0.000 | 0.000 | 0.103 | 0.000 | 0.000 | 0.000 |
| 287 | *Solanum incanum* | 0.000 | 0.000 | 0.000 | 0.068 | 0.000 | 0.000 | 0.000 |
| 288 | *Sonchus bipontini* | 0.000 | 0.000 | 0.000 | 0.137 | 0.000 | 0.000 | 0.000 |
| 289 | *Sporobolus pyramidalis* | 0.000 | 0.000 | 0.000 | 0.000 | 1.500 | 0.000 | 0.000 |
| 290 | *Stellaria sennii* | 0.000 | 0.000 | 0.000 | 0.000 | 0.125 | 0.400 | 0.000 |
| 291 | *Stephania abyssinica* | 0.000 | 0.000 | 0.000 | 0.068 | 0.000 | 0.000 | 0.000 |
| 292 | *Stereospermum kunthianum* | 0.000 | 0.692 | 0.000 | 0.000 | 0.000 | 1.000 | 0.666 |
| 293 | *Stiotocardia beraviensis* | 0.000 | 0.153 | 0.000 | 0.000 | 0.000 | 0.000 | 0.000 |
| 294 | *Strychnos mitis* | 0.000 | 2.461 | 0.000 | 0.000 | 0.000 | 0.000 | 0.511 |
| 295 | *Syzygium guineense* Subsp*. afromontanum* | 0.764 | 0.000 | 1.602 | 2.931 | 0.000 | 0.000 | 0.000 |
| 296 | *Tagetes minuta* | 0.000 | 0.000 | 0.333 | 0.000 | 0.000 | 0.000 | 0.000 |
| 297 | *Teclea noblis* | 0.504 | 1.076 | 0.000 | 0.517 | 0.000 | 2.600 | 0.000 |
| 298 | *Tectaria gemmifera* | 1.000 | 0.000 | 0.000 | 1.137 | 0.000 | 0.000 | 0.000 |
| 299 | *Terminalia schimperiana* | 0.000 | 0.384 | 0.000 | 0.000 | 0.000 | 0.000 | 0.000 |
| 300 | *Thalictrum rhynchocarpum* | 0.000 | 0.000 | 0.000 | 0.344 | 0.000 | 0.000 | 0.777 |
| 301 | *Thunbergia alata* | 0.058 | 0.230 | 0.410 | 0.000 | 0.000 | 0.000 | 0.000 |
| 302 | *Tiliacora troupinii* | 0.401 | 0.000 | 0.000 | 0.620 | 0.000 | 0.511 | 0.333 |
| 303 | *Trema orientalis* | 1.117 | 0.230 | 0.000 | 0.000 | 0.000 | 1.430 | 0.000 |
| 304 | *Trichilia dregeana* | 1.470 | 0.923 | 0.000 | 0.000 | 0.000 | 4.800 | 3.777 |
| 305 | *Trifolium baccarinii* | 0.000 | 0.000 | 0.000 | 0.068 | 0.000 | 0.000 | 0.000 |
| 306 | *Trifolium mattirolianum* | 0.000 | 0.000 | 0.111 | 0.275 | 0.000 | 0.000 | 0.000 |
| 307 | *Trifolium polystachyum* | 0.000 | 0.000 | 0.444 | 0.000 | 0.500 | 0.000 | 0.000 |
| 308 | *Trilepisium madagascariense* | 1.058 | 1.307 | 0.000 | 0.137 | 0.000 | 1.600 | 5.000 |
| 309 | *Tristemma mauritianum* | 0.000 | 0.000 | 0.333 | 0.421 | 1.750 | 0.000 | 0.000 |
| 310 | *Turraea holstii* | 0.000 | 0.846 | 0.000 | 0.000 | 0.000 | 0.000 | 0.622 |
| 311 | *Tylophora sylvatica* | 0.000 | 0.000 | 0.000 | 0.000 | 0.000 | 0.000 | 0.555 |
| 312 | *Urera hypselodendron* | 0.705 | 0.384 | 0.000 | 0.241 | 0.000 | 0.000 | 0.444 |
| 313 | *Urtica simensis* | 0.000 | 0.000 | 0.000 | 0.034 | 0.000 | 0.000 | 0.555 |
| 314 | *Vangueria madagascariensis* | 0.000 | 0.538 | 0.000 | 0.137 | 0.000 | 0.000 | 0.000 |
| 315 | *Vepris dainellii* | 1.941 | 0.000 | 0.000 | 3.034 | 0.000 | 3.600 | 0.000 |
| 316 | *Verbena officinalis* | 0.000 | 0.000 | 0.000 | 0.034 | 0.000 | 0.000 | 0.000 |
| 317 | *Vernonia amygdalina* | 0.000 | 0.000 | 0.000 | 0.103 | 0.000 | 0.000 | 0.000 |
| 318 | *Vernonia auriculifera* | 0.000 | 0.000 | 0.000 | 0.344 | 0.000 | 0.000 | 0.000 |
| 319 | *Vernonia hochstetteri* | 0.000 | 0.000 | 0.000 | 0.137 | 0.000 | 0.000 | 0.000 |
| 320 | *Vernonia karaguensis* | 0.000 | 0.000 | 0.000 | 0.034 | 0.000 | 0.000 | 0.000 |
| 321 | *Vernonia leopoldi* | 0.000 | 0.000 | 0.000 | 0.103 | 0.000 | 0.000 | 0.000 |
| 322 | *Veronica abyssinica* | 0.000 | 0.000 | 0.444 | 0.000 | 0.000 | 0.000 | 0.000 |
| 323 | *Vigna membranacea* | 0.000 | 0.000 | 0.000 | 0.068 | 0.000 | 0.000 | 0.000 |
| 324 | *Vigna vexillata* | 0.000 | 0.076 | 0.000 | 0.034 | 0.000 | 0.200 | 0.000 |
| 325 | *Whitfieldia elongata* | 0.000 | 1.769 | 0.000 | 0.000 | 0.000 | 0.000 | 0.000 |
| 326 | *Zanthoxylum usambarense* | 0.000 | 1.923 | 0.000 | 0.000 | 0.000 | 0.000 | 0.000 |
| 327 | *Zehneria scabra* | 0.000 | 0.000 | 0.444 | 0.034 | 0.000 | 0.000 | 0.000 |
