# Supplementary material for: Plant diversity and community analysis of Sele-Nono forest, Southwest Ethiopia: implication for conservation planning
Source: Bot Stud. 2022 Jul 19;63:23. doi: 10.1186/s40529-022-00353-w (PMC9294133; doi:10.1186/s40529-022-00353-w)
Supplement: Supplementary file 10 — Additional file 10: Appendix S10. Enviromental data recorded for each sample plot in Sele-Nono forest. [file 40529_2022_353_MOESM10_ESM.doc]

Appendix 1. Enviromental data recorded for each sample plot in Sele-Nono forest

| **Plot No** | **Altitude (m)** | **Slope (^o^)** | **Aspect** | **Disturbance** | **pH (H20)** | **P (ppm)** | **EC (mm/cm)** | **OM (%)** | **K (meq/100gm)** | **CEC (meq/100gm)** | **TN**  **(%)** | **Sand (%)** | **Silt**  **(%)** | **Clay (%)** |
| --- | --- | --- | --- | --- | --- | --- | --- | --- | --- | --- | --- | --- | --- | --- |
| 1 | 1640 | 33 | 1 | 2 | 4.7 | 6.75 | 0.32 | 11.06 | 0.72 | 54.62 | 0.65 | 25 | 43.14 | 31.86 |
| 2 | 1165 | 25 | 0 | 4 | 5.64 | 8.53 | 0.563 | 7.293 | 1.34 | 29.22 | 0.34 | 25 | 36 | 39 |
| 3 | 1640 | 42 | 2 | 1 | 4.88 | 5.12 | 0.23 | 9.431 | 0.63 | 74.23 | 0.75 | 35 | 35 | 30 |
| 4 | 2400 | 20 | 1.3 | 2 | 4.76 | 3.23 | 0.185 | 5.913 | 0.46 | 90.2 | 0.94 | 32.5 | 42.75 | 24.75 |
| 5 | 2240 | 40 | 2 | 1 | 4.89 | 4.74 | 0.253 | 6.189 | 0.46 | 72.17 | 0.87 | 32 | 41.25 | 26.75 |
| 6 | 1640 | 0 | 0 | 3 | 4.4 | 4.63 | 0.204 | 15.58 | 0.46 | 30.1 | 1.02 | 37.5 | 41.25 | 21.25 |
| 7 | 2430 | 12 | 0 | 2 | 4.89 | 3.93 | 0.211 | 7.396 | 0.28 | 84.29 | 0.56 | 32.5 | 40 | 27.5 |
| 8 | 1872 | 35 | 1 | 3 | 4.98 | 5.94 | 0.37 | 11.70 | 0.69 | 65.97 | 0.52 | 25 | 37 | 38 |
| 9 | 2180 | 36 | 2 | 1 | 5.21 | 4.03 | 0.132 | 10.39 | 0.32 | 83.21 | 0.96 | 26 | 41.25 | 32.75 |
| 10 | 1740 | 0 | 0 | 3 | 5.93 | 6.98 | 0.254 | 18.55 | 0.32 | 40 | 1.03 | 37.5 | 41.25 | 21.25 |
| 11 | 1620 | 14 | 2.5 | 5 | 5.61 | 5.6 | 0.389 | 12.36 | 0.89 | 58.32 | 0.53 | 34.11 | 32.23 | 33.66 |
| 12 | 1830 | 0 | 0 | 3 | 5.55 | 3.93 | 0.379 | 5.551 | 0.54 | 29.22 | 1 | 40 | 35 | 25 |
| 13 | 2380 | 15 | 1.3 | 5 | 4.24 | 4.74 | 0.282 | 19.62 | 0.22 | 89.04 | 0.87 | 40 | 38.75 | 21.25 |
| 14 | 1430 | 23 | 3 | 3 | 5.42 | 6.73 | 0.43 | 15.89 | 0.87 | 54.12 | 0.44 | 28.48 | 37.35 | 34.17 |
| 15 | 1850 | 0 | 0 | 3 | 5.18 | 4.74 | 0.452 | 14.51 | 0.43 | 51.56 | 1.04 | 32.5 | 40 | 27.5 |
| 16 | 1640 | 0 | 0 | 3 | 4.89 | 4.21 | 0.329 | 5.224 | 0.46 | 34.56 | 1.05 | 32.5 | 42.75 | 24.75 |
| 17 | 1230 | 29 | 3 | 4 | 6.27 | 7.12 | 0.698 | 16.29 | 0.98 | 51.56 | 0.29 | 40 | 35 | 25 |
| 18 | 1280 | 25 | 2 | 4 | 5.15 | 6.98 | 0.45 | 15.89 | 0.83 | 56.42 | 0.47 | 40 | 38.75 | 21.25 |
| 19 | 1880 | 48 | 1 | 1 | 5.61 | 4.54 | 0.148 | 9.706 | 0.54 | 79.11 | 0.63 | 27 | 35 | 38 |
| 20 | 1100 | 34 | 3 | 4 | 5.42 | 10.11 | 0.474 | 14.01 | 0.96 | 34.56 | 0.32 | 49.34 | 26.44 | 24.22 |
| 21 | 1860 | 38 | 2 | 2 | 5.64 | 4.12 | 0.158 | 11.29 | 0.54 | 83.29 | 0.72 | 32 | 40 | 28 |
| 22 | 1200 | 30 | 1.3 | 3 | 5.15 | 8.8 | 0.541 | 13.98 | 1.04 | 38.42 | 0.3 | 50.94 | 21 | 28.06 |
| 23 | 1820 | 45 | 0 | 3 | 4.89 | 5.43 | 0.26 | 11.43 | 0.62 | 77.34 | 0.54 | 23 | 40 | 37 |
| 24 | 2010 | 40 | 3 | 1 | 5.21 | 4.48 | 0.261 | 7.396 | 0.72 | 56.12 | 0.7 | 22 | 42.75 | 35.25 |
| 25 | 1600 | 35 | 1 | 2 | 4.73 | 5.37 | 0.236 | 12.43 | 0.74 | 70.52 | 0.75 | 35 | 40 | 25 |
| 26 | 1190 | 36 | 0 | 4 | 6.16 | 7.51 | 0.53 | 3.741 | 1.34 | 34.8 | 0.43 | 55.43 | 24.13 | 20.44 |
| 27 | 940 | 35 | 0 | 3 | 5.66 | 8.05 | 0.499 | 8.017 | 1.43 | 44.22 | 0.37 | 46.34 | 32.5 | 21.16 |
| 28 | 1636 | 32 | 1 | 2 | 4.89 | 5.2 | 0.231 | 5.724 | 0.66 | 79.34 | 0.66 | 54 | 24 | 22 |
| 29 | 1900 | 46 | 3 | 3 | 5.42 | 5.03 | 0.184 | 10.08 | 0.36 | 69.25 | 0.88 | 31 | 40 | 29 |
| 30 | 1250 | 27 | 1.3 | 4 | 6.13 | 9.6 | 0.518 | 7.068 | 0.97 | 45.32 | 0.39 | 37.5 | 33 | 29.5 |
| 31 | 2300 | 18 | 0 | 2 | 4.11 | 4.21 | 0.203 | 10.20 | 0.29 | 89.89 | 1.02 | 28.75 | 45 | 26.25 |
| 32 | 1905 | 30 | 3 | 2 | 4.89 | 4.87 | 0.232 | 7.862 | 0.42 | 73.11 | 0.58 | 27.23 | 38.75 | 34.02 |
| 33 | 1800 | 18 | 2.5 | 5 | 5.21 | 5.87 | 0.352 | 11.29 | 0.72 | 68.13 | 0.55 | 36.04 | 28.17 | 35.79 |
| 34 | 1885 | 40 | 2 | 2 | 4.37 | 5.73 | 0.167 | 9.948 | 0.43 | 86.34 | 0.96 | 20 | 45 | 35 |
| 35 | 1350 | 28 | 3.3 | 3 | 5.45 | 6.66 | 0.48 | 10.27 | 0.94 | 63.24 | 0.38 | 45 | 38.75 | 16.25 |
| 36 | 1394 | 40 | 1.3 | 3 | 4.89 | 5.36 | 0.21 | 16.34 | 0.53 | 68.46 | 0.56 | 37 | 46 | 17 |
| 37 | 2070 | 35 | 2 | 2 | 4.89 | 5.64 | 0.261 | 9.137 | 0.46 | 90.01 | 1.01 | 25 | 42.75 | 32.25 |
| 38 | 1625 | 36 | 0 | 2 | 4.76 | 4.67 | 0.13 | 11.53 | 0.64 | 64.88 | 0.5 | 43 | 35 | 22 |
| 39 | 1500 | 12 | 3 | 4 | 5.66 | 5.98 | 0.42 | 14.18 | 1.01 | 48.63 | 0.48 | 37.5 | 41.25 | 21.25 |
| 40 | 2350 | 22 | 1.3 | 2 | 4.04 | 4.44 | 0.186 | 7.931 | 0.25 | 84.47 | 0.96 | 47.5 | 38.75 | 13.75 |
| 41 | 1900 | 33 | 1.3 | 2 | 4.89 | 4.58 | 0.25 | 11 | 0.69 | 74.38 | 0.72 | 25 | 40 | 35 |
| 42 | 1600 | 35 | 4 | 1 | 5.12 | 3.87 | 0.375 | 12.36 | 0.61 | 82.38 | 0.7 | 32.5 | 42.75 | 24.75 |
| 43 | 1840 | 0 | 0 | 3 | 4.99 | 5.4 | 0.318 | 16.10 | 1.11 | 51.56 | 0.94 | 32.5 | 40 | 27.5 |
| 44 | 1630 | 20 | 3.3 | 4 | 5.61 | 6.22 | 0.32 | 13.51 | 0.73 | 64.26 | 0.5 | 37.83 | 27.25 | 34.92 |
| 45 | 1865 | 43 | 2 | 2 | 4.65 | 4.32 | 0.188 | 7.620 | 0.29 | 74.11 | 0.75 | 22 | 40 | 38 |
| 46 | 1910 | 26 | 1.3 | 4 | 4.45 | 4.23 | 0.217 | 6.448 | 0.36 | 88.49 | 0.98 | 31.25 | 42.5 | 26.25 |
| 47 | 2353 | 44 | 0 | 2 | 5.04 | 5.22 | 0.38 | 9.948 | 0.72 | 74.55 | 0.64 | 32.5 | 40 | 27.5 |
| 48 | 1645 | 48 | 3 | 2 | 4.4 | 4.05 | 0.211 | 7.396 | 0.52 | 74.32 | 0.94 | 24 | 38.75 | 37.25 |
| 49 | 2272 | 35 | 3 | 1 | 4.6 | 4.74 | 0.282 | 10.08 | 0.43 | 74.51 | 0.74 | 28.75 | 45 | 26.25 |
| 50 | 1655 | 0 | 0 | 3 | 4.89 | 4.67 | 0.1536 | 2.120 | 0.96 | 74.38 | 1.03 | 40 | 38.75 | 21.25 |
| 51 | 2120 | 35 | 3 | 1 | 5.55 | 4.21 | 0.176 | 10.20 | 0.26 | 80.54 | 0.67 | 37 | 38.75 | 24.25 |
| 52 | 1420 | 35 | 3 | 3 | 6.16 | 6.8 | 0.436 | 14.56 | 0.82 | 63.6 | 0.52 | 37.5 | 41.25 | 21.25 |
| 53 | 1160 | 23 | 0 | 2 | 5.52 | 6.98 | 0.467 | 14.36 | 1.23 | 52.31 | 0.33 | 55 | 21 | 24 |
| 54 | 2163 | 35 | 3 | 1 | 4.14 | 5.76 | 0.127 | 7.931 | 0.46 | 82.56 | 0.72 | 31.25 | 42.5 | 26.25 |
| 55 | 1800 | 25 | 1 | 5 | 4.89 | 5.79 | 0.23 | 9.586 | 0.51 | 80.55 | 0.72 | 22 | 37.5 | 40.5 |
| 56 | 1895 | 15 | 1.3 | 5 | 5.64 | 7.51 | 0.38 | 13.18 | 0.68 | 59.46 | 0.57 | 45 | 24.21 | 30.79 |
| 57 | 2083 | 25 | 1 | 1 | 4.52 | 4.52 | 0.183 | 9.172 | 0.42 | 76.05 | 0.86 | 28 | 46 | 26 |
| 58 | 2415 | 14 | 2.5 | 5 | 4.11 | 4.3 | 0.165 | 6.793 | 0.26 | 90.32 | 0.96 | 32.5 | 37.5 | 30 |
| 59 | 1126 | 26 | 0 | 4 | 5.91 | 8.23 | 0.483 | 5.879 | 1.11 | 42.43 | 0.35 | 41.36 | 32.11 | 26.53 |
| 60 | 2058 | 38 | 1 | 1 | 4.89 | 3.93 | 0.235 | 10.27 | 0.46 | 83 | 0.84 | 45 | 38.75 | 16.25 |
| 61 | 2100 | 25 | 2 | 1 | 4.93 | 3.53 | 0.143 | 7.793 | 0.43 | 82.38 | 0.73 | 22.5 | 32.5 | 45 |
| 62 | 2160 | 25 | 2 | 1 | 5.61 | 4.21 | 0.185 | 7.982 | 0.46 | 78.85 | 0.7 | 37.5 | 37.5 | 25 |
| 63 | 1865 | 40 | 0 | 2 | 5.93 | 4.88 | 0.32 | 11.44 | 0.64 | 72.59 | 0.63 | 32 | 38.75 | 29.25 |
| 64 | 1870 | 35 | 2 | 1 | 4.89 | 3.17 | 0.154 | 9.413 | 0.29 | 90.32 | 0.74 | 55 | 30 | 15 |
| 65 | 1220 | 30 | 1 | 4 | 5.08 | 5.41 | 0.218 | 11.29 | 0.53 | 68.41 | 0.6 | 28.75 | 45 | 26.25 |
| 66 | 2040 | 16 | 1.3 | 3 | 4.89 | 4.12 | 0.175 | 7.965 | 0.38 | 74.38 | 1.01 | 50 | 11 | 39 |
| 67 | 1634 | 35 | 2 | 2 | 4.41 | 4.34 | 0.264 | 7.482 | 0.37 | 67.91 | 0.69 | 32.5 | 40 | 27.5 |
| 68 | 2095 | 35 | 1 | 2 | 5.18 | 4.74 | 0.267 | 6.103 | 0.51 | 77.04 | 0.65 | 28 | 38.75 | 33.25 |
| 69 | 2344 | 34 | 2 | 2 | 4.89 | 3.88 | 0.159 | 8.603 | 0.42 | 74.4 | 0.72 | 32.5 | 42.75 | 24.75 |
| 70 | 1870 | 40 | 2 | 2 | 5.52 | 6.12 | 0.416 | 4.379 | 0.85 | 58.36 | 0.46 | 40 | 35 | 25 |
| 71 | 1880 | 38 | 1 | 2 | 4.89 | 5.71 | 0.261 | 6.137 | 0.62 | 74.58 | 0.63 | 31.25 | 42.5 | 26.25 |
| 72 | 2150 | 32 | 1 | 3 | 6.22 | 4.74 | 0.546 | 6.672 | 0.96 | 29.44 | 0.45 | 58.01 | 30.33 | 11.66 |
| 73 | 1276 | 41 | 1 | 2 | 4.99 | 4.46 | 0.25 | 14.51 | 0.7 | 78.52 | 0.72 | 18 | 37.5 | 44.5 |
| 74 | 1600 | 33 | 0 | 4 | 4.73 | 5.4 | 0.473 | 17.87 | 0.79 | 35.06 | 0.42 | 41.24 | 20 | 38.76 |
| 75 | 1160 | 46 | 2 | 3 | 4.89 | 4.05 | 0.166 | 6.189 | 0.46 | 96.47 | 0.83 | 32.5 | 40 | 27.5 |
| 76 | 1390 | 30 | 2 | 2 | 4.37 | 3.86 | 0.158 | 17.87 | 0.3 | 85.06 | 0.8 | 40 | 38.75 | 21.25 |
| 77 | 1134 | 25 | 1.3 | 3 | 4.89 | 4.92 | 0.256 | 11.51 | 0.65 | 68.33 | 0.5 | 26 | 34 | 40 |
| 78 | 2041 | 30 | 2 | 2 | 5.21 | 4.74 | 0.282 | 11.94 | 0.55 | 72.37 | 0.58 | 46 | 31 | 23 |
| 79 | 2020 | 25 | 2 | 1 | 4.89 | 4.75 | 0.286 | 10.03 | 0.47 | 90.25 | 0.78 | 33 | 45 | 22 |
| 80 | 1900 | 35 | 0 | 3 | 5.21 | 7.27 | 0.261 | 13.98 | 0.79 | 82.38 | 0.94 | 28.75 | 45 | 26.25 |
| 81 | 1640 | 32 | 1.3 | 2 | 5.61 | 5.23 | 0.315 | 11.06 | 0.68 | 68.38 | 0.68 | 37.5 | 41.25 | 21.25 |
| 82 | 2018 | 28 | 0 | 3 | 4.89 | 8.6 | 0.557 | 8.241 | 0.91 | 22.92 | 0.36 | 51.24 | 18.42 | 30.34 |
| 83 | 1850 | 0 | 0 | 3 | 4.28 | 5.72 | 0.234 | 11.29 | 0.32 | 74.38 | 0.75 | 31 | 42.75 | 26.25 |
| 84 | 1887 | 50 | 2 | 1 | 4.55 | 5.34 | 0.211 | 7.396 | 0.52 | 82.38 | 0.73 | 29 | 40 | 31 |
| 85 | 1760 | 25 | 3 | 5 | 4.89 | 4.21 | 0.284 | 10.08 | 0.44 | 83.61 | 0.83 | 40 | 38.75 | 21.25 |
| 86 | 2090 | 28 | 2.5 | 4 | 5.21 | 4.74 | 0.325 | 12.36 | 0.81 | 69.36 | 0.51 | 33.41 | 32.46 | 34.13 |
| 87 | 1420 | 15 | 3 | 3 | 6.05 | 5.86 | 0.403 | 16.29 | 0.93 | 35.06 | 0.42 | 32.5 | 40 | 27.5 |
| 88 | 2100 | 35 | 1 | 1 | 4.62 | 5.36 | 0.209 | 7.465 | 0.42 | 85.62 | 0.72 | 45 | 38.75 | 16.25 |
| 89 | 2440 | 12 | 1.3 | 2 | 4.03 | 4.64 | 0.379 | 7.310 | 0.42 | 82.38 | 0.75 | 45 | 38.75 | 16.25 |
| 90 | 1190 | 25 | 0 | 4 | 4.89 | 5.79 | 0.462 | 14.62 | 1.43 | 35.12 | 0.3 | 42.32 | 29.25 | 28.43 |
